# Molecularly Targeted Therapies in Oncology: Mechanisms, Resistance, and Combination Strategies

**DOI:** 10.3390/molecules31071195

**Published:** 2026-04-03

**Authors:** Klaudia Giercuszkiewicz-Haśnik, Beata Morak-Młodawska, Małgorzata Jeleń

**Affiliations:** 1 Department of Systems Biology and Engineering, Silesian University of Technology, 44-100 Gliwice, Poland; 2 Centre of Biotechnology, Silesian University of Technology, 44-100 Gliwice, Poland; 3 Faculty of Medical Sciences in Katowice, Medical University of Silesia, 40-752 Katowice, Poland; 4 Department of Organic Chemistry, Faculty of Pharmaceutical Sciences in Sosnowiec, Medical University of Silesia in Katowice, 41-200 Sosnowiec, Poland; bmlodawska@sum.edu.pl

**Keywords:** targeted therapy, precision oncology, combination therapy, immuno-oncology, cancer drug discovery, molecular oncology

## Abstract

Targeted therapies are reshaping oncology by enabling treatment selection based on actionable molecular alterations, improving precision, and reducing unnecessary toxicity. This review provides an up-to-date overview of current targeted treatment modalities and the medicinal chemistry principles that support their discovery and optimization. We synthesize evidence on small-molecule and biologic strategies spanning receptor and non-receptor kinases and their major signaling axes (PI3K-AKT-mTOR and RAS-RAF-MEK-ERK), apoptosis regulation (BCL-2 family), DNA repair via poly(ADP-ribose) polymerase (PARP) inhibition, and epigenetic or metabolic targets including histone deacetylases (HDACs), bromodomain and extra-terminal proteins (BET), and mutant isocitrate dehydrogenases (IDH1/2). Across these areas, we summarize recurrent resistance mechanisms and the rationale for combination or sequential approaches. Biologic targeted therapy is discussed in parallel, including immune checkpoint blockade, antibody–drug conjugates, bispecific antibodies (BsAb), and cell therapies such as chimeric antigen receptor T cells, with emphasis on biomarker-guided patient stratification. Finally, we outline emerging directions beyond canonical nodes, including modulation of the p53-MDM2/MDM4 axis, ferroptosis control through AIFM2/FSP1, and innate immune pathways such as CD47-SIRPa and the stimulator of interferon genes (STING). Overall, the field is shifting from single-target inhibition toward integrated strategies that combine precise molecular targeting with an understanding of signaling network dynamics, resistance evolution, and therapeutic vulnerabilities.

## 1. Introduction

Targeted therapies are a major pillar of modern oncology, as treatments guided by specific biomarkers can improve clinical outcomes compared with standard, non-selective anticancer approaches [1,2]. Unlike conventional strategies, targeted interventions are built around defined molecular alterations, enabling a more precise selection of therapy and, consequently, reduced toxicity in selected patient groups while improving therapeutic success—particularly in patients carrying so-called driver mutations [1]. According to recent reviews, the greatest benefits have been observed in cancers for which clear therapeutic targets have been identified and reliable diagnostic tests have been developed to detect them. In everyday clinical practice, this translates into the use of monoclonal antibodies, immunotherapy, small-molecule targeted agents, or antibody–drug conjugates, which can be better aligned with tumor biology than classical chemotherapy [3].

Progress in this field has also reshaped the design of clinical trials, with basket and umbrella frameworks increasingly used to evaluate drug efficacy across multiple molecular subgroups [4]. Basket trials assess the activity of a given drug in tumors of different histological origin that share the same molecular alteration—for example, testing NTRK (neurotrophic tyrosine receptor kinase) inhibitors in cancers harboring NTRK gene fusions (a rare event in which an NTRK gene fragment fuses with another gene, resulting in a constitutively active TRK (tropomyosin receptor kinase) fusion protein that drives tumorigenesis and can be effectively targeted across various tumor types with TRK inhibitors) [4]. By contrast, umbrella trials focus on patients with the same tumor type but different genetic mutations or biomarkers; each subgroup receives a different targeted therapy matched to the underlying molecular abnormality [5,6]. A representative example is an umbrella study in non-small cell lung cancer (NSCLC) in the neoadjuvant setting, in which patients are assigned to targeted therapy based on the tumor’s molecular profile. This approach enables initiation of tailored treatment prior to surgery, thereby increasing the likelihood of effective disease control [7].

The development of targeted therapies would not have been possible without advances in medicinal chemistry and organic synthesis. The design of new ligands, structure–activity relationship (SAR) analyses, and pharmacokinetic optimization have enabled the generation of effective kinase inhibitors, epigenetic regulators, and agents that modulate apoptosis [2,8,9]. In parallel, computer-assisted approaches—such as molecular modeling—and artificial intelligence algorithms are playing an increasingly important role by accelerating the identification of drug candidates and supporting the selection of molecules for further preclinical evaluation [3].

In this review, we examine targeted anticancer therapies not only as separate drug classes, but also through several recurring developmental principles that shape modern oncology drug discovery. These include structure-guided optimization of target engagement, resistance-driven redesign of next-generation inhibitors, and the increasing use of rational combinations and biomarker-guided treatment selection. Using this framework, we compare small-molecule and biologic strategies across classical signaling nodes, apoptosis regulation, DNA repair, epigenetic control, and emerging non-canonical targets. Our aim is not only to summarize representative agents, but also to highlight why some therapeutic concepts achieved durable clinical relevance, whereas others were constrained by toxicity, limited selectivity, or rapid adaptive escape.

Taken together, targeted therapies can be conceptually understood within three interrelated dimensions: oncogenic pathway dependency, which determines initial therapeutic sensitivity; adaptive resistance mechanisms, including signaling rewiring and tumor heterogeneity; and therapeutic strategies aimed at overcoming these limitations, particularly combination and sequential treatments. This framework provides a unifying perspective for interpreting the diverse classes of targeted agents discussed in this review.

## 2. Classical Molecular Targets

### 2.1. Tyrosine Kinases and Signaling Pathways

Protein kinases are enzymes that transfer a phosphate group from adenosine triphosphate (ATP) to protein substrates, thereby regulating key cellular signaling pathways. Within targeted cancer therapy, tyrosine kinases (TKs), which catalyze the phosphorylation of tyrosine (Tyr) residues, and serine/threonine kinases, which catalyze the phosphorylation of serine and/or threonine (Ser/Thr) residues, are of particular importance. These enzymes control major oncogenic signaling routes, including the RAS-RAF-MEK-ERK axis and the phosphatidylinositol 3-kinase (PI3K)/AKT/mechanistic target of **rapamycin** (mTOR) pathway [10]. Tyrosine kinases are commonly classified into receptor tyrosine kinases (RTKs)—transmembrane proteins with an extracellular ligand-binding domain—and non-receptor tyrosine kinases (NRTKs), which are intracellular kinases lacking both an extracellular domain and a transmembrane segment [10]. Among NRTKs, the most clinically established targets include ABL (including the BCR-ABL fusion oncoprotein), Bruton’s tyrosine kinase (BTK; TEC family), and Janus kinases (JAKs), while focal adhesion kinase (FAK), targeted by defactinib, represents a more recent example [11]. RTKs form a large family of membrane receptors that serve as central signaling hubs in cancer cells (e.g., epidermal growth factor receptor (EGFR), human epidermal growth factor receptor 2 (HER2), mesenchymal–epithelial transition factor (MET), anaplastic lymphoma kinase (ALK), ROS proto-oncogene 1 (ROS1), rearranged during transfection (RET), vascular endothelial growth factor receptors (VEGFRs), fibroblast growth factor receptors (FGFRs), platelet-derived growth factor receptors (PDGFRs), KIT proto-oncogene receptor tyrosine kinase (KIT), and AXL receptor tyrosine kinase (AXL)). Physiologically, ligand binding (e.g., growth factors) induces receptor dimerization and autophosphorylation, which activates downstream signaling cascades controlling proliferation, differentiation, survival, and migration, including the JAK-STAT, RAS-RAF-MEK-ERK, and PI3K-AKT-mTOR pathways [12,13]. In cancer, RTKs are frequently deregulated through overexpression, activating mutations, or gene fusions, leading to oncogene addiction: tumor cells become dependent on a single hyperactive RTK-driven signaling route (the receptor tyrosine kinase (RTK) pathway), which fuels uncontrolled tumor growth [13,14]. For this reason, RTKs have been among the most important molecular targets in oncology for over two decades, and their pharmacological inhibition has transformed the treatment of multiple malignancies [15,16]. In parallel to RTK-dependent signaling, non-kinase pathways also contribute substantially to the regulation of cancer cell proliferation and differentiation. One prominent example is the Notch signaling pathway (NOTCH), which can be pharmacologically modulated, among others, by γ-secretase inhibitors (GSIs) [17]. Notch signaling has a context-dependent and multifaceted role in cancer, acting as either an oncogenic driver or a tumor suppressor. It influences proliferation, invasion and metastasis, epithelial-to-mesenchymal transition (EMT), angiogenesis, cancer stem cell properties, metabolic reprogramming, and the development of treatment resistance [17].

In NSCLC, treatment is now closely guided by molecular profiling. Clinicians routinely identify activating EGFR mutations, ALK rearrangements, ROS1 rearrangements, RET rearrangements, as well as mutations in BRAF, MET, and HER2, and NTRK fusion oncogenes, and then select appropriate kinase inhibitors. This strategy has translated into a significant survival improvement compared with chemotherapy [18,19,20]. A similar biomarker-driven approach applies to HER2 in breast and gastric cancers, KIT and platelet-derived growth factor receptor alpha (PDGFRA) in gastrointestinal stromal tumors (GISTs), BCR-ABL in chronic myeloid leukemia (CML), and vascular endothelial VEGFRs/PDGFRs in solid tumors requiring anti-angiogenic treatment [13,16,21]. Across many solid and hematologic malignancies, RTK blockade has become part of standard management, and new molecules, including covalent inhibitors and agents selective for rare mutations, continue to expand the spectrum of actionable indications [15]. RTKs transmit signals mainly through two major cascades: the PI3K/AKT/mTOR pathway and the RAS/RAF/MEK/ERK (MAPK) pathway. These act as shared signaling nodes for multiple receptors. Activated RTKs engage them by stimulating PI3K and RAS, while downstream signal propagation is mediated predominantly by serine/threonine kinases (AKT/mTOR and RAF/MEK/ERK). The PI3K/AKT/mTOR pathway regulates tumor cell growth, metabolism, survival, and angiogenesis. Its constitutive activation most commonly results from phosphatidylinositol-4,5-bisphosphate 3-kinase catalytic subunit alpha (PIK3CA) mutations, loss of phosphatase and tensin homolog (PTEN) function, or hyperactivation of upstream RTKs [22,23]. Alterations in this pathway occur across a broad spectrum of cancers (including breast, colorectal, endometrial, and lung cancers, as well as head and neck tumors) and are associated with both aggressive disease biology and resistance to other treatments, such as endocrine therapy and anti-HER2 agents [22]. These observations have supported the development of PI3K inhibitors such as **idelalisib**, **copanlisib**, and **alpelisib**, as well as mTOR inhibitors such as **everolimus** and **temsirolimus**, which are already used clinically in hematologic malignancies, hormone receptor-positive/human epidermal growth factor receptor 2-negative (HR+/HER2−) breast cancer with PIK3CA mutations, and in neuroendocrine tumors and renal cell carcinoma (RCC) [21,22]. The RAS/RAF/MEK/ERK (MAPK) pathway is the second fundamental signaling system downstream of RTKs. It comprises RAS proteins, RAF kinases (ARAF, BRAF, and RAF1/CRAF), MEK1/2, and ERK1/2. This cascade controls cell proliferation, differentiation, and survival, and aberrant activation is observed in a substantial fraction of human cancers. RAS mutations are estimated to occur in approximately 33% of solid tumors, whereas BRAF mutations are present in about 8% of cancers; the BRAF V600E mutation is particularly characteristic of melanoma and a subset of NSCLC cases [24]. Recent studies in solid tumors emphasize that, despite the efficacy of RTK-targeted drugs, deregulation of MAPK signaling, including ERK reactivation, represents one of the major mechanisms of acquired resistance to targeted therapies [25,26,27]. The clinical relevance of MAPK signaling is well illustrated by the development of BRAF and MEK inhibitors. In patients with BRAF V600-mutant melanoma, combined BRAF inhibitor (BRAFi) and MEK inhibitor (MEKi) therapy, for example, **dabrafenib** plus **trametinib**, **vemurafenib** plus **cobimetinib**, or **encorafenib** plus **binimetinib**, significantly improved objective response rates, progression-free survival, and overall survival compared with monotherapy and classical chemotherapy, as repeatedly confirmed in clinical trials and meta-analysis [28,29,30]. Similar regimens are currently used in selected cases of BRAF-mutant NSCLC and thyroid cancer, and recent reviews describe the trajectory of these drugs from organ-specific therapy (melanoma) toward tissue-agnostic use, in which treatment selection is based on genetic and molecular features rather than tumor site, in cancers harboring defined BRAF alterations [26,31]. Although the PI3K/AKT/mTOR and MAPK pathways are well-established therapeutic targets, the clinical efficacy of their inhibitors remains strongly context-dependent and is often limited by both biological and pharmacological constraints. PI3K inhibitors are frequently hindered by dose-limiting toxicities, including immune-related adverse events, metabolic disturbances, and hepatotoxicity, which restrict long-term use. Their activity is further weakened by incomplete pathway suppression and rapid adaptive resistance, particularly through crosstalk with parallel networks such as MAPK signaling. Inhibition of PI3K can relieve negative feedback and promote compensatory activation of receptor tyrosine kinases and ERK, reducing response durability. Similar limitations affect MAPK-targeted therapy: although BRAF and MEK inhibitors have shown substantial benefit, especially in BRAF V600-mutant melanoma, monotherapy often leads to rapid resistance driven mainly by ERK reactivation. The superiority of combined BRAF and MEK inhibition over single-agent treatment illustrates a central principle of targeted therapy—simultaneous blockade of multiple nodes can delay resistance and improve response durability, although at the cost of increased toxicity. Overall, the variable effectiveness of PI3K- and MAPK-directed strategies across tumor types and genetic backgrounds reflects differences in pathway dependence, co-occurring alterations, and signaling redundancy, supporting the growing shift toward biomarker-guided combination approaches rather than single-agent inhibition.

**Scheme 1:**
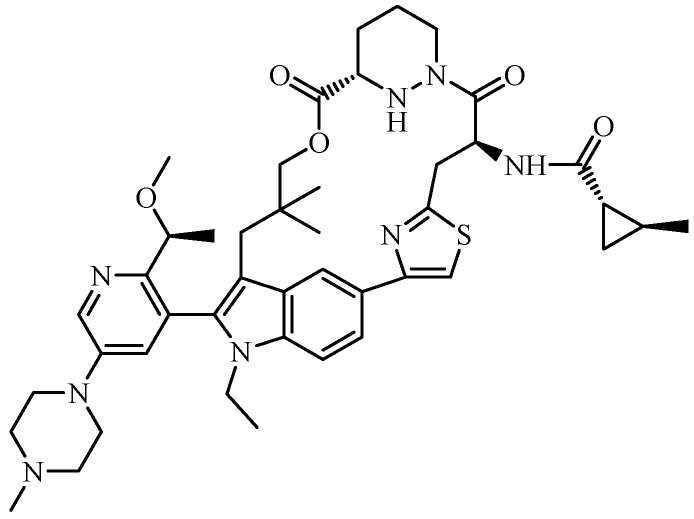
Chemical structure of **daraxonrasib** (**RMC-6236**).

**Daraxonrasib** (**RMC-6236**, Scheme 1) is an orally available, multi-selective RAS (ON) inhibitor that targets the active, guanosine triphosphate-bound form of RAS proteins, including several mutant KRAS variants, thereby suppressing signaling through the RAF-MEK-ERK pathway [32,33]. Preclinical studies demonstrated broad antitumor activity across KRAS-mutant models and reduced ERK phosphorylation, including in pancreatic and lung cancer systems [33,34]. In the first-in-human phase I study enrolling patients with advanced solid tumors harboring RAS mutations, encouraging early response rates were reported, particularly in heavily pretreated pancreatic cancer, with an acceptable safety profile dominated by gastrointestinal and skin adverse events [35,36]. These findings support direct inhibition of mutant RAS as an increasingly realistic therapeutic strategy, even in highly refractory malignancies such as pancreatic cancer; however, evidence from mouse models of pancreatic ductal adenocarcinoma (PDAC) suggests that durable responses may require simultaneous inhibition of several signaling nodes rather than monotherapy directed against a single pathway component [37]. In these models, pancreatic cancer cells maintained oncogenic potential as long as at least one of three key nodes—RAF1, EGFR, or STAT3—remained active, whereas combined suppression of all three induced rapid tumor regression and prolonged tumor-free survival in orthotopic tumors [37]. This concept was translated into a pharmacological strategy using a triple combination of a RAS inhibitor (**daraxonrasib** or the KRAS G12D-selective inhibitor MRTX1133), the EGFR family inhibitor afatinib, and the STAT3 degrader SD36, which produced complete and durable regressions in mice and showed similar efficacy in patient-derived xenograft models [37]. Collectively, these results support the rationale for combination strategies that simultaneously extinguish critical signaling nodes to achieve deep and durable responses while limiting the risk of relapse and resistance.

RTKs and their main downstream signaling cascades, PI3K/AKT/mTOR and RAS/RAF/MEK/ERK, form a convergent oncogenic signaling axis that is essential for cancer cell survival and growth. Hyperactivation of these pathways, driven by mutations, gene amplification, or gene fusions, underpins the concept of oncogene addiction and helps explain why tyrosine kinase inhibitors, PI3K/mTOR inhibitors, and BRAF/MEK inhibitors have become key pillars of contemporary targeted therapy across multiple cancer types [13,16,22,26].

### 2.2. Design of Tyrosine Kinase Inhibitors

Tyrosine kinase inhibitors (TKIs, Table 1, Table S1) are designed to bind the active site of kinases, most commonly the ATP-binding domain, thereby blocking substrate phosphorylation and, consequently, inhibiting the transmission of proliferative and anti-apoptotic signals [15]. Since the approval of the first breakthrough targeted therapy, imatinib, an inhibitor of the hyperactive BCR-ABL tyrosine kinase produced by the Philadelphia chromosome in CML, in 2001 [38], the number of clinically available TKIs has expanded to several dozen, covering multiple indications, including breast cancer and hematologic malignancies [22].

From a medicinal chemistry perspective, the diversity of tyrosine kinase inhibitors can be reduced to a limited set of recurring design principles. Most ATP-competitive inhibitors share common features, including hinge-region binding, occupation of adjacent hydrophobic pockets, and, in more advanced cases, covalent targeting of non-conserved residues. Therefore, the scaffold classes discussed below should not be viewed as independent chemical groups, but rather as alternative structural solutions to optimize these shared interactions, improve selectivity, and overcome resistance mechanisms. This perspective provides a unifying framework for interpreting the evolution of kinase inhibitor design.

TKI design relies on heterocyclic cores that fit into the ATP pocket. The choice of scaffold governs affinity, selectivity, and pharmacokinetics. Common cores include imidazopyridines, pyrimidines, quinazolines, quinolines, triazolopyrimidines, and pyrazolo[3,4-d]pyrimidines, which form the basis of many approved and investigational inhibitors [18].

Resistance to TKI monotherapy often emerges rapidly, motivating combination strategies with immunotherapies, which can prolong response and improve survival [39].

**Table 1:** Key receptor tyrosine kinases (RTKs) and non-receptor tyrosine kinases (NRTKs) in targeted therapy—molecular alterations and available inhibitors [15,40].

Receptor RTK (Gene)/Non-Receptor Kinase (Gene)	Function	Common Cancer-Associated Alterations/Indications	Representative Approved Inhibitors
EGFR	A transmembrane receptor tyrosine kinase of the ErbB family that, upon ligand binding, activates the RAS/RAF/MEK/ERK, PI3K/AKT/mTOR, and STAT pathways, thereby regulating proliferation, survival, and differentiation of epithelial cells.	Activating mutations in NSCLC; amplification and overexpression in lung cancer, including squamous cell carcinoma.	Gefitinib, erlotinib, icotinib, dacomitinib, afatinib, osimertinib
HER2	HER2 is an ErbB family receptor that primarily forms dimers with other receptors, especially HER3, thereby strongly amplifying proliferative and pro-survival signaling (MAPK, PI3K/AKT).	HER2-positive breast cancer and advanced NSCLC with HER2 exon 20 mutations.	Trastuzumab, lapatinib, neratinib, tucatinib, pyrotinib
ALK/ ROS1	Receptor tyrosine kinases that are physiologically involved in nervous system development and, in cancer, most often occur as oncogenic fusion kinases (e.g., EML4-ALK and ROS1 fusions), leading to constitutive activation of the RAS/RAF/MEK/ERK and PI3K/AKT pathways and driving tumor cell proliferation and survival.	NSCLC (ALK-positive, ROS1-positive, MET exon 14 skipping (METex14) and/or MET amplification), as well as solid tumors harboring TRK and/or ROS1 gene rearrangements.	Crizotinib, ceritinib, alectinib, brigatinib, lorlatinib, entrectinib
RET	A receptor tyrosine kinase for ligands of the glial cell line-derived neurotrophic factor (GDNF) family, which plays an important role in the development of the nervous system and thyroid C cells. Constitutive activation of this receptor (germline mutations or fusion rearrangements) drives proliferation and resistance to apoptosis, including in medullary thyroid carcinoma (MTC).	NSCLC with RET rearrangements, advanced/metastatic radioiodine-refractory thyroid cancer, advanced/metastatic MTC, and other solid tumors with RET fusions.	Selpercatinib, pralsetinib
MET (c-MET)	A receptor for hepatocyte growth factor (HGF) whose activation triggers pathways controlling proliferation, survival, migration, and invasion, including RAS/MAPK and PI3K/AKT. In cancer, overexpression, amplification, or activating alterations (e.g., MET exon 14 skipping, METex14) promote tumor growth and metastasis.	NSCLC with METex14 alterations; EGFR-mutant, MET-amplified NSCLC with acquired resistance to EGFR-TKIs (including after gefitinib and osimertinib); and pulmonary sarcomatoid carcinoma (PSC) with METex14 alterations.	Crizotinib, capmatinib, tepotinib, savolitinib
VEGFR	VEGFR1–3 are primarily expressed on endothelial cells and transduce VEGF signals into the proliferation, survival, and migration of vascular wall cells, thereby controlling angiogenesis and vascular permeability. In cancer, this enables the formation of pathological tumor vasculature.	Advanced/metastatic RCC (mRCC), unresectable or advanced hepatocellular carcinoma (HCC), differentiated thyroid carcinoma (DTC), including radioiodine-refractory disease, MTC, GIST, metastatic pancreatic neuroendocrine tumors (pNET), colorectal cancer (CRC), including metastatic CRC (mCRC), endometrial cancer, advanced NSCLC (previously treated), relapsed small cell lung cancer (SCLC; ≥3L), advanced soft tissue sarcomas (STS), and gastric/gastroesophageal junction adenocarcinoma.	Sorafenib, sunitinib, pazopanib, axitinib, lenvatinib, vandetanib, regorafenib, cabozantinib, anlotinib, apatinib, fruquintinib
PDGFR/ KIT	Receptor tyrosine kinases that regulate proliferation, differentiation, and survival of mesenchymal and hematopoietic cells; their aberrant activation (mutations or rearrangements) promotes tumor growth and angiogenesis.	Philadelphia chromosome-positive (Ph+) chronic myeloid leukemia, GIST (including KIT- and PDGFRA-mutant, unresectable or metastatic disease across different lines of therapy), and other hematologic malignancies.	Imatinib, sunitinib, regorafenib, avapritinib, ripretinib
FGFR	A family of receptors for fibroblast growth factors that integrates FGF signaling in embryonic development, angiogenesis, and cell proliferation and differentiation. In cancer, FGFR amplifications, mutations, and fusions lead to sustained activation of pro-proliferative pathways and contribute to drug resistance.	Locally advanced/metastatic urothelial carcinoma (UC) with FGFR2/3 alterations and advanced/metastatic, previously treated cholangiocarcinoma (CCA) with FGFR2 fusions/rearrangements.	Erdafitinib, pemigatinib
ABL (BCR::ABL1)	A cytoplasmic kinase; constitutively active BCR::ABL1 drives proliferation and survival of leukemic cells.	Ph+ CML (BCR::ABL1); resistance is associated, among others, with kinase domain mutations (e.g., T315I).	Imatinib, dasatinib, nilotinib, bosutinib, ponatinib, asciminib [41]
JAK (JAK1/2)	Cytoplasmic kinases in the JAK/STAT pathway; their hyperactivation drives symptoms and disease progression in myeloproliferative neoplasms.	Myelofibrosis (MF) and related myeloproliferative neoplasms (MPNs) with deregulated JAK/STAT signaling.	Ruxolitinib, fedratinib, pacritinib, momelotinib [42]
BTK (TEC family)	A cytoplasmic kinase in B-cell receptor (BCR) signaling that is essential for the proliferation and survival of malignant B lymphocytes.	B-cell lymphoproliferative malignancies (including chronic lymphocytic leukemia (CLL), mantle cell lymphoma (MCL), marginal zone lymphoma (MZL), Waldenstrom macroglobulinemia (WM), and follicular lymphoma (FL), depending on the indication).	Ibrutinib, acalabrutinib, Zanubrutinib [43]

#### 2.2.1. Imidazopyridines and Pyrimidines

**Scheme 2:**
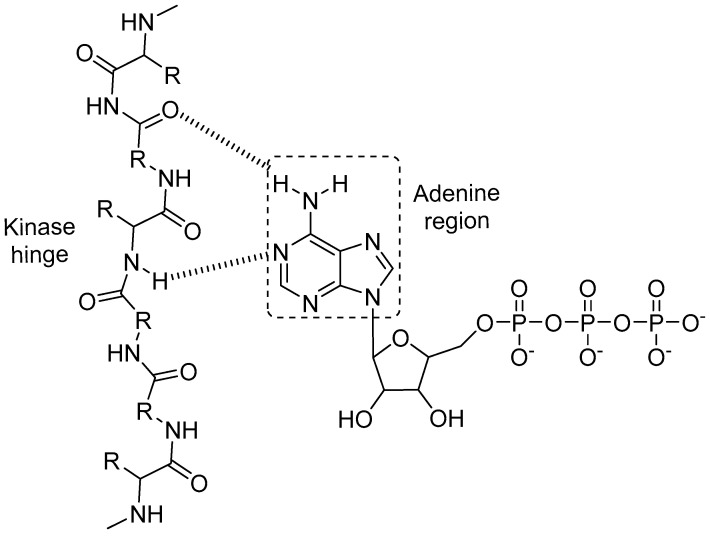
ATP interactions with the kinase hinge region [44].

Pyrimidines are privileged kinase inhibitor cores. Nitrogen positioning and amino substituents (e.g., 2-aminopyrimidine, 2,4-diaminopyrimidine) mimic the hydrogen-bonding pattern of ATP adenine at the kinase hinge, forming one or two key backbone interactions [44,45] (Scheme 2). This classical hinge-binding motif underpins broad TKI activity and informs selectivity across kinase families [44,46,47].

**Imatinib**, a 2-phenylaminopyrimidine derivative, exemplifies this approach, establishing hinge hydrogen bonds and hydrophobic interactions in BCR-ABL, KIT, and PDGFR, resulting in selective inhibition of autophosphorylation and clinically meaningful CML responses [48,49]. **Nilotinib**, a second-generation analog, preserves hinge interactions while incorporating peripheral modifications to target imatinib-resistant BCR-ABL mutants [50].

**Scheme 3:**
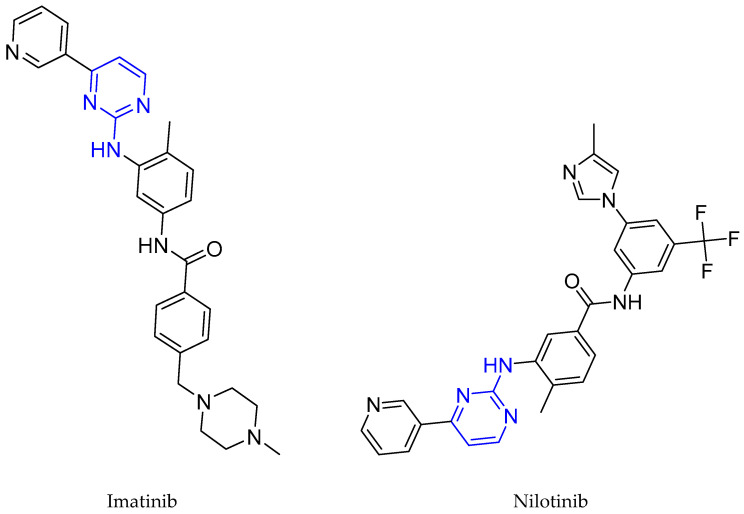

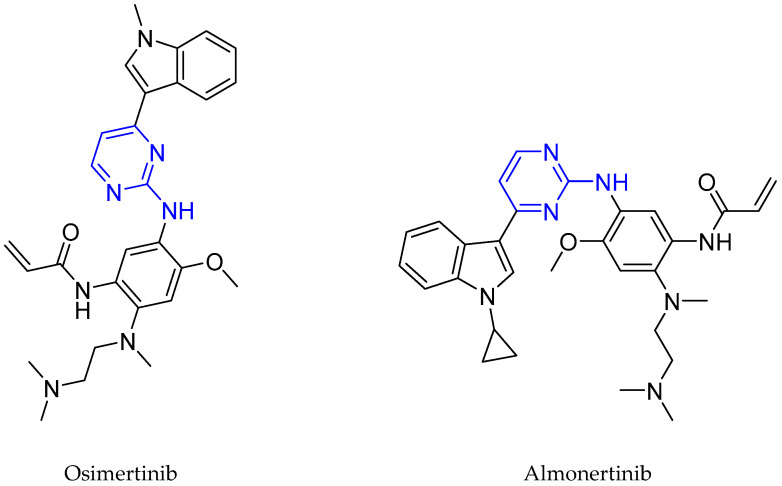
Tyrosine kinase inhibitors containing a 2-aminopyrimidine moiety. The key structural motifs discussed in the text are highlighted in blue.

The pyrimidine hinge-binding principle also informs EGFR/HER inhibitors. Third-generation agents like **osimertinib** and **almonertinib** incorporate a 2-aminopyrimidine hinge binder combined with a covalent warhead targeting Cys797 in the ATP pocket, enhancing selectivity and overcoming resistance mutations (Scheme 3) [46,51,52]. This design strategy, in which a conserved pyrimidine hinge-binding segment is combined with more diverse peripheral fragments, has enabled the development of TKIs with distinct selectivity profiles and the ability to overcome resistance driven by mutations within the kinase domain [46,51]. Importantly, the widespread use of the 2-aminopyrimidine hinge-binding motif has also contributed to cross-resistance patterns among ATP-competitive inhibitors, highlighting the need for alternative binding modes or allosteric strategies. In parallel, imidazo[1,2-a]pyridines have been explored as versatile kinase inhibitor scaffolds. Their fused, planar bicyclic system provides a rigid orientation of the ring nitrogen relative to the hinge region and allows favorable positioning of substituents toward hydrophobic pockets. The imidazo[1,2-a]pyridine core can serve as an ATP-site pharmacophore, and appropriate substitution has enabled compounds with low-nanomolar activity against PI3K and mTOR, together with strong inhibition of phosphorylation of downstream targets in cancer cell lines [53,54]. Similarly, in a series of imidazo[1,2-a]pyridine-based c-Met inhibitors, this scaffold, combined with fragments designed to occupy hydrophobic regions and the solvent-front region (the area near the entrance to the ATP pocket at the interface with the solvent), has yielded low-nanomolar c-Met inhibitors that selectively suppress c-Met phosphorylation and key downstream signaling nodes (AKT and ERK), thereby effectively limiting the proliferation of c-Met-dependent cancer cells [55]. From a drug design perspective, these findings indicate that both pyrimidines, particularly 2-aminopyrimidines, and imidazo[1,2-a]pyridines constitute complementary platforms for building tyrosine kinase inhibitors. Pyrimidines dominate among clinically used inhibitors (e.g., **imatinib**, **nilotinib**, and multiple EGFR/HER inhibitors), where they classically engage the kinase hinge region, whereas imidazo[1,2-a]pyridines offer a more compact, conformationally constrained geometry that can efficiently probe alternative regions of the ATP-binding pocket in targets such as PI3K, mTOR and c-Met, providing additional opportunities to optimize selectivity and address resistance to earlier TKI generations [40,46,52,53]. Overall, pyrimidine-based scaffolds show how a conserved hinge-binding motif can support successive generations of kinase inhibitors, whereas imidazopyridines illustrate a complementary strategy based on conformational constraint and pocket-specific selectivity tuning. Compared with classical pyrimidine-based inhibitors, imidazo[1,2-a]pyridines offer a more rigid and conformationally constrained scaffold, which may enhance selectivity by limiting conformational freedom. However, pyrimidines remain dominant in clinically approved TKIs due to their well-established hinge-binding geometry and predictable SAR. This illustrates a broader principle in kinase inhibitor design: highly optimized, “privileged” scaffolds often outperform structurally novel cores in clinical translation due to their balance of potency, selectivity, and developability.

#### 2.2.2. Quinazolines and Quinolines

Quinazolines and quinolines constitute a major class of heterocyclic scaffolds used in tyrosine kinase inhibitor design, with particular relevance for the ErbB receptor family, including EGFR (ErbB1) and HER2 (ErbB2). Both scaffold types share a planar, fused aromatic system that allows precise insertion into the ATP-binding cleft, forming hydrogen bonds with residues in the kinase hinge region, a key determinant of inhibitor affinity and selectivity [56,57,58]. In quinazolines, the most classical motif is the 4-anilinoquinazoline scaffold. The quinazoline ring serves as the hinge-binding element, whereas the anilino substituent at C-4 penetrates the hydrophobic back pocket, forming the basis of the pharmacophore for many first- and second-generation EGFR inhibitors. The ring nitrogens of the quinazoline core participate in hydrogen bonding with residues in the EGFR hinge region, while the orientation of the anilino ring and its substituents within the hydrophobic pocket is critical for affinity and selectivity [56]. Among the best clinically characterized 4-anilinoquinazolines are **gefitinib** and **erlotinib**, selective ATP-competitive EGFR inhibitors used for NSCLC with common activating EGFR kinase-domain mutations, including exon 19 deletions (del19) and L858R [52]. Structural and SAR studies show that small modifications at the C-6/C-7 positions of the quinazoline ring can significantly affect binding affinity and resistance profiles [52,59]. **Lapatinib**, also a 4-anilinoquinazoline, was designed as a dual EGFR/HER2 inhibitor; its extended aryloxy substituent increases HER2 affinity and allows clinical use in HER2-overexpressing breast cancer [56,60,61].

A further step in quinazoline-based TKI development was the introduction of covalent modification. In newer EGFR/HER2 inhibitors such as **afatinib** and **dacomitinib**, an acrylamide group was added to the quinazoline scaffold to enable covalent bond formation with a conserved cysteine residue in the kinase domain, thereby prolonging target inhibition and partially overcoming resistance to earlier generations. Importantly, these compounds retain the classical 4-anilinoquinazoline pharmacophore, whereas the acrylamide side chain is oriented toward the reactive cysteine within the active site [52,56,62].

**Scheme 4:**
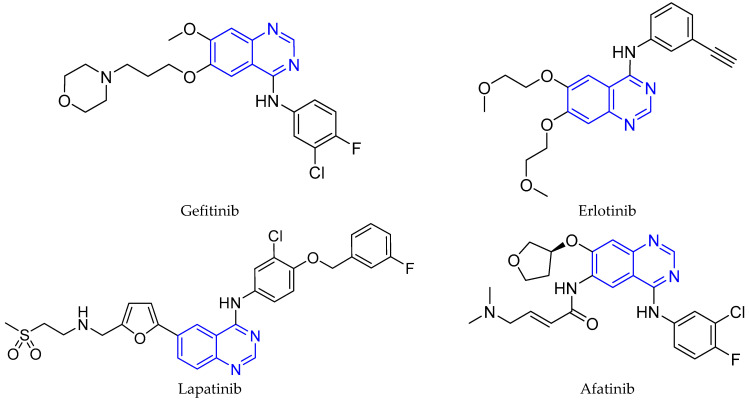

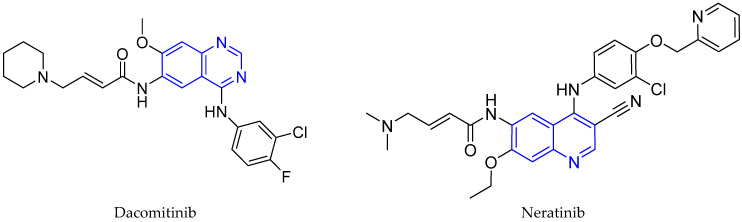
Tyrosine kinase inhibitors containing a quinazoline or quinoline moiety. The key structural motifs discussed in the text are highlighted in blue.

Quinolines are structurally related to quinazolines but contain only one nitrogen atom within the bicyclic core. This change alters the electron density distribution and basicity of the system, which affects hydrogen-bonding patterns in the hinge region and the kinase selectivity profile, while preserving the key planarity and the ability to insert into the ATP-binding pocket. The quinoline scaffold is a versatile core in numerous kinase inhibitors, including EGFR/HER2 inhibitors, VEGFR/PDGFR-targeted inhibitors, and c-Met inhibitors, and is found both in approved drugs and in compounds under investigation [63,64]. A representative example of the quinoline scaffold in ErbB-directed kinase inhibition is **neratinib**, an irreversible pan-HER inhibitor in which a 4-anilino-3-cyanoquinoline core is linked to an electrophilic substituent capable of forming a covalent bond with a conserved cysteine residue in the kinase domains of EGFR and HER2. Structural analyses indicate that the quinoline core of **neratinib** adopts a binding mode similar to that of classical 4-anilinoquinazolines within the ATP-binding pocket, thereby preserving the key interactions required for effective target engagement. At the same time, the side chain containing a Michael acceptor moiety—an electrophilic fragment designed to react irreversibly with nucleophilic residues such as the cysteine thiol—enables covalent capture of the kinase and prolongs the duration of inhibition [65]. Overall, the progression from reversible quinazoline inhibitors to covalent quinazoline and quinoline derivatives reflects an analytical design strategy that integrates scaffold geometry, hinge interactions, hydrophobic pocket occupancy, and targeted covalent chemistry. This approach facilitates fine-tuning of kinase selectivity, improves pharmacodynamic durability, and provides a structural rationale for overcoming resistance mutations such as EGFR T790M (Scheme 4) [56,66]. From a translational perspective, the evolution from reversible to covalent quinazoline inhibitors reflects a broader strategy to overcome resistance mutations such as EGFR T790M. The clinical success of quinazoline-based EGFR inhibitors highlights the importance of scaffold compatibility with both the hinge region and adjacent hydrophobic pockets. Compared with other heterocyclic cores, quinazolines provide an optimal balance between rigidity, binding affinity, and synthetic accessibility, which has contributed to their dominance in EGFR-targeted therapy.

The development of EGFR inhibitors represents one of the most illustrative examples of iterative medicinal chemistry optimization driven by resistance mechanisms. First-generation reversible inhibitors (e.g., **gefitinib**, **erlotinib**) showed efficacy in tumors harboring activating EGFR mutations but were limited by the emergence of the T790M gatekeeper mutation, which increased ATP affinity and reduced inhibitor binding. Second-generation inhibitors (e.g., **afatinib**) introduced irreversible binding through covalent interaction with Cys797, improving potency but still showing limited selectivity against T790M. This challenge was addressed by third-generation inhibitors such as **osimertinib**, which combine mutant selectivity with covalent binding and reduced activity against wild-type EGFR, thereby improving both efficacy and tolerability. More recent efforts focus on overcoming resistance mutations such as C797S and targeting atypical EGFR variants, highlighting the continuous co-evolution of drug design and tumor adaptation.

#### 2.2.3. Triazolopyrimidines and Pyrazolo[3,4-d]pyrimidines

Triazolopyrimidines and pyrazolo[3,4-d]pyrimidines are frequently used heterocyclic cores in kinase inhibitor chemistry because they are purine bioisosteres and can mimic the binding mode of the ATP adenine moiety within the catalytic cleft, forming characteristic hydrogen bonds with residues in the hinge region [67,68,69]. A review focused on JAK inhibitors highlighted that replacing the adenine-like core with fused azolopyrimidine systems, including triazolopyrimidines, can enable high selectivity and favorable pharmacokinetics while maintaining the classical ATP-competitive binding mode [70,71].

**Scheme 5:**
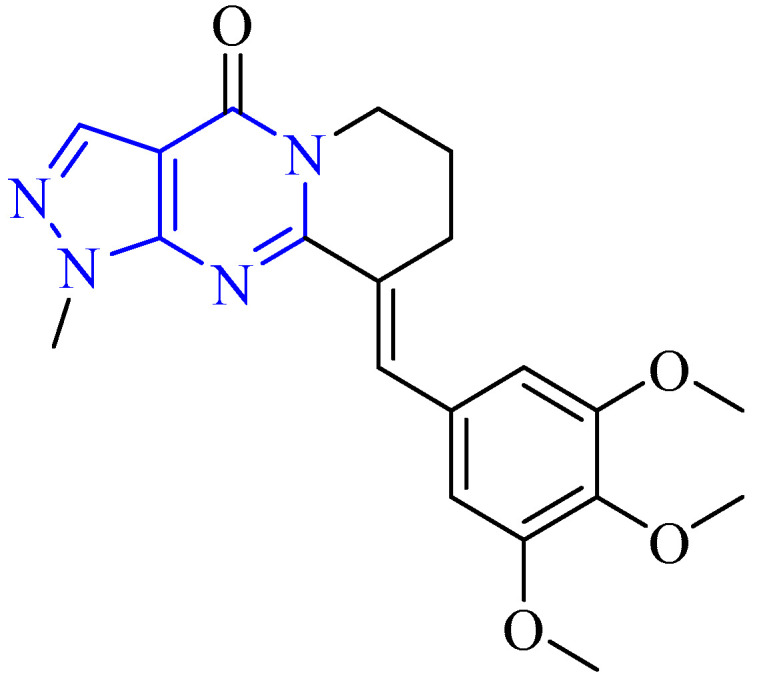
Compound **10k**. The key structural motifs discussed in the text are highlighted in blue.

**Scheme 6:**
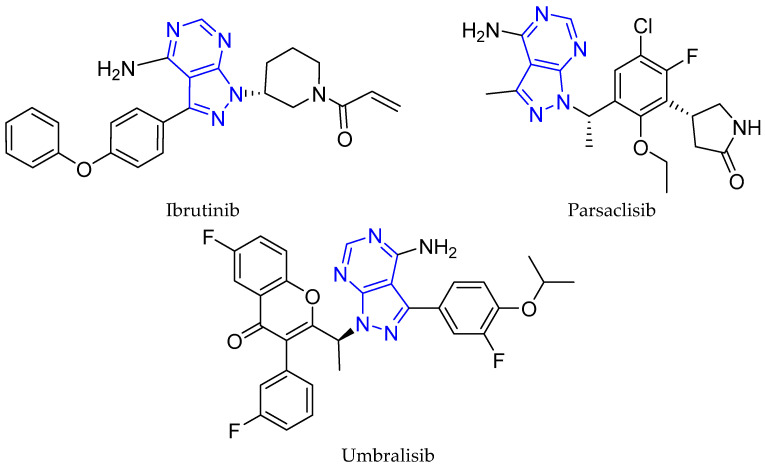
Representative drugs built on the pyrazolo[3,4-d]pyrimidine scaffold. The key structural motifs discussed in the text are highlighted in blue.

Pyrazolo[3,4-d]pyrimidines are an important class of azolopyrimidines used as kinase-inhibitor cores, particularly in compounds targeting angiogenesis-related receptor tyrosine kinases. Ruzi et al. designed tricyclic pyrazolo[3,4-d]pyrimidine derivatives as potential VEGFR-2 inhibitors and showed that appropriate substitution around the fused core enabled strong in vitro VEGFR-2 inhibition and in vivo suppression of tumor growth by the lead compound **10k** (Scheme 5). Compound **10k** interacts with the catalytic domain of VEGFR-2 through hydrogen bonding with Cys919, which contributes to effective kinase inhibition [72]. As emphasized in reviews of pyrazolo[3,4-d]pyrimidine-based kinase inhibitors (Scheme 6), this fused heterocyclic scaffold functions as an isostere of the adenine ring of ATP, allowing it to reproduce hinge-region interactions within the ATP-binding site. In addition, suitable aromatic side chains can extend into adjacent hydrophobic regions of the ATP pocket, thereby supporting both affinity optimization and selectivity tuning against related tyrosine kinases [67].

**Ibrutinib** (Imbruvica) is a 4-amino-3-(4-phenoxyphenyl)pyrazolo[3,4-d]pyrimidine derivative that contains an acrylamide moiety linked to a piperidine ring. It inhibits the proliferation and survival of B cells through irreversible binding to Bruton tyrosine kinase (BTK), thereby disrupting B-cell receptor signaling, which is frequently pathologically hyperactivated in B-cell malignancies. The drug is approved for the treatment of, among others, MCL, CLL, diffuse large B-cell lymphoma (DLBCL), WM, MZL, small lymphocytic lymphoma (SLL), and FL [73,74].

**Parsaclisib** (**INCB050465**) is a pyrazolo[3,4-d]pyrimidine derivative. Its structure contains a fused pyrazolo[3,4-d]pyrimidine core substituted with an aromatic ring and a pyrrolidin-2-one moiety. It is a next-generation selective phosphatidylinositol 3-kinase delta (PI3Kdelta) inhibitor that has been investigated mainly in B-cell lymphomas. In DLBCL, parsaclisib monotherapy (phase II CITADEL-202) showed moderate activity with an acceptable safety profile; however, the results did not support further development of monotherapy in this indication [75]. In patients with relapsed or refractory follicular lymphoma, the phase II CITADEL-213 trial reported high response rates and durable remissions, with toxicities occurring mainly in the gastrointestinal tract and mucosal tissues and generally remaining manageable [76].

**Umbralisib** is an oral small-molecule kinase inhibitor built on a pyrazolo[3,4-d]pyrimidine scaffold linked to a chromen-4-one system. It acts as a selective inhibitor of PI3Kdelta and casein kinase 1 epsilon (CK1epsilon), thereby disrupting B-cell receptor (BCR) signaling, a pathway that is central to B-cell lymphomas [77]. Based on results from the UNITY-NHL study, umbralisib received accelerated U.S. Food and Drug Administration (FDA) approval in 2021 for the treatment of adult patients with relapsed or refractory MZL after at least one prior anti-CD20-based regimen and relapsed or refractory FL after at least three prior lines of systemic therapy [77]. However, one year later, the approval was withdrawn following clinical trial data indicating a possible increased risk of death associated with its use [78,79,80].

**Scheme 7:**
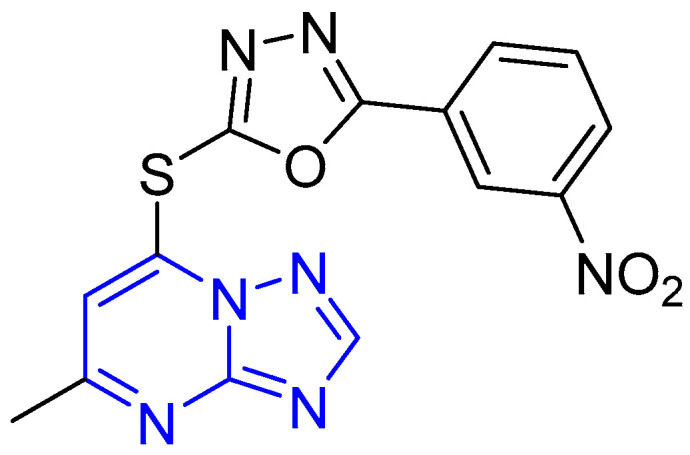
Compound **13c**. The key structural motifs discussed in the text are highlighted in blue.

Triazolopyrimidines are a privileged class of heterocycles widely explored as scaffolds for small-molecule anticancer agents directed against defined molecular targets, particularly kinases involved in tumor progression. Their value in medicinal chemistry stems from the ability of the triazolopyrimidine core to support ATP-competitive binding and preserve key hinge-region interactions across multiple oncogenic kinases [81,82,83]. For example, Eldeeb et al. developed two series of triazolo[1,5-a]pyrimidines as multi-kinase inhibitors targeting TrkA, VEGFR2, EGFR, and CDK2/CDK5, showing that the core maintains crucial hydrogen-bond interactions with hinge-region residues such as Met592 in TrkA, Asp1046 in VEGFR2, and Met769 in EGFR [83]. Similarly, Adawy et al. obtained compound **13c** from four series of triazolopyrimidine hybrids; this derivative showed very high in vitro activity against EGFR and HER2, while molecular docking confirmed binding within the catalytic site, including ATP-pocket occupancy and hinge-binding interactions (Scheme 7) [81].

Elsenbawy et al. reported thieno[2,3-d][1,2,4]triazolo[1,5-a]pyrimidines with notable cytotoxicity against MCF-7, HCT-116, and PC-3 cells. Docking studies suggested favorable binding within the catalytic sites of EGFR and PI3K, further supporting the utility of these cores in the design of tyrosine kinase inhibitors [84]. Triazole-fused pyrimidines, including triazolopyrimidine systems, represent important templates for the discovery of new targeted anticancer agents, and structure–activity relationship analysis enables rational tuning of their pharmacological properties [82].

Beyond classical RTKs, purine-like azolopyrimidine scaffolds have also been extended to non-receptor tyrosine kinases (NRTKs), including the Janus kinase family (JAK1, JAK2, JAK3, and TYK2) [85]. These cytoplasmic kinases are associated with cytokine receptors and regulate STAT-dependent transcriptional programs involved in inflammation and proliferation [86]. Because JAK catalytic domains contain a conventional ATP-binding pocket with a hinge region, adenine-mimetic cores such as pyrrolo[2,3-d]pyrimidines and related azolopyrimidines could be directly adapted for inhibitor design, supporting the development of oral ATP-competitive JAK inhibitors used clinically in inflammatory and autoimmune diseases [70,85].

**Scheme 8:**
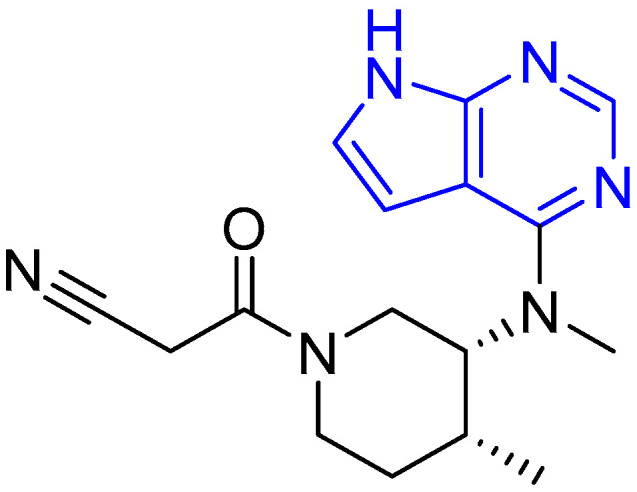
**Tofacitinib**. The key structural motifs discussed in the text are highlighted in blue.

A representative example of azolopyrimidine-based kinase inhibitor design is **tofacitinib** (**CP-690,550**) (Scheme 8), a small-molecule ATP-competitive JAK inhibitor initially developed as a selective JAK3 inhibitor and now clinically classified as a JAK1/3 inhibitor. Flanagan et al. showed that its pyrrolo[2,3-d]pyrimidine core occupies the ATP-binding site in the catalytic domain of JAK3 and forms characteristic hinge-region hydrogen bonds. Their SAR analysis further indicated that selectivity across JAK family members is influenced by the positioning of hydrogen-bond donors and acceptors as well as the size of substituents attached to the heterocyclic scaffold. A review by Clark et al. further showed that the development of JAK inhibitors has widely exploited small, purine-like pyrimidine scaffolds, including azolopyrimidine systems, and that minor changes in substituents on this core translate into substantial differences in isoform selectivity, absorption, distribution, metabolism, and excretion (ADME) properties, and safety profiles [70].

In the design of new TKIs for oncology, including immuno-oncology, this implies that fused azolopyrimidine systems, such as triazolopyrimidines and pyrazolo[3,4-d]pyrimidines, represent versatile structural platforms. They can be adapted to different kinase profiles through a combination of core cyclocondensation and late-stage SNAr modifications, and subsequently applied in rational strategies to overcome and delay resistance to targeted therapy [70,72]. Moreover, the relevance of triazolopyrimidines as potential tyrosine kinase inhibitors aligns with a broader therapeutic trend in which inhibition of receptors such as EGFR or VEGFR2, receptor tyrosine kinases that are frequently overexpressed in many solid tumors, constitutes a key component of modern targeted therapies [83].

#### 2.2.4. PI3K Inhibitors (Morpholine-Containing Scaffolds)

The PI3K/AKT/mTOR pathway is a central regulator of proliferation and survival in cancer cells, with sustained activation often driven by dysregulated BCR signaling, characteristic of chronic lymphocytic leukemia (CLL) and other B-cell malignancies. Class I PI3Ks, particularly the PI3Kδ isoform, have thus become key precision targets. Approved agents such as **idelalisib**, alongside newer PI3Kα/δ inhibitors like copanlisib, exemplify selective blockade of this pathway in B-cell cancers [87]. Idelalisib, the first PI3K inhibitor introduced for CLL, also shows activity in high-risk disease (del(17p)/TP53), though its clinical use is limited by characteristic toxicities including hepatotoxicity, severe diarrhea/colitis, skin reactions, pneumonitis, and infections [87]. Structurally, **idelalisib** is a fluorinated quinazolin-4-one linked to a purine and aromatic phenyl ring, enabling selective, ATP-competitive engagement of the PI3Kδ catalytic domain via the hinge region, specificity pocket, and adjacent hydrophobic sites [88].

**Scheme 9:**
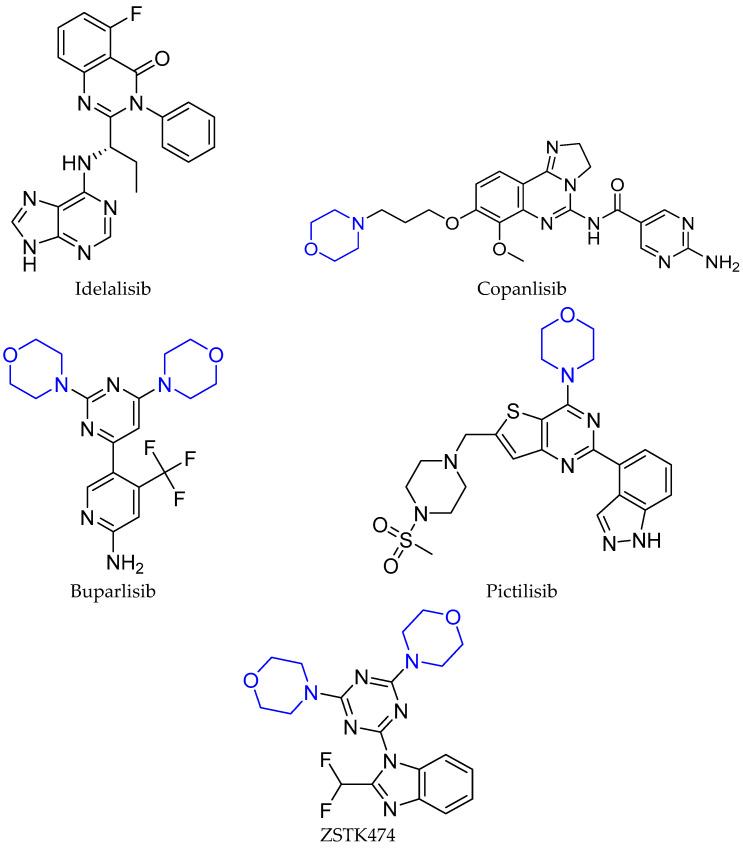
PI3K Inhibitors. The key structural motifs discussed in the text are highlighted in blue.

The morpholine ring is a privileged pharmacophore element in PI3K inhibitors, frequently positioned within the hydrophilic portion of the ATP-binding pocket to enhance hydrogen-bonding, solubility, and pharmacokinetics [89]. In most compounds, it is tethered to heteroaromatic cores such as pyrimidines or related azines, forming the defining motif of selective PI3K inhibitors (Scheme 9) [87,89].

Representative agents illustrate the versatility of this design. **Pictilisib** (**GDC-0941**), a morpholine–piperazine derivative of thieno[3,2-d]pyrimidine, is an oral pan-PI3K inhibitor with marked activity in xenograft models of ovarian cancer (IGROV1) and glioblastoma (U87MG), showing manageable toxicity in phase I clinical trials [90,91,92].

**Copanlisib** (**BAY 80-6946**) combines the morpholine motif with a tricyclic imidazo[1,2-c]quinazoline core, preferentially targeting PI3Kα/δ and receiving accelerated FDA approval for relapsed or refractory follicular lymphoma [87,93]. However, the phase III CHRONOS-4 trial evaluating the addition of copanlisib to standard treatment regimens did not meet its primary endpoint (a progression-free survival (PFS) benefit), which led to withdrawal of the approval [94].

**Buparlisib** (**NVP-BKM120**), a 2,6-dimorpholinopyrimidine, showed promising survival outcomes in combination with **paclitaxel** in HNSCC (BERIL-1), yet the subsequent phase III BURAN trial failed to demonstrate overall survival benefit [90,95,96,97].

**ZSTK474**, a triazine-based benzimidazole with two morpholine substituents, exhibits strong preclinical antitumor activity and early clinical tolerability but has not been approved [98,99].

Importantly, the clinical performance of PI3K inhibitors highlights a recurring limitation of targeted therapies: pathway inhibition often induces compensatory signaling activation, particularly within the MAPK axis, as well as upregulation of receptor tyrosine kinases [87]. This dynamic signaling plasticity represents one source of acquired resistance to PI3K inhibitors and provides a rationale for intensive development of combination strategies that pair morpholine–pyrimidine PI3K inhibitors with mTOR or MEK inhibitors, or other modulators of MAPK and the DNA damage response (DDR), to limit proliferative signaling escape. Despite a strong biological rationale, the clinical development of PI3K inhibitors has revealed significant limitations. These include a narrow therapeutic window, immune-mediated toxicities, and rapid emergence of adaptive resistance through pathway reactivation and signaling redundancy. In contrast to targets such as EGFR or BCR-ABL, where tumors often exhibit strong oncogene addiction, PI3K signaling is highly interconnected with other pathways, reducing the durability of single-agent responses. This highlights a broader challenge in targeted therapy: central signaling nodes are not always the most tractable therapeutic targets.

#### 2.2.5. Rapamycin Analogs (mTOR Inhibitors)

**Scheme 10:**
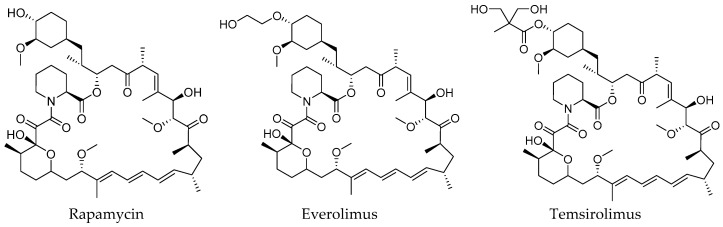
**Rapamycin** and its derivatives.

Rapamycin analogs (so-called rapalogs), such as **temsirolimus** and **everolimus**, are semisynthetic derivatives of **rapamycin** (**sirolimus**), a macrolide lactone produced by *Streptomyces hygroscopicus* (Scheme 10) [100]. Their shared structural platform enables formation of a complex with the immunophilin FKBP12 (FK506-binding protein 12), which subsequently binds to the FKBP12-rapamycin binding (FRB) domain of mechanistic target of rapamycin complex 1 (mTORC1), thereby inhibiting its catalytic activity—a mechanism that has been extensively confirmed experimentally for both rapamycin and its semisynthetic analogs [101].

**Everolimus** (**RAD001**) and **temsirolimus** (**CCI-779**) are semisynthetic derivatives of rapamycin in which the polar region of the molecule has been modified while preserving the pharmacophore responsible for binding to FKBP12 and mTORC1 [101,102]. Compared with rapamycin, these semisynthetic modifications increase bioavailability and improve pharmacokinetics. From a medicinal chemistry perspective, rapalogs exemplify a semisynthetic optimization strategy for a natural ligand: retention of the complex macrocyclic core enables precise engagement of the mTOR/FKBP12 binding interface, whereas peripheral modifications mainly improve ADME properties (e.g., solubility, clearance, and route of administration) without a substantial loss of target affinity [102].

**Temsirolimus** is a hydroxyester of **rapamycin** that was developed to increase solubility and stability [103]. In contrast, **everolimus** carries a 2-hydroxyethyl substituent at the C-40 position, which increases its oral bioavailability and enables sustained mTORC1 inhibition in solid tumors, as demonstrated in cellular and in vivo models [104]. Rapalogs primarily inhibit mTORC1, thereby relieving negative feedback constraints and promoting partial compensatory reactivation of AKT signaling—both through enhanced insulin/IGF-1 receptor input mediated by IRS-1 and through feedback-driven activation of mTORC2 that supports AKT activation. IRS-1 is a cytoplasmic adaptor protein phosphorylated downstream of the insulin receptor or insulin-like growth factor 1 receptor (IGF-1R) that propagates signals to the PI3K/AKT/mTOR pathway, and its activity is constrained by an mTORC1-dependent negative feedback loop. Relief of this feedback has repeatedly been identified as a key mechanism limiting rapalog efficacy, particularly in solid tumors [100]. From a clinical perspective, mTORC1 inhibition by rapalogs is also associated with disturbances in glucose homeostasis. Both **everolimus** and **temsirolimus** significantly increase the risk of newly diagnosed diabetes and severe hyperglycemia in oncology patients [105]. The underlying mechanisms include the development of insulin resistance secondary to attenuation of intracellular insulin signaling, as well as impaired insulin secretion due to direct effects on pancreatic beta cells, resulting in hyperglycemia that may require dedicated diabetology management. At the same time, chronic hyperglycemia and compensatory hyperinsulinemia can secondarily enhance PI3K/AKT/mTOR pathway activation in tumor cells via insulin and IGF-1 receptors, promoting proliferation, survival, and tumor progression and thereby limiting the long-term effectiveness of mTORC1 blockade [105,106].

**Temsirolimus** has demonstrated clinical efficacy, particularly in poor-prognosis RCC, as confirmed by the landmark phase III trial, which showed the superiority of **temsirolimus** over interferon α [107]. **Everolimus** has broad clinical use in the treatment of neuroendocrine tumors and advanced hormone receptor-positive/human epidermal growth factor receptor 2-negative (HR-positive/HER2-negative) breast cancer, typically in combination with endocrine therapy, as well as in pNETs. This is attributed to its sustained inhibition of mTORC1 and its ability to suppress cap-dependent protein translation through the 4E-binding protein 1 (4E-BP1)/ribosomal protein S6 kinase (S6K) axis, which comprises key mTORC1 translational effectors responsible for the synthesis of pro-proliferative proteins [102,108].

The main pharmacological limitations of rapalogs arise from compensatory PI3K/AKT activation, selective inhibition of mTORC1 only, the development of metabolic resistance, and the presence of tumor cell subpopulations with an insulin-resistant phenotype. These mechanisms have been summarized in a recent review on mTOR and PI3K inhibitors [109].

#### 2.2.6. Summary

Tyrosine kinases and the MAPK and PI3K/AKT/mTOR pathways remain core regulators of cancer cell proliferation, survival, and malignant transformation. Activating alterations in EGFR, HER2, BRAF, and PI3K, as well as ALK gene fusions, drive constitutive signaling, supporting their clinical relevance as molecular targets for kinase inhibitors. From a chemical perspective, a key role is played by a flat heteroaromatic core (e.g., pyrimidine, quinazoline, or imidazopyridine) that forms hydrogen bonds with the hinge region of the kinase domain. These pharmacophore principles underpin the activity of both classical EGFR TKIs and PI3K inhibitors featuring the characteristic morpholine–pyrimidine motif. Understanding structure–activity relationships and tumor adaptive mechanisms remains essential for the design of next-generation kinase inhibitors and optimized therapeutic regimens. Overall, modern TKI design is shifting from simple ATP-mimetic scaffolds toward structurally diverse molecules capable of addressing resistance mutations, exploiting allosteric pockets, or enabling covalent target engagement. From a translational perspective, these insights indicate that successful targeting of kinase pathways requires not only high-affinity inhibitors but also strategies that account for signaling redundancy and adaptive feedback. This further reinforces the shift toward combination-based therapeutic approaches in modern oncology. Importantly, not all kinase targets are equally “druggable” in a clinical sense. While some, such as BCR-ABL or mutant EGFR, exhibit strong oncogene addiction and sustained responses to inhibition, others, including PI3K, are embedded in highly redundant signaling networks, limiting the effectiveness of single-agent therapy. This distinction is critical for understanding why some targeted therapies achieve long-term clinical success, whereas others require combination strategies.

### 2.3. Antiapoptotic Proteins

Mitochondrial apoptosis is one of the principal mechanisms for eliminating damaged or malignant cells. Its central pillar is the BCL-2 protein family, which comprises antiapoptotic proteins (BCL-2, BCL-XL, BCL-W, MCL-1), proapoptotic effectors (BAX and BAK), and BH3-only proteins (activators: BIM, BID, PUMA, which engage BAX/BAK while antagonizing antiapoptotic proteins; and sensitizers: NOXA, BAD, BIK, HRK, BMF, which primarily neutralize antiapoptotic proteins). The balance among these subgroups determines mitochondrial outer membrane permeabilization and cytochrome c release [110,111,112]. In cancer, this balance is often shifted toward antiapoptotic proteins, enabling tumor cells to evade physiological cell death and promoting treatment resistance [113,114]. BCL-2 is a classical antiapoptotic protein that is frequently overexpressed in hematologic malignancies, including CLL, B-cell lymphomas, and a subset of acute myeloid leukemia (AML). Functional analyses in DLBCL have shown that tumor cell survival depends not only on BCL-2 but also on cooperative signaling across BCL-2, BCL-XL, and MCL-1, which is relevant for designing combination regimens and anticipating resistance [110,114]. The most clinically compelling proof of concept for therapeutically targeting BCL-2 is venetoclax, a selective oral BH3 mimetic that inhibits BCL-2 and has markedly improved rates of deep remissions in CLL and AML, confirming that direct targeting of the apoptotic machinery can be an effective anticancer strategy [115,116,117,118]. Importantly, venetoclax represented a breakthrough in relapsed/refractory CLL because it can trigger the intrinsic (mitochondrial) apoptotic cascade and retains clinical activity in TP53-altered disease, enabling responses also in patients with TP53 mutations, who have traditionally been considered a poor-prognosis group [119].

MCL-1 (myeloid cell leukemia-1) is another key antiapoptotic member of this family, with particular relevance in solid tumors and many hematologic malignancies. MCL-1 is frequently amplified and overexpressed across multiple cancers, and its upregulation is associated with adverse prognosis and resistance to multiple therapies, including BCL-2 inhibitors [112]. High tumor dependence on MCL-1 is a major determinant of cancer cell survival and stress tolerance and has also been linked to a specific dependency on fatty acid oxidation, as shown in MCL-1-dependent cancer models [120].

The recognition that BCL-2 and MCL-1 sequester proapoptotic BH3-only proteins and the BAX/BAK effectors within a specialized hydrophobic BH3-binding groove has driven the development of BH3 mimetics. These small molecules are designed to occupy this groove and displace endogenous proapoptotic proteins, thereby liberating BAX/BAK, increasing mitochondrial membrane permeabilization, and activating the caspase cascade [112,115,121,122]. **Venetoclax**, a BCL-2-selective agent, is now widely used in combination regimens (e.g., with anti-CD20 antibodies, hypomethylating agents, or kinase inhibitors), and clinical reviews have confirmed its transformative impact on outcomes, particularly in older patients or those ineligible for intensive chemotherapy [118,121,123]. However, clinical experience has also shown that selective BCL-2 inhibition can shift tumor dependence toward other antiapoptotic proteins, primarily MCL-1 and BCL-XL. Resistance analyses in patients relapsing on **venetoclax** have frequently implicated increased MCL-1 or BCL-XL expression, or altered regulation of these proteins, which diminishes treatment efficacy despite sustained BCL-2 blockade [124,125]. This phenomenon has become a major rationale for the parallel development of direct MCL-1 inhibitors and combination strategies, such as dual targeting of BCL-2 and MCL-1 or pairing BH3 mimetics with agents that reduce MCL-1 levels at the transcriptional or translational level [126]. In response, selective MCL-1 inhibitors have been intensively pursued in recent years as a promising approach to overcome resistance to conventional chemotherapeutics and to venetoclax [112,113,127]. Overall, BCL-2 and MCL-1 represent key molecular targets at the interface of tumor biology and precision therapy: overexpression of antiapoptotic proteins enables tumor cells to evade cell death, whereas BH3 mimetics, with **venetoclax** as a landmark agent and emerging MCL-1 inhibitors, provide a means to re-engage the apoptotic program.

#### 2.3.1. ABT-737 Analogs and Venetoclax

**Scheme 11:**
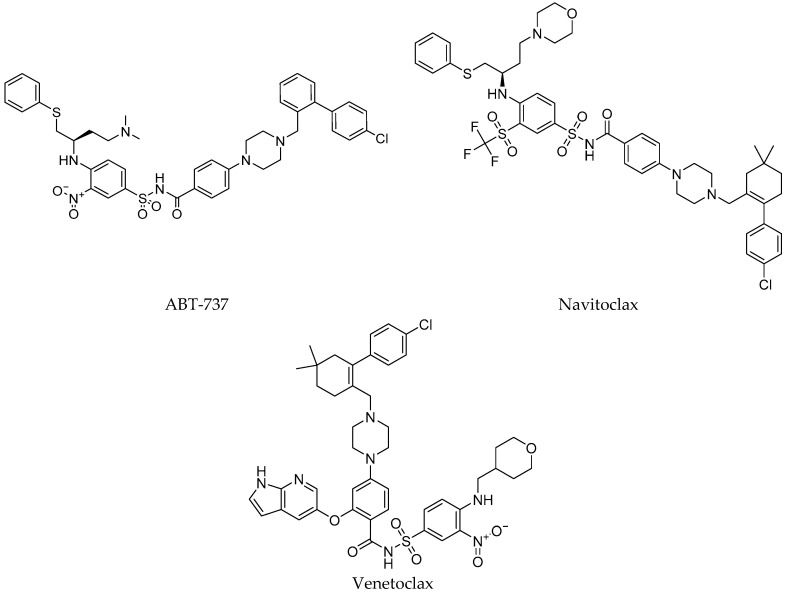
BH3-mimetic inhibitors of BCL-2.

The BCL-2 protein family includes the key antiapoptotic proteins BCL-2, BCL-XL, and MCL-1, which contain conserved BH1-BH4 domains and a C-terminal transmembrane segment that anchors them in the mitochondrial outer membrane. A shared structural hallmark of antiapoptotic members is a long hydrophobic groove formed mainly by the BH1-BH3 domains, which binds the alpha-helical BH3 motif of proapoptotic proteins (BAX/BAK and BH3-only proteins) (Scheme 11). Overexpression of BCL-2, BCL-XL, or MCL-1 in many solid and hematologic malignancies functionally neutralizes proapoptotic proteins, suppresses mitochondrial membrane permeabilization, and promotes resistance to chemo- and radiotherapy, providing the rationale for developing BH3 mimetics as small-molecule inhibitors of this family [128,129]. A landmark BH3 mimetic was ABT-737, designed to recapitulate the topology of the BH3 helix of BAD (BCL-2 associated agonist of cell death) within the binding groove of BCL-XL/BCL-2/BCL-W. **ABT-737** binds by occupying the same hydrophobic groove that is physiologically filled by the BH3 helix, with several aromatic fragments inserting into consecutive hydrophobic pockets and forming a network of hydrogen bonds with conserved residues within the BH1-BH3 region. In preclinical models, **ABT-737** showed high affinity for BCL-2, BCL-XL, and BCL-W and induced regression of solid tumors and lymphomas in mice, confirming that pharmacological engagement of the BH3 groove is sufficient to activate the intrinsic apoptotic pathway [129,130,131]. Optimization of **ABT-737** yielded the oral analog **ABT-263** (**navitoclax**), which retained a similar binding profile to BCL-2/BCL-XL but had limited clinical utility due to dose-limiting thrombocytopenia caused by BCL-XL inhibition in megakaryocytes and platelets [128,130]. Subsequent refinement of the **ABT-737**/**ABT-263** pharmacophore led to **venetoclax** (**ABT-199**), a highly selective oral BCL-2 inhibitor that preserves the BH3-mimetic binding mode while showing markedly reduced affinity for BCL-XL, translating into substantially less thrombocytopenia in preclinical studies [128,130,132]. **Venetoclax** retains the ability to occupy the BH3 groove of BCL-2 and release BAX/BAK, leading to mitochondrial outer membrane permeabilization, caspase activation, and rapid cell death. Clinically, this has translated into high activity in CLL and AML, particularly in combination regimens with hypomethylating agents or anti-CD20 antibodies on B cells [128,130]. Structurally, **ABT-737**, **navitoclax**, and **venetoclax** represent a closely related series of BH3 mimetics: a rigid, polycyclic aromatic/heteroaromatic core recapitulates the hydrophobic ridge of the BH3 helix, linker elements (including a sulfonamide fragment in the first generation) impose the geometry required to fill the groove pockets, and terminal polar groups participate in hydrogen-bonding and electrostatic interactions with conserved residues in the BH1/BH3 region [128,130,131]. Overall, the BH3 mimetic family illustrates that effective pharmacophores targeting antiapoptotic BCL-2 proteins must precisely reproduce both the topology and functional group presentation of the alpha-helical BH3 motif while accounting for differences in groove architecture among isoforms (BCL-2, BCL-XL, and MCL-1), which is critical for selectivity and the clinical safety profile of inhibitors. In contrast to kinase inhibitors, which target enzymatic activity, BH3 mimetics act by disrupting protein–protein interactions, representing a fundamentally different pharmacological strategy. This distinction is important, as it enables targeting of non-enzymatic dependencies but also requires precise structural mimicry, which makes drug design more challenging.

#### 2.3.2. MCL-1 Inhibitors

**Scheme 12:**
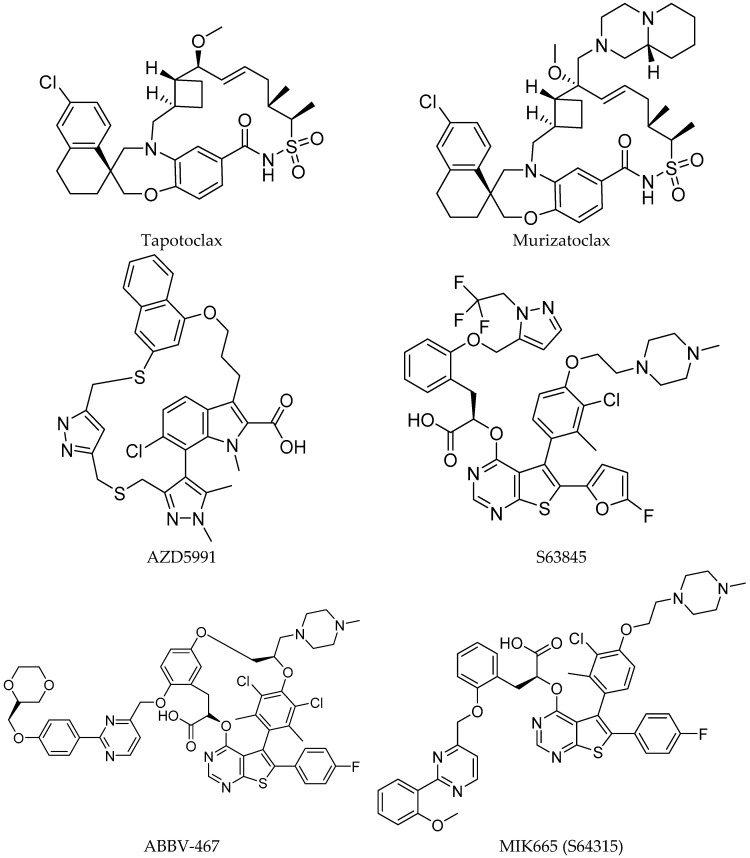
BH3-mimetic MCL-1 inhibitors.

Similarly to the multidomain architecture of BCL-2, MCL-1 adopts an eight-helix fold in which selected helices (helices 2–5 and helix 8) form a hydrophobic BH3-binding groove responsible for recognizing the BH3 region of proapoptotic BCL-2 family proteins. MCL-1 binds BH3-only activators such as BIM, BID, and PUMA and can sequester the proapoptotic effector BAK (and, depending on the cellular context, also BAX), thereby suppressing mitochondrial outer membrane permeabilization and blocking apoptosis. Under physiological conditions, genotoxic stress can induce BH3-only proteins including NOXA, which preferentially engage MCL-1 and neutralize its antiapoptotic function, promoting the release of BAK from MCL-1. This enables BAX/BAK oligomerization, mitochondrial outer membrane permeabilization, cytochrome c release into the cytosol, caspase activation, and apoptotic cell death [112,113]. In cancer cells, MCL-1 is frequently overexpressed, which helps malignant cells evade apoptosis by restraining proapoptotic signaling. Consequently, direct MCL-1 inhibitors have become one of the most actively pursued classes of BH3 mimetics [112,113]. These agents are designed to mimic BH3-helix interactions within the MCL-1 groove, which contains four hydrophobic pockets (P1-P4) and a key hotspot residue, Arg263 [133]. Recent reviews indicate that the most clinically advanced small-molecule BH3 mimetics targeting MCL-1 include **AMG-176** (**tapotoclax**) and **AMG-397** (**murizatoclax**) (Amgen), **AZD5991** (AstraZeneca), and **S63845**, together with its clinical derivative **S64315**/**MIK665** (Servier/Novartis) (Scheme 12) [112,134,135].

Crystal structures of these ligand–MCL-1 complexes reveal a recurring pharmacophore: an anionic moiety (most commonly a carboxylate or a bioisostere) forming a salt bridge or hydrogen-bonding network with Arg263, coupled with rigid, extended aromatic frameworks that occupy successive hydrophobic pockets within the BH3-binding groove [112,133,135,136].

**S64315**/**MIK665** is a selective, small-molecule MCL-1 inhibitor (a BH3 mimetic) derived from a fragment series featuring a thienopyrimidine scaffold and an anchoring carboxylic acid group [137]. In the discovery work, it was described as a clinical candidate obtained through structure-guided optimization, with a marked improvement in affinity and cellular activity compared with earlier compounds in the series [135]. S64315 acts by binding to the BH3 groove of MCL-1 and displacing proapoptotic effectors, thereby restoring intrinsic (mitochondrial) apoptosis in a BAX/BAK-dependent manner [138]. Structurally, **S64315**/**MIK665** occupies the P2 pocket and the P4-P5 region of the MCL-1 groove, which has been associated with improved binding and cellular potency [138]. Preclinical results include strong activity in hematologic cancer cells [135]. In melanoma models, MCL-1 inhibition (using compounds of the **S63845**/**S64315**/**MIK665** class) in combination with **navitoclax** produced stronger tumor cell killing and greater suppression of tumor growth than monotherapy in the described mouse xenograft model [139]. **S64315**/**MIK665** is administered intravenously and has entered clinical trials (including AML, MDS, multiple myeloma, and lymphomas), and it is also being evaluated in combination regimens, including with **venetoclax** [138].

A similar architecture, an anionic Arg263 anchor combined with a bulky, lipophilic aromatic framework tailored to the P1-P4 region, is also characteristic of **AZD5991** and other newer (often macrocyclic) MCL-1 inhibitors [134]. A prototypical compound in this class is the intravenously administered inhibitor **AMG-176**, a selective MCL-1 BH3 mimetic with negligible binding to BCL-2 and BCL-XL, which robustly induces apoptosis in MCL-1-dependent hematologic models and shows preclinical synergy with **venetoclax** [112,140]. In a phase I study in patients with relapsed or refractory multiple myeloma and AML (NCT02675452), **AMG-176** demonstrated biological activity with an acceptable safety profile; however, further development of **AMG-176**, similar to other MCL-1 inhibitors, has been constrained by emerging cardiac safety signals within this drug class [112,140,141].

Despite very promising proapoptotic activity in preclinical models and early-phase clinical trials in malignancies with strong MCL-1 dependence (multiple myeloma, AML, and selected lymphomas), none of these inhibitors has received regulatory approval to date. Development of this drug class has been constrained by cardiac safety signals, including a clinical hold on **AMG-397** and discontinuation of parts of the **AZD5991** program [112,113,141,142].

In the phase I study of **AZD5991** in patients with relapsed or refractory hematologic malignancies, asymptomatic, dose-dependent laboratory increases in troponin levels were frequently observed, without a significant impact on left ventricular ejection fraction. In contrast, in the first clinical study of **ABBV-467**, an increase in plasma troponin was reported in 4 of 8 patients, which the authors interpreted as a potential class effect of MCL-1 inhibitors [136,143].

At the same time, preclinical data and early clinical observations suggest that short, pulse-like dosing schedules and combinations with other targeted therapies (e.g., **venetoclax**, CDK9 inhibitors, azacitidine) may allow maintenance of strong apoptosis induction with an acceptable safety profile. Therefore, selective MCL-1 inhibitors remain one of the most important, although still experimental, directions in the development of proapoptotic therapies [112,113,142]. These challenges illustrate a key limitation of targeting apoptotic regulators: although biologically compelling, interference with core survival pathways may also affect normal tissues, leading to mechanism-based toxicities. As a result, the clinical success of MCL-1 inhibitors will likely depend on optimized dosing strategies and rational combination approaches rather than monotherapy.

#### 2.3.3. Summary

Antiapoptotic BCL-2 family proteins (BCL-2, BCL-XL, and MCL-1) play a central role in sustaining cancer cell survival by protecting cells from initiation of the mitochondrial apoptotic pathway. High BCL-2 expression in hematologic malignancies (particularly CLL and AML), as well as elevated MCL-1 levels in many solid tumors and in resistant multiple myeloma clones, provides a strong biological rationale for the development of BH3-mimetic inhibitors. Structurally, effective inhibitors of this family exploit the analogy to native BH3 domains by combining an aromatic core with functional groups that enable hydrogen-bonding and hydrophobic interactions within the BH3-binding groove of BCL-2 or MCL-1. Understanding structure–function relationships across the BCL-2 family and compensatory mechanisms (particularly the MCL-1-BCL-XL axis) is now critical for the rational design of combination regimens that enhance efficacy and limit resistance. Consequently, the BCL-2 family remains one of the most established and most promising targets in precision anticancer therapy, spanning both clinical development and modern medicinal chemistry.

### 2.4. DNA Repair Proteins—PARP1/2

Cancers often exhibit high replication stress and genomic instability, which can render them particularly dependent on efficient DDR pathways and create vulnerabilities that can be selectively exploited therapeutically [144]. Drugs targeting DDR components, especially poly(ADP-ribose) polymerase (PARP) inhibitors, represent a practical application of synthetic lethality by selectively eliminating tumor cells with impaired DNA repair [145]. Poly(ADP-ribose) polymerases, particularly PARP1 and PARP2, recognize a single-strand DNA break (SSB), bind to the lesion site, and catalyze PARylation of repair proteins, including themselves. As a consequence, chromatin gradually relaxes and PARP dissociates from DNA. Thus, PARP inhibitors act via two complementary mechanisms: they can prevent the dissociation of PARP and other repair factors from DNA (so-called PARP trapping) or competitively bind to the NAD+ site, thereby inhibiting PARP catalytic activity. As a result, SSBs cannot be repaired and are converted into double-strand breaks (DSBs). DSBs are repaired primarily through homologous recombination repair (HRR) or non-homologous end joining (NHEJ). HRR involves, among others, BRCA-family proteins (BRCA1/BRCA2); when these are mutated and lose their function, homologous recombination deficiency (HRD) arises. In contrast, NHEJ is a rapid but error-prone repair pathway that directly ligates broken DNA ends, frequently introducing mutations and promoting genomic instability, which underpins the concept of synthetic lethality [146].

PARP1/2 play pivotal roles in sensing and resolving DNA lesions. Upon detecting damage, they catalyze poly(ADP-ribosyl)ation (PARylation) and promote recruitment of the single-strand break repair machinery XRCC1-Polβ-Lig3 (XRCC1-DNA polymerase beta-DNA ligase III), thereby stabilizing replication forks and restoring strand integrity [147]. After binding to a DNA break, these enzymes use NAD+ as a substrate and transfer ADP-ribose units onto nuclear proteins (including histones and PARP itself), initiating poly(ADP-ribose) chain formation that facilitates assembly of downstream repair factors and modulates chromatin architecture [147]. It has been reported that the PARP1 catalytic site can be described as three functional sub-sites occupied by NAD+: the nicotinamide-binding region (NI), the phosphate-interaction region (PH), and a larger pocket accommodating the adenine moiety (AD). In practice, most PARP inhibitors mimic the nicotinamide portion of NAD+—an aromatic core bearing a carboxamide group anchors the ligand in the nicotinamide site (NI) sub-site through a conserved network of hydrogen bonds and π-π stacking, while peripheral substituents may further extend into the adjacent AD/PH regions and nearby hydrophobic areas, thereby strengthening binding affinity and shaping the pharmacological profile [148]. Heteroaromatic PARP inhibitor cores contain a carboxamide group that occupies the nicotinamide pocket within the catalytic center of PARP1/2 and forms a conserved hydrogen-bond network with residues corresponding to Gly863 and Ser904, closely mimicking the binding mode of the nicotinamide moiety of NAD+ [149]. Catalytic inhibition prevents PARP auto-modification and release from DNA, resulting in stable PARP-inhibitor-DNA complexes (PARP trapping, i.e., prolonged retention of PARP on DNA; this is a kinetic phenomenon that depends on both inhibitor binding properties and PARP exchange dynamics at the lesion site [150]) and converting single-strand breaks into toxic double-strand breaks during replication. In cells with HRD, such as those harboring BRCA1/2 mutations, these additional double-strand breaks cannot be efficiently repaired, leading to tumor-selective synthetic lethality [149].

Different PARP inhibitors can inhibit PARP1 and PARP2 to different extents, and studies have shown that, for example, niraparib and talazoparib can promote PARP2 trapping through WGR-DNA interactions (the WGR domain is a structural PARP domain that contributes to DNA binding), thereby modifying the mode of PARP2 recruitment to DNA damage sites [151]. As a result, combined catalytic inhibition and PARP trapping generate toxic DNA double-strand breaks during replication in cells with homologous recombination defects (e.g., BRCA1/2 alterations), which provides a molecular basis for the selective cytotoxicity of PARP inhibitors [147,151,152]. This biological rationale has translated into clinical benefit. In the SOLO1 trial, maintenance therapy with olaparib in patients with BRCA-mutated ovarian cancer significantly and durably prolonged PFS compared with placebo [153]. In the OlympiA trial, adjuvant olaparib improved invasive disease-free survival in patients with HER2-negative breast cancer and a germline BRCA1/2 mutation, supporting the clinical applicability of synthetic lethality beyond ovarian cancer [154]. In germline BRCA-mutated pancreatic cancer, maintenance olaparib in the POLO study significantly prolonged PFS after a response to platinum-based chemotherapy, expanding the spectrum of malignancies sensitive to PARP blockade [155]. Current research guidance also emphasizes that biomarkers beyond BRCA, namely the broadly defined HRD phenotype (impaired homologous recombination, high genomic instability, and increased sensitivity to DNA-damaging agents) and the compensatory pathways arising from it, determine responses to PARP inhibitors (PARPi) and inform the rationale for combination strategies, for example, with antiangiogenic therapy. Such combinations are pursued both to enhance efficacy and to overcome resistance driven by restoration of homologous recombination (the high-fidelity repair of DNA double-strand breaks using the sister chromatid as a template) or by stabilization of replication forks [145,152].

#### 2.4.1. Phthalazinones

**Scheme 13:**
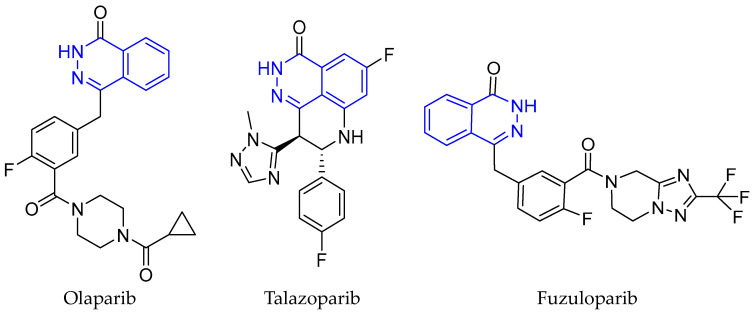
PARP inhibitors containing a phthalazinone fragment. **Olaparib** [167], **Talazoparib** [168], **Fuzuloparib** [165]. The key structural motifs discussed in the text are highlighted in blue.

Phthalazin-1(2H)-one is a well-characterized and frequently used scaffold in small-molecule drug design, including PARP inhibitors, and it represents a key structural motif in **olaparib** and **talazoparib**, as well as in numerous newer analogues investigated in preclinical studies (Scheme 13) [149,156,157]. It has been shown that 4-benzyl-substituted 2H-phthalazin-1-one derivatives bind within the catalytic site of PARP-1, mimicking the binding mode of the nicotinamide moiety of NAD+ through an analogous carboxamide arrangement and π-π interactions [158]. Further optimization of substituents at the 4-position, particularly meta-substituted benzyl aromatic groups, enabled retention of nanomolar cellular activity against PARP-1 while improving metabolic stability and pharmacokinetic properties, which directly supported the development of **AZD2281** (**olaparib**) [158,159]. The rigid heteroaromatic phthalazinone core forms key hydrogen bonds and π-π interactions within the conserved nicotinamide pocket of PARP-1/2, whereas peripheral aromatic elements and basic side chains contribute to isoform preferences, the propensity for PARP trapping on DNA, and overall ADME properties [157,160].

**Olaparib** can be regarded as an archetypal phthalazinone-based PARP inhibitor, whose structure provides high affinity for PARP1/2 and good oral bioavailability [157]. In the “Phthalazinones 2” series, Cockcroft et al. showed that meta-substituted 4-benzyl-2H-phthalazin-1-ones combine very high PARP-1 activity with a favorable profile, which served as a direct starting point for the development of olaparib [158]. For **olaparib**, the phthalazinone core provides anchoring within the NAD+ binding site, whereas its aromatic motifs occupy neighboring hydrophobic pockets in the catalytic domain of PARP1/2; meanwhile, the basic piperazine unit remains solvent-exposed and is often used as a convenient handle for further functionalization (e.g., to generate imaging analogues) [157,161].

Recent studies indicate that the **olaparib** scaffold based on a phthalazinone core can serve as a starting point for designing more complex molecules, such as dual inhibitors of PARP and HDACs, in which the moiety responsible for PARP inhibition is retained, while additional functional groups confer HDAC inhibitory activity (HDAC inhibition increases histone acetylation, promotes chromatin relaxation, and may lead to reactivation of selected genes that are silenced in cancer cells). Such compounds, beyond enhancing synthetic lethality, may promote the accumulation of DNA in the cytoplasm and activate cGAS-STING signaling—a cytosolic DNA sensor that elicits an interferon response—thereby strengthening the immunological component of anticancer activity [162].

In **talazoparib** (**BMN-673**), the phthalazinone core is fused with an additional partially hydrogenated pyridine ring, forming a tricyclic, chiral dihydropyridophthalazinone scaffold [149]. Crystallographic analyses of the catalytic domains of PARP-1 and PARP-2 in complex with BMN-673 have shown that this extended π-system establishes an extensive network of hydrogen bonds and π-π interactions within the nicotinamide pocket and its surrounding region, which translates into potent PARP-1 inhibition and exceptionally stable trapping of PARP-DNA complexes [149].

Among newer PARP inhibitors built on the phthalazin-1(2H)-one scaffold is **fuzuloparib** (**SHR-3162**), an **olaparib**-derived analogue in which the phthalazinone core is substituted at C-4 via a 4-fluorobenzyl linker and further extended with a fused triazolopyrazine-type moiety, which increases lipophilicity and supports high PARP1/2 inhibitory potency [163,164]. In preclinical models, **fuzuloparib** potently inhibits PARP-1, induces DNA double-strand breaks, triggers G2/M cell-cycle arrest and apoptosis in homologous recombination-deficient cells, and effectively sensitizes tumor cells to cytotoxic agents, while showing a favorable pharmacokinetic and toxicology profile [163]. Based on the FZOCUS-3 program, **fuzuloparib** received marketing authorization in China for the treatment of platinum-sensitive recurrent ovarian, fallopian tube, or primary peritoneal cancer in patients with a germline BRCA mutation after at least second-line chemotherapy, expanding the clinically available PARP inhibitor options [165,166].

**Scheme 14:**
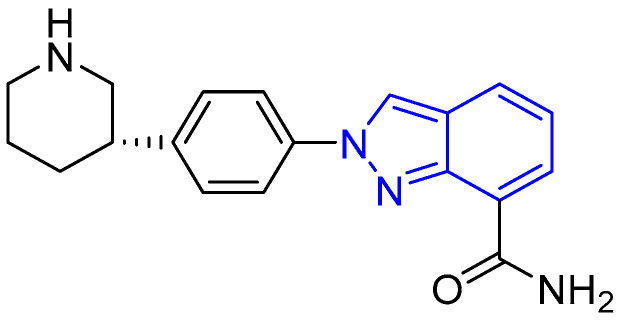
**Niraparib** [169]. The key structural motifs discussed in the text are highlighted in blue.

Unlike **olaparib** and **talazoparib**, **niraparib** is not a phthalazinone derivative but is built on a 2H-indazole-7-carboxamide scaffold, in which the indazole core is substituted at the 2-position with a 4-[(3S)-piperidin-3-yl]phenyl moiety, while retaining the key carboxamide motif that acts as a nicotinamide mimic and mediates binding within the nicotinamide pocket of PARP1/2 (Scheme 14) [169]. **Niraparib** was developed as an orally available PARP inhibitor with high activity against PARP1/2 and with clinical efficacy in tumors characterized by defects in homologous recombination; its pharmacokinetic properties have been attributed, at least in part, to physicochemical features such as lipophilicity and basicity that differ from those of more polar phthalazinone-based PARP inhibitors [169,170].

Comparative analyses of clinically used PARP inhibitors emphasize that the combination of a rigid heteroaromatic core (phthalazinone or indazole), appropriately oriented aromatic rings, and tunable basicity and polarity of amide-heterocycle tail regions constitutes a recurring structural theme that underpins high affinity for PARP1/2, PARP trapping potency, and the pharmacokinetic behavior of this class of small-molecule PARP inhibitors [157,170].

#### 2.4.2. Quinazolines

Alongside classical phthalazin-1(2H)-one cores, PARP inhibitor design increasingly explores alternative rigid heteroaromatic scaffolds, particularly 4(3H)-quinazolinones and quinoxaline frameworks, as NAD+-competitive chemotypes that can preserve the key hydrogen-bonding pattern within the nicotinamide-binding pocket of PARP-1/2 [171,172,173]. Such scaffold hopping enables retention of the characteristic interaction network in the PARP-1/2 nicotinamide site while potentially allowing tuning of isoform preference, PARP-DNA trapping potency, and ADME properties through appropriate selection of the heterocycle and peripheral aromatic substituents [171].

**Scheme 15:**
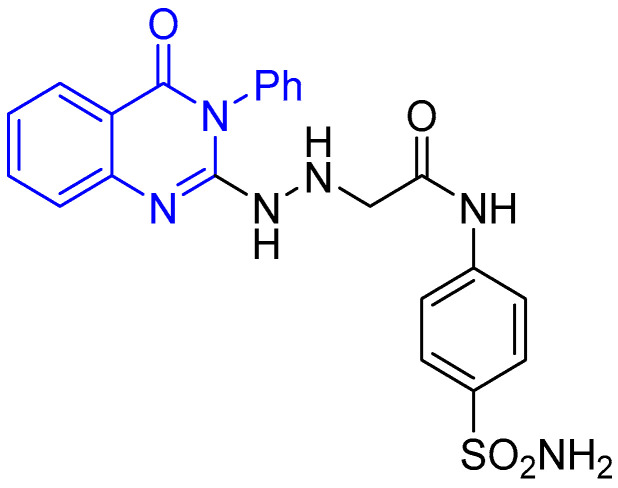
Compound **12c**, which showed the highest activity relative to olaparib [174]. The key structural motifs discussed in the text are highlighted in blue.

In the study by Ramadan et al., the 4-quinazolinone scaffold was deliberately employed as a bioisostere of the phthalazinone core of **olaparib**. The authors reported a new series of 4-quinazolinone derivatives in which most compounds exhibited substantial PARP-1 inhibitory activity, with IC_50_ values comparable to olaparib; the most active compound, **12c** (Scheme 15), also induced G2/M cell-cycle arrest in MCF-7 cells and was associated with enhanced apoptosis [174].

Molecular modeling within this series indicated that heteroatoms of the 4-quinazolinone core reproduce key nicotinamide-like hydrogen-bonding interactions in the catalytic center of PARP-1, whereas suitably substituted aromatic rings occupy adjacent hydrophobic subpockets in a binding mode comparable to the aromatic fragments of phthalazinone-based inhibitors [174].

**Scheme 16:**
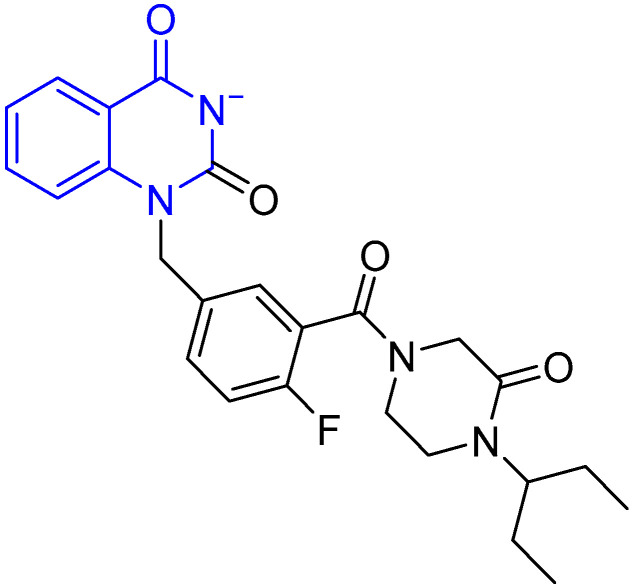
Compound **Cpd36**, which displayed the highest activity among the quinazoline-2,4-dione derivatives evaluated [175]. The key structural motifs discussed in the text are highlighted in blue.

Quinazoline-2,4-dione derivatives containing an N-substituted piperazinone motif have been identified as highly potent PARP-1/2 inhibitors with subnanomolar activity. X-ray cocrystal structures with PARP-1 and PARP-2 demonstrated a classical NAD-competitive binding mode involving both the nicotinamide-binding pocket (NI site) and the adenosine-binding region (AD site). The lead compound, **Cpd36** (Scheme 16), showed IC50 values of 0.94 nM for PARP-1 and 0.87 nM for PARP-2, with activity confirmed in cellular assays. Structural analyses indicated that the quinazoline-dione core anchors the inhibitor in the NI site, whereas the N-substituted piperazinone motif stabilizes binding through interactions within the AD site, together supporting very high potency. **Cpd36** also showed strong PARP-trapping capacity, prolonged retention on PARP-1, and dose-dependent antitumor activity in animal models, including BRCA1-deficient TNBC, PTEN-deficient prostate cancer, and colorectal cancer. Overall, this scaffold provides a rational basis for further optimization of PARP inhibitors [175].

More recent studies on quinazoline-2,4-diones confirm that an appropriate combination of electron-withdrawing substituents and basic side chains can further enhance selectivity toward PARP-1 while maintaining a favorable pharmacokinetic profile and in vitro antitumor activity [175,176].

Quinazolinones represent one of the major core scaffold classes in targeted chemotherapy, and numerous molecules featuring this framework are used clinically (mainly as tyrosine kinase inhibitors); moreover, some more recent studies have leveraged the same structural platform for the design of PARP inhibitors, which aligns with the broader trend of recycling established pharmacophores in new molecular contexts [172].

**Scheme 17:**
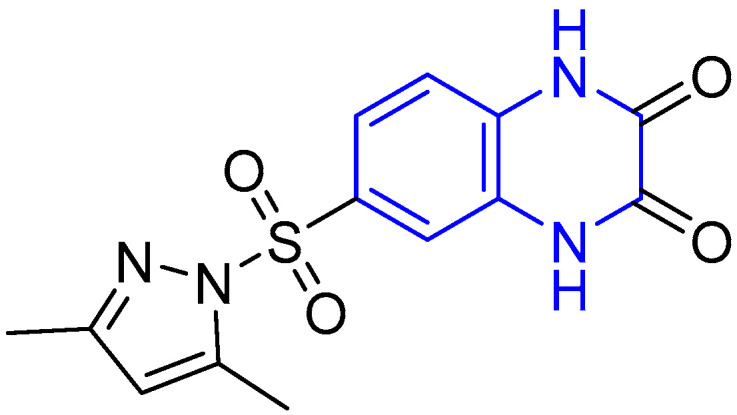
Quinoxaline-based PARP-1 inhibitor [173]. The key structural motifs discussed in the text are highlighted in blue.

Quinoxalines provide another important example of an alternative scaffold: 2,3-dioxo-1,2,3,4-tetrahydroquinoxaline was employed as a bioisostere of the phthalazinone core of **olaparib** and a new series of derivatives was synthesized (Scheme 17), in which the newly prepared compounds exhibited nanomolar PARP-1 inhibitory activity (IC_50_ range: 2.31–8.25 nM), outperforming **olaparib** (IC_50_ = 4.40 nM) in the same assay [173]. The same derivatives showed strong antitumor activity in BRCA1-mutant breast cancer cells (MDA-MB-436), and detailed cell-cycle and apoptosis analyses confirmed G2/M-phase arrest, induction of apoptosis, and enhancement of autophagy, consistent with a canonical synthetic-lethal mechanism associated with PARP-1 inhibition [173].

A range of pyridazine-based PARP-1 inhibitors has been reported, in which the diazine core serves as a scaffold that carries the pharmacophoric elements engaging the catalytic center of the enzyme, often in combination with benzamide fragments reminiscent of classical PARP inhibitors, enabling compounds with meaningful PARP-1 inhibitory activity and a favorable selectivity profile [177]. The authors emphasize that variations in the substitution pattern on the pyridazine ring, together with the introduction of additional heteroatoms within the core and peripheral fragments, markedly affect affinity for the PARP-1 catalytic site and the selectivity profile, which is critical for achieving an optimal balance between efficacy and potential toxicity of new PARP inhibitors [177].

**Scheme 18:**
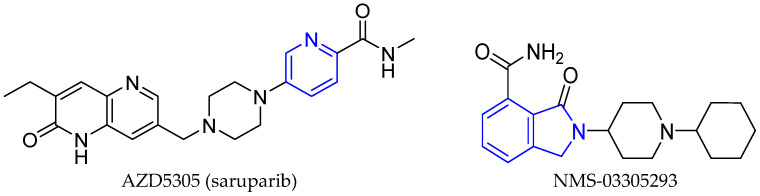
Pyridine-based and imidazopyridine-based PARP-1 inhibitors. The key structural motifs discussed in the text are highlighted in blue.

Next-generation selective PARP-1 inhibitors, such as **AZD5305** (**saruparib**) and **NMS-03305293** (Scheme 18), represent a logical extension of the ‘reduced heterocycle’ concept: the use of compact cores (including pyridine-derived and imidazopyridine-derived motifs) that preserve key hydrogen-bonding interactions within the NAD+ binding site, while limiting PARP-2 inhibition, translates in preclinical models into higher PARP-1 selectivity and potentially an improved hematologic safety profile [178,179].

Collectively, the available evidence indicates that quinazolinones, quinazoline-2,4-diones, pyridazines, and quinoxalines constitute a mature and phthalazinone-complementary family of heterocyclic cores for PARP inhibitors; selecting a given scaffold, aided by modern cross-coupling approaches and late-stage amide functionalization, enables a rational balance between PARP-1/2 affinity, PARP-DNA trapping capacity, isoform selectivity, and ADME parameters across this drug class [171,172,173,174,175,177].

#### 2.4.3. Other PARP Inhibitors

**Scheme 19:**
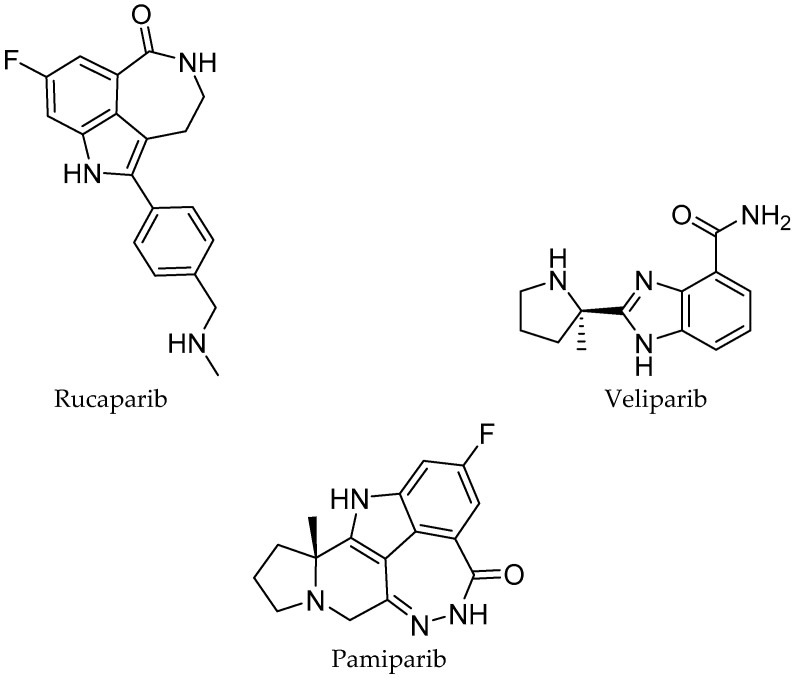
Other NAD+-competitive PARP inhibitors.

The remaining clinically used PARP inhibitors, **rucaparib**, **veliparib**, and **pamiparib** (Scheme 19), represent distinct chemotypes; however, they all share a common NAD+-competitive pharmacophore, typically comprising a carboxamide fragment conjugated to a heteroaromatic core that recapitulates the key hydrogen-bonding pattern of nicotinamide within the catalytic site of PARP-1/2 [157]. **Rucaparib** is a small molecule built on a tricyclic scaffold that mimics the nicotinamide pharmacophore, enabling tight accommodation within the catalytic pocket of PARP-1/2/3 and resulting in potent multi-enzyme inhibition in BRCA-mutant cellular models [180,181,182]. Clinically, rucaparib has been used, among others, in BRCA-mutated ovarian cancer, and its antitumor activity relies on both catalytic PARP inhibition and synthetic lethality driven by the accumulation of DNA damage in homologous recombination (HR)-deficient cells [183]. **Pamiparib** (**BGB-290**) is a fluorinated polycyclic dihydrodiazepinoindolone derivative containing four nitrogen atoms within the ring system. It is a potent and selective PARP-1/2 inhibitor with high affinity and blood–brain barrier penetration, making it particularly attractive for central nervous system (CNS) tumors [184]. Preclinical studies and early clinical trials indicate that **pamiparib** combines strong catalytic inhibition with efficient PARP trapping and a favorable brain pharmacokinetic profile, translating into promising antitumor activity in glioma and other brain tumor models and providing a rationale for ongoing combination studies with radiotherapy and chemotherapy [184,185]. **Veliparib** (**ABT-888**) belongs to the class of small benzimidazole carboxamides and was designed as a potent, orally available PARP-1/2 inhibitor with good tissue distribution [186,187]. Comparative studies of PARP trapping by clinical PARP inhibitors have shown that **veliparib** stabilizes PARP-DNA complexes relatively weakly compared with **olaparib** or **talazoparib**, which is associated with lower intrinsic cytotoxicity but supports its use in combinations with chemotherapy and radiotherapy with an acceptable safety profile [188].

#### 2.4.4. Summary

Poly(ADP-ribose) polymerases (PARP1/2) are key enzymes that initiate the repair of DNA single-strand breaks, and their pharmacological inhibition leads to the accumulation of replication-associated lesions and synthetic lethality in cells lacking efficient homologous recombination (HR), particularly those harboring BRCA1/2 mutations. The pharmacophore basis of PARP inhibitor efficacy relies on heteroaromatic cores that accurately reproduce NAD+ recognition within the PARP catalytic site. These agents must not only inhibit enzymatic activity but also stabilize the PARP-DNA complex (PARP trapping), which represents a major driver of cytotoxicity in HR-deficient tumors.

The development of next-generation inhibitors has focused on structural modifications of the phthalazinone framework and the introduction of alternative heterocyclic cores (e.g., quinazolines and triazines), enabling fine-tuning of inhibitory potency and selected pharmacokinetic properties.

Resistance to PARP inhibitors remains an active area of research and includes, among others, reversion mutations that restore HR, replication-fork stabilization, and PARP alterations that reduce trapping. Understanding these mechanisms is currently central to the design of subsequent inhibitor generations and rational combination strategies that may overcome tolerance to DNA damage and expand clinical indications.

### 2.5. Epigenetic Regulators

Epigenetic dysregulation, particularly alterations affecting histone acetylation and chromatin organization, plays an important role in oncogenesis and has become a clinically relevant target for precision therapies in cancer [189]. Histone deacetylases (HDACs) remove acetyl groups from lysine residues of histones and non-histone proteins, thereby promoting chromatin condensation, repression of tumor-suppressor gene expression, and cancer cell survival; pharmacological HDAC inhibition can restore the expression of growth-restricting genes and trigger cell-cycle arrest and apoptosis [190]. Hydroxamate-based HDAC inhibitors (HDACi), including vorinostat and belinostat, were among the first clinically developed agents in this class and typically target class I/II HDACs, increasing acetylation of histones and key regulatory proteins, which translates into antiproliferative and proapoptotic effects across multiple cancer models [189]. In parallel, non-hydroxamate HDACi have been intensively explored to improve isoform selectivity and safety; examples include o-aminobenzamides that primarily target class I HDACs, as well as other chemotypes evaluated for reduced toxicity and prolonged activity relative to hydroxamates [191]. In addition, non-hydroxamate HDAC6-selective inhibitors (e.g., benzimidazole-linked (thio)barbiturate derivatives) have been reported to achieve nanomolar potency, supporting the feasibility of effective isoform targeting without a hydroxamate group [192]. From a therapeutic perspective, combining HDACi with other targeted therapies or immunotherapy may provide additional clinical benefits, but it requires careful toxicity monitoring and further biomarker development to optimize patient selection for such regimens [190].

Bromodomain and extra-terminal (BET) family proteins (BRD2/3/4), which contain bromodomains that recognize acetylated lysines on histones, function as epigenetic readers and support transcription of oncogenic programs (including MYC-driven networks), making them attractive therapeutic targets [193]. BET inhibitors (e.g., the chemical probe **JQ1** and clinical derivatives such as **OTX015**/**MK-8628**) block bromodomain binding to acetylated histones, thereby downregulating pro-proliferative transcriptional outputs and potentially inducing cell-cycle arrest and apoptosis in models of leukemia and solid tumors [194]. Translational studies indicate that pharmacological inhibition of BRD4/BET reshapes transcriptional networks essential for maintenance of the malignant phenotype and may enhance sensitivity to combination strategies [195]. Early clinical studies of **OTX015** in hematologic malignancies demonstrated biological activity and feasibility of the approach, although further development requires optimization of dosing, combination regimens, and patient-selection criteria due to toxicity and limited efficacy as monotherapy [196].

#### 2.5.1. Hydroxamate HDAC Inhibitors

**Scheme 20:**
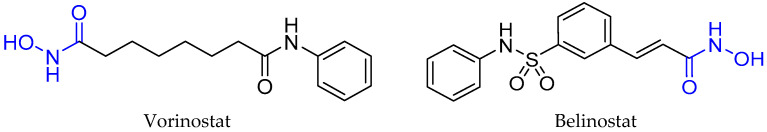
HDAC inhibitors with a hydroxamide structure. **Vorinostat** [198], **Belinostat** [210]. The key structural motifs discussed in the text are highlighted in blue.

Hydroxamate-structured HDAC inhibitors, **vorinostat** and **belinostat** (Scheme 20), belong to the classical tri-partite ligands of zinc-dependent histone deacetylases, comprising an aromatic cap group, a hydrophobic aliphatic linker, and a terminal hydroxamate moiety that acts as a bidentate Zn^2+^ chelator within the catalytic center of the enzyme [197]. Structural analyses have shown that this arrangement, i.e., an aromatic fragment interacting with the rim/surface of the HDAC pocket, a carbon linker, and a terminal -CONHOH group, represents a shared motif for **vorinostat** (**SAHA**) and **belinostat**, as well as for multiple newer hydroxamate analogues [197]. Analysis of SAHA-like series indicates that optimal HDAC inhibitory activity is typically achieved with linkers containing five to six carbon atoms, whereas both shortening and excessive elongation tend to reduce enzyme-ligand affinity [197]. **Vorinostat** (suberoylanilide hydroxamic acid, **SAHA**) was approved in 2006 by the FDA for the treatment of cutaneous manifestations of CTCL (cutaneous T-cell lymphoma) in patients with progressive, persistent, or recurrent disease after at least two systemic therapies. This decision was supported by an open-label Phase II clinical trial enrolling 74 patients, predominantly with stage IIB disease or higher, including 41% with Sezary syndrome [198]. In Phase II studies in advanced, heavily pretreated CTCL, vorinostat produced an objective response (almost exclusively partial) in approximately 25–30% of patients, with a median time to progression of approximately 6–7 months [198]. **Vorinostat** is considered a prototypical linear pan-HDAC inhibitor, in which a p-hydroxyanilide ring is connected to the hydroxamate group via a simple aliphatic chain, enabling simultaneous positioning of the aromatic cap at the entrance of the catalytic tunnel and efficient Zn^2+^ coordination by the -CONHOH group at its base [197]. Detailed structure–activity relationship analyses indicate that lengthening or shortening this linker impairs HDAC affinity, whereas modifications within the aromatic fragment (phenyl-ring substitution and the introduction of heteroaromatics) can be used to modulate selectivity toward individual class I/II HDAC isoforms [197].

In 2025, **vorinostat** remains under active clinical investigation, and current research directions also include indications extending beyond its classical hematologic use. Studies have explored **vorinostat** in uveal melanoma, where it is assessed with respect to pathways regulating tumor-cell growth and potential response biomarkers; a Phase II study conducted by the National Cancer Institute (NCT01587352) is discussed in recent reviews as ongoing or having completed accrual, with results still awaited [199,200]. Of particular interest is the VALOR program, presented at the American Society of Clinical Oncology (ASCO) 2025 as a trial-in-progress abstract, in which vorinostat is evaluated as an epigenetic modulator of prostate-specific membrane antigen (PSMA) expression in patients with PSMA-low metastatic castration-resistant prostate cancer (mCRPC). The study hypothesis is that epigenetic priming with **vorinostat** may increase PSMA expression and improve uptake of ^177^Lu-PSMA-617, potentially expanding PSMA-targeted radioligand therapy to a patient population previously considered less amenable to this approach [201].

**Belinostat** (**PXD101**) is an HDAC inhibitor with a sulfonamide-hydroxamate chemotype that retains the hallmark pharmacophore of this class, comprising an aromatic cap group, an aliphatic linker, and a terminal hydroxamate moiety. Compared with simpler analogues, **belinostat** features a more extended aryl-sulfonamide fragment, which may increase the contribution of π-π interactions and hydrogen-bonding contacts with amino-acid side chains lining the HDAC catalytic tunnel. In 2014, the FDA approved **belinostat** for the treatment of patients with relapsed or refractory peripheral T-cell lymphoma (PTCL) [202].

The most recent studies on **belinostat** are shifting it from a classical pan-HDAC inhibitor in PTCL/CTCL toward a drug evaluated in complex combination regimens, with the aim of achieving synergistic activity of both agents. In a Phase I study, Maher et al. assessed **belinostat** in combination with the NEDD8-activating enzyme inhibitor **pevonedistat** (**MLN4924**) in patients with relapsed/refractory AML or high-risk myelodysplastic syndrome; the regimen proved feasible and safe (no DLT—dose-limiting toxicity—was observed, and hematologic adverse events predominated), one patient achieved a complete remission and several had stable disease, and pharmacodynamic studies showed increased expression of markers related to oxidative stress, glutathione metabolism, and ferroptosis-associated pathways, suggesting the involvement of these processes in the response to the **pevonedistat-belinostat** combination [203]. Thomine et al. showed that in ovarian cancer SKOV3 and IGROV1-R10 cells, **belinostat** exerts cytostatic effects, whereas at higher concentrations it induces apoptosis, driving a concentration-dependent increase in the levels of the pro-apoptotic proteins BIM, PUMA, and NOXA, together with a partial reduction in Bcl-XL expression; pharmacological inhibition of Bcl-XL or Mcl-1 strongly sensitized both cell lines and patient-derived tumor organoids (PDTO) to **belinostat**, positioning the belinostat plus a Bcl-XL/Mcl-1 inhibitor regimen as a promising strategy in ovarian cancer. At the same time, the authors emphasize that the clinical use of BH3 mimetics targeting Bcl-XL and Mcl-1 is currently limited by on-target toxicity (adverse effects resulting from inhibition of the physiological function of the intact target protein, e.g., thrombocytopenia upon Bcl-XL blockade or cardiotoxicity upon Mcl-1 inhibition), i.e., the same mechanism that underlies the antitumor effect, and therefore combining these agents with **belinostat** may in the future allow dose reduction to a better tolerated range while maintaining antitumor efficacy [204].

In comparative studies of first-generation inhibitors, **belinostat** and **vorinostat** are classified as non-selective (pan-HDAC) hydroxamates with a similar mode of binding to the Zn^2+^ center; however, they differ in their pharmacokinetic profiles and the scope of antitumor activity, which provided a starting point for further optimization of hydroxamate HDAC inhibitors [197]. Examples include coumarin-based hydroxamate derivatives, which exhibit strong HDAC inhibitory activity (particularly against HDAC1) and pronounced cytotoxicity in cancer cell lines, suggesting their potential development as anticancer agents [205,206]. Another direction involves biphenyl hydroxamates designed as dual PD-L1/class I HDAC inhibitors, integrating epigenetic activity with modulation of immune checkpoints [207]. In parallel, N-hydroxycinnamamide derivatives are being developed; in THP-1 leukemia cells, these compounds suppress proliferation and induce apoptosis and cell-cycle arrest [208]. Collectively, these studies indicate that hydroxamate HDAC inhibitors are no longer viewed exclusively as non-selective pan-inhibitors, but rather as a platform for creating highly targeted, multifunctional molecules that can combine epigenetic modulation with immunotherapy or isoform-directed activity [209].

**Scheme 21:**
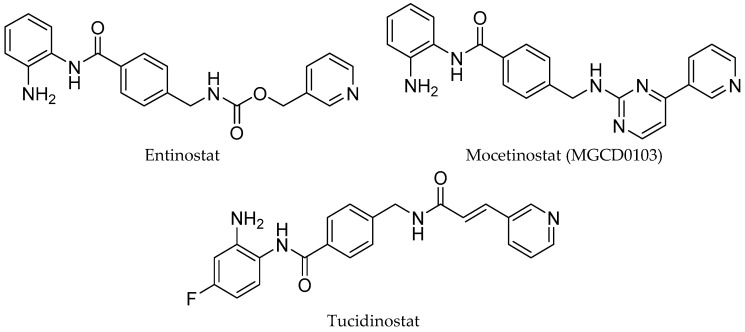
Benzamide HDAC inhibitors.

Non-hydroxamate HDAC inhibitors primarily include benzamide derivatives as well as other non-hydroxamate chemotypes, which are being developed, among others, to reduce non-selective metal binding and the toxicity associated with broadly chelating hydroxamates [211,212]. Compared with hydroxamates, benzamides (so-called ortho-aminoanilides) display slower yet highly selective inhibition of class I HDACs (HDAC1/2/3), translating into a more favorable pharmacodynamic profile and enabling long-term oral dosing. Among the best clinically characterized benzamide HDAC inhibitors are **entinostat** (**MS-275**), **mocetinostat** (**MGCD0103**), and **tucidinostat** (chidamide) (Scheme 21), all of which are based on the ortho-aminobenzamide motif as the Zn^2+^-binding group [213,214,215,216,217,218].

**Entinostat** is an oral, class I-selective HDAC inhibitor (HDAC1/2/3) in which the 2-aminobenzamide pharmacophore coordinates Zn^2+^, while a substituted pyridine ring serves as the cap group, supporting stable binding within the catalytic site [213,214,217]. Reviews on entinostat emphasize that it is a representative class I inhibitor with a favorable pharmacokinetic profile, evaluated in multiple Phase I/II studies and in the large Phase III E2112 trial in combination with exemestane in patients with advanced HR+/HER2- breast cancer, although it has not yet received regulatory approval [217,219]. **Tucidinostat** (chidamide) is an oral, subtype-selective benzamide HDAC inhibitor targeting HDAC1, HDAC2, HDAC3, and HDAC10, approved in China, among others, for relapsed/refractory T-cell lymphomas and for HR+/HER2- breast cancer in combination with exemestane [213,215,216,220]. Real-world evidence suggests that tucidinostat may provide clinical benefit with an acceptable safety profile in patients with advanced HR+/HER2- breast cancer, supporting the clinical relevance of benzamide HDAC inhibitors in this setting [216,221].

Recent studies on N-(2-aminophenyl)benzamides show that appropriate optimization of the cap group and linker enables the development of potent and selective HDAC1/2 inhibitors with antiproliferative activity in cellular models, supporting the view that the 2-aminobenzamide motif remains a versatile platform for the design of non-hydroxamate HDAC inhibitors [215].

Thiazolidinediones represent a distinct class of non-hydroxamate HDAC inhibitors, in which the 2,4-thiazolidinedione ring serves as an alternative Zn^2+^-binding group while retaining the canonical HDAC inhibitor architecture, i.e., an aromatic cap group, a flexible linker, and a terminal metal-interacting moiety [212,222]. Studies on N-substituted thiazolidinediones demonstrated that appropriate optimization of the aromatic cap group and linker can yield highly active HDAC8 inhibitors, and molecular modeling supports the placement of the hydrophobic cap at the pocket entrance and anchoring of the thiazolidinedione ring in the vicinity of Zn^2+^ [222].

A distinct category of non-hydroxamate HDAC inhibitors comprises cyclic peptides and depsipeptides, such as romidepsin (Istodax), in which Zn^2+^ coordination is mediated by a free thiol generated upon prodrug biotransformation; this agent is approved for the treatment of CTCL and PTCL, demonstrating that effective HDAC inhibition can also be achieved without a classical hydroxamate group [223,224].

Recent studies on HDAC11 indicate that highly selective, non-hydroxamate inhibitors can be designed in which Zn^2+^ coordination is mediated by alternative motifs (e.g., a carbonyl oxygen donor or a heteroaromatic ring nitrogen), thereby bypassing the canonical -CONHOH warhead while still achieving micromolar potency against HDAC11 and a highly promising selectivity profile, supporting the feasibility of moving beyond hydroxamates as a universal metal-binding group [212]. Collectively, these data suggest that non-hydroxamate HDAC inhibitors constitute an expanding family of compounds aimed at improved isoenzyme selectivity, more favorable pharmacokinetic properties, and potentially reduced toxicity risk associated with non-selective metal binding by hydroxamates, while retaining epigenetic modulatory activity [211,212,225].

#### 2.5.2. BET Bromodomain Inhibitors

BET bromodomains (BRD2, BRD3, BRD4, and BRDT) act as readers of histone N-acetyllysine marks, and pharmacological blockade of the acetyllysine-binding pocket disrupts the recruitment of transcriptional complexes to promoters and enhancers of genes critical for tumor cell survival, such as MYC [226,227].

BRD2, which is predominantly localized in the nucleus, has been described as an atypical protein kinase and supports the activation of the transcription factors E2F1 and E2F2, key regulators of gene expression controlling the G1-to-S transition [195,228]. BRD2 binds acetylated histones H3 and H4 and, by recruiting transcription factors, coactivators, and repressors (including, among others, histone deacetylases), modulates transcriptional activity and chromatin remodeling [195].

BRD3 supports the G1-to-S transition by regulating cyclin D1 transcription, a key cell-cycle protein that promotes entry into the DNA synthesis phase [195]. BRD4 is a well-studied nuclear BET family member that regulates the cell cycle, embryonic development, and genome stability through its association with chromosomes during mitosis [195,229]. A key function of BRD4 is the recruitment and activation of the P-TEFb complex, which enhances CDK9-dependent phosphorylation of the RNA polymerase II (RNA Pol II) C-terminal domain and enables productive transcriptional elongation of genes linked to cellular proliferation [230]. BRD4 also contributes to chromatin remodeling and the DNA damage response, including processes related to NHEJ, thereby supporting genome stability and promoting tumor cell survival [195,231,232,233]. A major regulatory output of BRD4 is the promotion of c-MYC transcription [234]. BRDT is a testis-specific bromodomain protein whose loss results in infertility with reduced sperm count and abnormal sperm morphology, as it regulates cyclin A1 expression, which is required for germ cells to enter the first meiotic division [235].

Inhibitors targeting these proteins may suppress oncogene expression driven by BET-dependent transcriptional programs, thereby acting as potential anticancer agents. Structural and functional studies on BET inhibitors indicate that small molecules occupy the hydrophobic KAc-binding pocket (lysine acetylation—the acetyl-lysine recognition site within the bromodomain) in the BD1/BD2 domains and competitively displace acetylated histone tails, which results in transcriptional reprogramming of cancer cells, including attenuation of MYC-dependent superenhancer activity and reduced expression of cell-cycle and anti-apoptotic genes [195]. From a chemical standpoint, common features of BET inhibitors include a planar heteroaromatic core that fills the KAc pocket, substituents that tune physicochemical properties and selectivity, and the feasibility of late-stage functionalization (e.g., amidation, N-alkylation, and cross-coupling reactions), enabling rapid generation of analogue libraries while preserving the key pharmacophore [194,195].

In melanoma models, a BET inhibitor was shown to suppress tumor progression by modulating the noncanonical NF-κB/SPP1 axis, which was associated with attenuation of transcriptional programs that support cancer cell growth and survival [236].

**Scheme 22:**
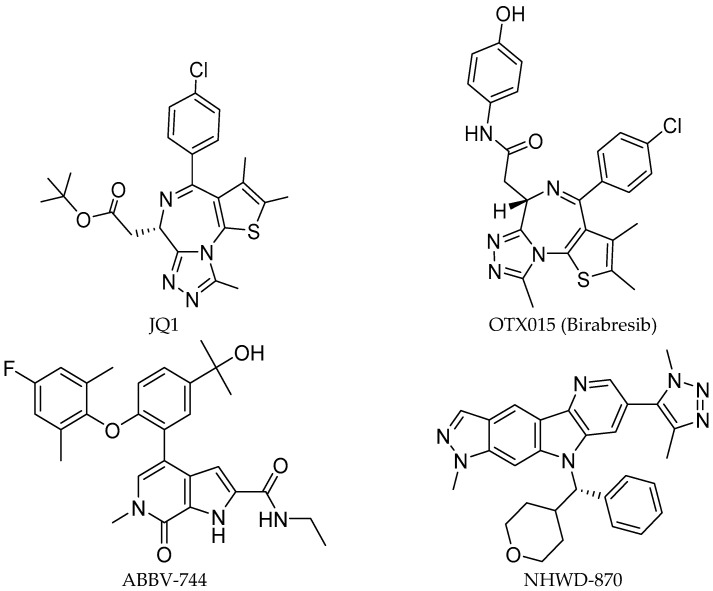
BET Bromodomain Inhibitors.

**JQ1** (Scheme 22) is a prototypical, noncovalent BET inhibitor featuring a thienotriazolodiazepine core and designed to occupy the KAc pocket of BET bromodomains and compete with acetyl-lysine recognition, thereby providing high selectivity toward BET proteins. In multiple myeloma and leukemia models, **JQ1** rapidly reduces MYC levels, induces G1 cell-cycle arrest, and promotes apoptosis, which laid the groundwork for the development of newer BET inhibitors with improved pharmacokinetic properties (e.g., I-**BET762**, **ABBV-744**, **NHWD-870**) (Scheme 22) [195]. JQ1 also suppresses signaling driven by the CRLF2/IL7R complex and the JAK-STAT5 axis in B-cell malignancy models in vitro and in vivo, which is linked to reduced cancer cell proliferation and survival [237].

**OTX015** (**MK-8628**, **birabresib**) (Scheme 22) retains the **JQ1** pharmacophore (a flattened triazolodiazepine core engaging the BD1/BD2 domains); however, modifications within peripheral fragments improved drug-like properties and enabled the development of an orally available BET inhibitor that subsequently progressed into clinical evaluation [238,239,240]. In a phase I study in patients with non-Hodgkin lymphoma and multiple myeloma, **OTX015** exhibited predictable pharmacokinetics, a class-consistent toxicity profile (thrombocytopenia, diarrhea, fatigue), and signals of antitumor activity, supporting the feasibility of systemic, long-term BET inhibition in humans [239,240,241]. In preclinical AML and acute lymphoblastic leukemia (ALL) models, **OTX015** decreased BRD2, BRD4, and c-MYC levels, which was associated with cell-cycle arrest, apoptosis induction, and inhibition of cancer cell growth [242].

Newer molecules, such as BD2-selective inhibitors (**ABBV-744**, apabetalone) and ligands selective toward either BD1 or BD2, have been developed through subtle modifications of the heteroaromatic core and the spatial arrangement of hydrogen-bond donors/acceptors, enabling diversification of pharmacological and toxicity profiles within the BET inhibitor class [243]. **NHWD-870** is a novel, potent BET inhibitor that suppresses tumor cell proliferation, among other mechanisms, by downregulating c-MYC and attenuating tumor cell– macrophage crosstalk; the study reported, inter alia, reduced CSF1 expression linked to inhibition of the BRD4/HIF1α axis, and in the evaluated models, **NHWD-870** showed higher activity than **OTX015** [244]. It is currently being evaluated in clinical trials (NCT06073938, NCT06527300) [245]. NHWD-870 was shown to limit melanoma invasion and metastasis via regulation of SPINK6, act synergistically with cytarabine, and display promising potential in the treatment of bone sarcomas [246].

**ABBV-744** is a highly BD2-selective inhibitor of BRD2, BRD3, and BRD4, with affinity hundreds of times higher than for BD1, translating into strong antiproliferative activity in AML and prostate cancer [243,247].

Despite intensive research, no BET inhibitor has received FDA approval for clinical use to date; all known compounds remain in preclinical development or at various stages of clinical trials [195].

##### PROTAC-Based BET Degraders

**Scheme 23:**
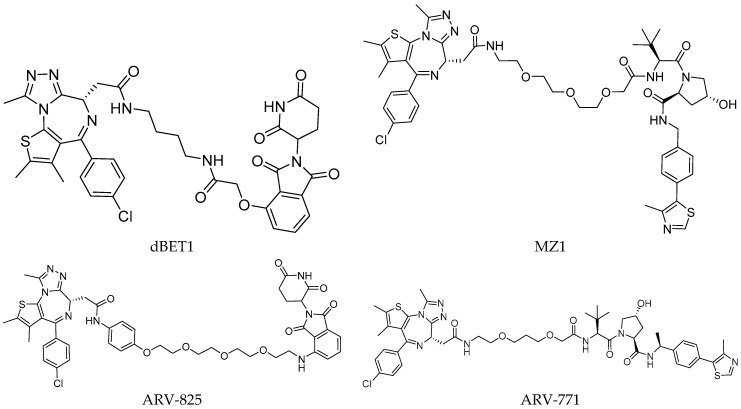
PROTAC-Based BET Degraders.

Resistance to BET inhibitors may arise, among others, from impaired ubiquitin-dependent turnover of BET proteins; in prostate cancer, SPOP (an E3 ligase component) mutations were shown to stabilize BRD4 by reducing its ubiquitination and degradation, thereby sustaining BRD4-dependent transcriptional programs, including the c-MYC axis [248]. PROTAC-based BET degraders (proteolysis targeting chimeras) address this limitation because they are bifunctional small molecules that recruit the target protein to an E3 ligase, forming a ternary complex that triggers polyubiquitination and proteasomal degradation of BET proteins [249,250,251,252]. The first BET-directed PROTAC, **dBET1** (a thalidomide-JQ1 conjugate), rapidly and selectively degraded BRD4 and reduced MYC levels [249]. Subsequent PROTACs, such as **MZ1** (which leverages the VHL E3 ligase) and **ARV-825** (Scheme 23), demonstrated strong antitumor activity in vitro and in vivo, including in hematological malignancy models [253,254,255,256,257]. Although PROTACs still face challenges related to cellular permeability, stability, and the potential for adverse effects associated with profound target degradation, overcoming these limitations may establish them as an important class of anticancer agents [252].

#### 2.5.3. IDH Inhibitors

Isocitrate dehydrogenases (IDH1 and IDH2) are key metabolic enzymes involved in the tricarboxylic acid cycle and redox homeostasis by catalyzing the conversion of isocitrate to α-ketoglutarate (α-KG) with concomitant NADPH production [258]. Under physiological conditions, IDH1 is localized in the cytosol and peroxisomes, whereas IDH2 resides in mitochondria; together, they support cellular pools of α-KG and NADPH, which are important, among others, for α-KG-dependent dioxygenases, including enzymes involved in DNA and histone demethylation [258]. In addition, IDH1/2 mutations confer a neomorphic enzymatic activity that reduces α-KG to the oncometabolite D-2-hydroxyglutarate (2-HG), leading to its marked accumulation in cancer cells, particularly in AML and gliomas [259]. The accumulation of 2-HG secondary to IDH mutations inhibits α-KG-dependent dioxygenases, resulting in dysregulated DNA and histone methylation and altered gene expression, which may promote tumor suppressor gene silencing and reinforcement of oncogenic programs [259]. Pharmacological inhibition of mutant IDH1/2 and subsequent reduction of 2-HG levels can partially reverse these epigenetic perturbations and induce differentiation of malignant cells, supporting the classification of IDH inhibitors as epigenetic regulators [259].

**Scheme 24:**
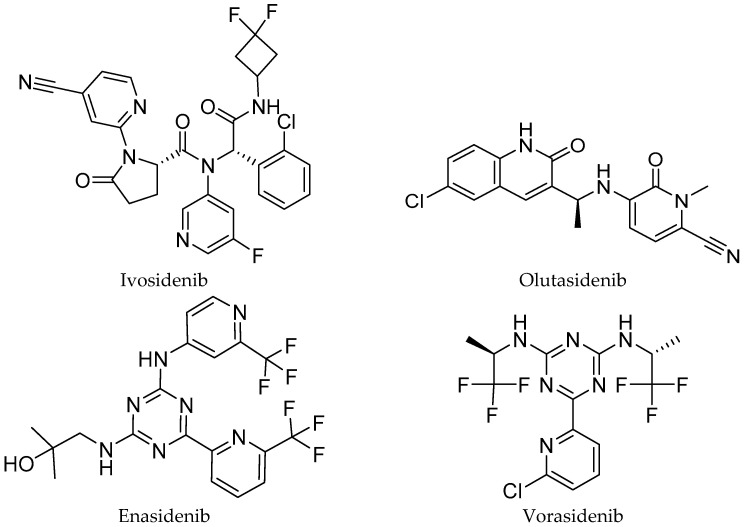
Inhibitors of IDH.

**Enasidenib** (**AG-221**) (Scheme 24) is an oral, selective, and potent inhibitor of mutant IDH2 with a triazine-based scaffold that binds the enzyme allosterically and stabilizes a nonproductive conformation, thereby limiting oncometabolite formation and reducing 2-HG levels [260,261]. **Enasidenib** received FDA approval in 2017 for adults with relapsed/refractory AML harboring an IDH2 mutation, and phase I/II studies confirmed clinically meaningful complete and partial responses as well as differentiation of leukemic blasts; a characteristic complication was the IDH differentiation syndrome (IDH-DS) [261]. IDH-DS is a drug-induced differentiation syndrome observed in AML patients treated with mutant IDH1/IDH2 inhibitors. It results from pharmacological suppression of 2-HG production, partial reversal of aberrant DNA and histone methylation, and release of the differentiation block, leading to rapid maturation of a large burden of leukemic cells. This process is accompanied by neutrophil expansion, cytokine release, and a systemic inflammatory response. IDH-DS typically occurs within the first weeks to 2 months of therapy and is diagnosed when at least two nonspecific symptoms coexist, such as dyspnea, fever without an identifiable infection, sudden weight gain, unexplained hypotension, acute kidney injury, pulmonary infiltrates, or pleural/pericardial effusions. It is estimated to affect approximately 10–20% of patients treated with **enasidenib or ivosidenib**, and prompt recognition followed by immediate initiation of glucocorticoids is critical to prevent fatal complications [262].

**Ivosidenib** (**AG-120**) (Scheme 24) is an oral, selective, allosteric inhibitor of mutant IDH1, designed as a small molecule that binds an allosteric pocket at the IDH1 dimer interface and potently suppresses 2-HG production in cellular models and in AML patient samples [263]. In 2022, it was approved by the FDA in combination with **azacitidine** for the treatment of patients with IDH1-mutated AML who are ≥75 years of age or have comorbidities that preclude intensive induction chemotherapy, based on the phase III AGILE trial (NCT03173248) [264].

**Olutasidenib** (**FT-2102**) (Scheme 24) is a potent, mutant-selective IDH1 inhibitor built on a quinolinone scaffold, developed through structure-based design to engage an allosteric pocket in IDH1, enable good oral bioavailability, and effectively suppress 2-HG production in cancer cells [265]. In the 2102-HEM-101 study in patients with relapsed/refractory IDH1-mutated AML, **olutasidenib** achieved complete or partial responses and durable remissions, which led to FDA approval in 2022 for this population; the safety profile was dominated by cytopenias, gastrointestinal adverse events, and a potential differentiation syndrome [266,267].

**Vorasidenib** (**AG-881)** (Scheme 24) is the first oral, brain-penetrant, dual inhibitor of mutant IDH1/2; crystallographic structural studies confirmed its binding to an allosteric site in IDH1/2, and in orthotopic glioma models, it reduced 2-HG production in tumor tissue by >97% [268]. **Vorasidenib** was approved by the FDA in 2024 for the treatment of adults and adolescents aged ≥12 years with World Health Organization (WHO) grade 2 astrocytoma or oligodendroglioma harboring an IDH mutation, based on the phase III INDIGO trial, which demonstrated an improvement in median PFS from 11.1 to 27.7 months versus placebo [269,270].

#### 2.5.4. Summary

Epigenetic regulators, including histone deacetylase (HDAC) inhibitors and bromodomain and extraterminal (BET) protein inhibitors, are increasingly important complements to anticancer therapy, providing molecular intervention points beyond canonical kinase-driven signaling pathways. This category can also encompass inhibitors of mutant IDH1/2, such as **ivosidenib**, **enasidenib**, **olutasidenib**, **and vorasidenib**. Despite distinct core scaffolds, these agents can be considered a coherent class of small-molecule, heteroaromatic allosteric IDH1/2 inhibitors, as they stabilize an inactive dimer conformation and suppress the neomorphic reduction of α-KG to 2-HG, thereby indirectly contributing to normalization of DNA and histone methylation patterns. Robust lowering of this oncometabolite has translated into clinically validated activity in IDH-mutant AML as well as in IDH-mutant tumors of the central nervous system. Hydroxamate HDAC inhibitors (e.g., **vorinostat**, **belinostat)** and non-hydroxamate inhibitors (e.g., benzamides, thiazolidines) rely on well-defined chemical motifs, including functional groups that serve as zinc-binding elements within the HDAC active site, enabling durable blockade of histone deacetylation, derepression of tumor suppressor gene programs, and induction of apoptosis in cancer cells [191,271]. In contrast, BET inhibitors (e.g., **JQ1**, **OTX015**) are small molecules that block the bromodomains of BET proteins (BRD2/3/4), which recognize acetylated lysine residues on histones and promote transcription of oncogenic drivers (including c-MYC). Their mechanism involves disruption of BET-chromatin engagement and attenuation of pro-proliferative transcriptional programs, supporting their development as epigenetic anticancer agents [272]. From a chemical design perspective, these compounds typically incorporate a heterocyclic core capable of occupying the bromodomain binding pocket, further tuned by aryl substituents and solubilizing groups to optimize pharmacokinetics.

Collectively, while epigenetic therapies do not replace kinase inhibitors or other agents targeting proliferative signaling, they represent a valuable complementary strategy. A detailed understanding of structure–activity relationships (SAR) and rational combination of epigenetic inhibitors with targeted therapies or immunotherapy will be central to future treatment regimens and to overcoming therapy resistance [273].

### 2.6. Protein–Protein Interaction (PPI) Inhibitors

The tumor suppressor p53 is physiologically restrained by MDM2, an E3 ubiquitin ligase that binds the N-terminal transactivation domain (TAD) of p53 and promotes its ubiquitination and proteasomal degradation, thereby forming a canonical negative feedback loop within the p53-MDM2 axis. MDM2 overexpression is associated with poor prognosis and has been linked to metastatic dissemination and EMT [274]. In many cancers, MDM2 overexpression suppresses p53 function despite the presence of intact TP53272. Moreover, MDM2 exhibits immunomodulatory activity: it can act as a tumor-associated antigen, shape T-cell and dendritic-cell populations, sustain STAT5 stability in CD8+ T cells, and modulate cytokine profiles and major histocompatibility complex class II (MHC-II) expression, making it a potential target for immunotherapeutic strategies [275,276,277,278,279].

Structurally, the N-terminal binding pocket of MDM2 is hydrophobic and recognizes the canonical p53 TAD motif (Phe19-Trp23-Leu26), which has provided the basis for designing small molecules that occupy this pocket and displace p53 [280]. These compounds, as MDM2 inhibitors, mimic the p53-MDM2 interaction in the form of a rigid, multi-aryl pharmacophore that competes with p53 for binding to MDM2 and thereby leads to stabilization and reactivation of functional p53 [274]. Therapeutically, blocking the MDM2–p53 interaction in cells harboring wild-type p53 stabilizes the tumor suppressor, increases the expression of effector genes, and triggers cell-cycle arrest and apoptosis [274]. From a clinical perspective, MDM2-p53 inhibitors are primarily considered in patients with preserved (wild-type) TP53 and documented MDM2 overexpression in the tumor, as supported by genomic and immunohistochemical analyses. Their use is justified in settings where the suppressor function of p53 is intact but excessively restrained by MDM2, an archetypal situation, for example, in dedifferentiated liposarcoma (DDLPS) [281,282]. Recent reviews emphasize the rapid evolution of this class (nutlins and newer chemotypes) and its progression into clinical trials, alongside ongoing evaluation of combination strategies (e.g., with kinase inhibitors and immunotherapy) [274,283]. Despite numerous clinical studies of MDM2 inhibitors and degraders, no agent of this type has yet received FDA approval due to the combination of limited efficacy in registration-oriented trials and clinically significant toxicity, predominantly hematologic, although promising clinical trials of new molecules are currently ongoing [283].

**Scheme 25:**
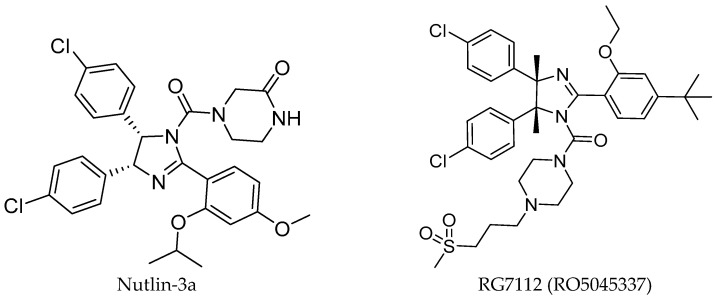
Scheme Imidazoline analogs inhibiting the p53–MDM2 interaction.

It has been shown that p53 binds to MDM2 via an α-helical motif, in which three hydrophobic residues (Phe19, Trp23, and Leu26) occupy a deep pocket in MDM2; mimicking this motif has become the basis for designing small molecules that inhibit the p53-MDM2 interaction [284,285,286]. **Nutlins** (**Nutlin-1, -2,** and **-3**) represent the first well-characterized class of potent, selective, non-peptidic small-molecule *cis*-imidazoline analogs, designed as mimetics of the key p53 residues (Phe19, Trp23, and Leu26). By occupying three hydrophobic subpockets within the N-terminal domain of MDM2, they block the p53-MDM2 interaction and reactivate the p53 pathway in cells harboring wild-type p53 [287,288]. **Nutlin-3a** (Scheme 25), the most active enantiomer of **Nutlin-3**, was not advanced into clinical development due to unfavorable pharmacological properties; however, it served as a template for the design of the 2,4,5-triarylimidazoline **RG7112** (**RO5045337**) (Scheme 25), which has been widely used in preclinical and clinical studies [289,290]. **RG7112** was evaluated in phase I trials (e.g., NCT00623870) in patients with relapsed or refractory AML, ALL, and CLL. Although p53 activation and tumor regressions were observed, development of the compound was discontinued due to limited bioavailability and hematologic toxicity, particularly neutropenia and thrombocytopenia [291]. The program was subsequently superseded by **idasanutlin** (**RG7388**), which offered an improved pharmacokinetic profile and selectivity.

**Scheme 26:**
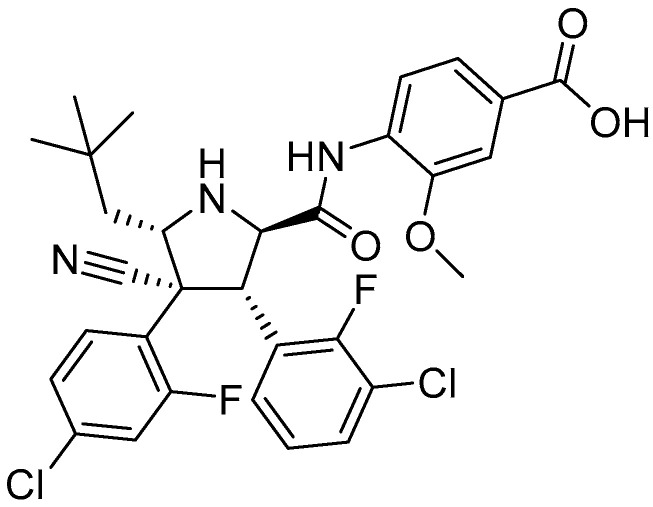
Chemical structure of **idasanutlin** (**RG7388**).

MDM2–ligand complex reflects a structure-guided optimization of binding potency. **Idasanutlin** (**RG7388**) (Scheme 26) is a second-generation, orally available MDM2 antagonist developed as a follow-up to early nutlin-like inhibitors (including **RG7112**), yet built on a distinct chemotype—a cyanopyrrolidine scaffold. The compound binds to the N-terminal pocket of MDM2 and mimics the spatial arrangement of the key residues within the helical p53 TAD motif (Phe19, Trp23, and Leu26), thereby efficiently displacing p53 from the MDM2 complex and functionally reactivating the p53 pathway [274]. Compared with nutlins, a hallmark design feature of idasanutlin is the “trans” orientation of the aryl rings within the pyrrolidine core together with additional interactions that stabilize the and selectivity while maintaining oral dosing feasibility [274,292]. Clinically, idasanutlin was evaluated, among others, in combination with cytarabine in patients with relapsed/refractory AML (the MIRROS trial); despite clear pharmacodynamic activation of the p53 pathway, adverse events such as neutropenia and thrombocytopenia were observed, and no overall survival advantage over cytarabine alone was demonstrated in the studied population, which contributed to the discontinuation of clinical development [293,294].

**Scheme 27:**
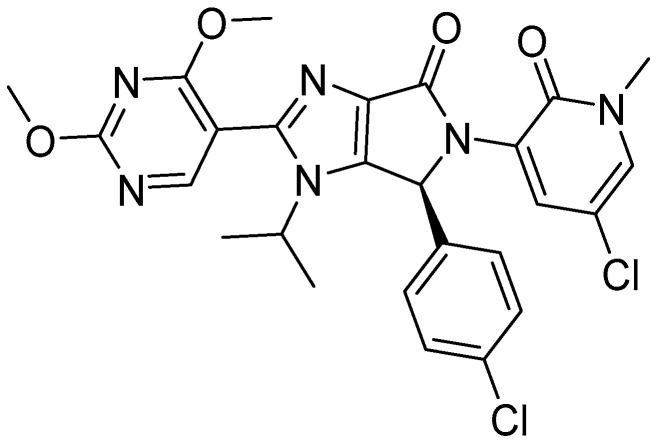
Chemical structure of **siremadlin** (**HDM201**).

The potential anticancer properties of p53-MDM2 inhibitors have driven the development of many new compounds that could find applications in oncology and are currently under investigation. **Siremadlin** (**HDM201**) (Scheme 27) is a small-molecule MDM2 antagonist from the imidazolo–pyrrolidinone class, designed to reactivate the p53 pathway by disrupting the p53-MDM2 interaction in tumors with wild-type TP53 [274,281]. The compound binds selectively to MDM2, applying the “central valine” concept, in which a flat, unsaturated core is positioned in close contact with the V93 residue within the MDM2 pocket that recognizes the p53 motif [295]. In practice, this results in an arrangement of the ligand’s hydrophobic fragments such that they functionally mimic the spatial organization of the key determinants of p53 binding in MDM2, thereby effectively competing with the helical p53 motif [295]. Preclinical studies suggested that a “pulsed” high-dose regimen of **siremadlin** may induce a stronger pro-apoptotic response than sustained low doses in cancer cells with wild-type p53, which is important for dose-schedule design in clinical studies [295]. In a phase I study (NCT02143635) in patients with advanced solid and hematologic malignancies with wild-type TP53, several dosing regimens were tested, and the primary objective was to assess dose-limiting toxicities (DLTs). In this study, despite adverse effects such as thrombocytopenia, siremadlin demonstrated an acceptable safety profile [281]. **Siremadlin** was also investigated as maintenance therapy in AML patients after stem cell transplantation, and the results indicate its efficacy and anti-leukemic activity, preventing disease relapse [296].

**Scheme 28:**
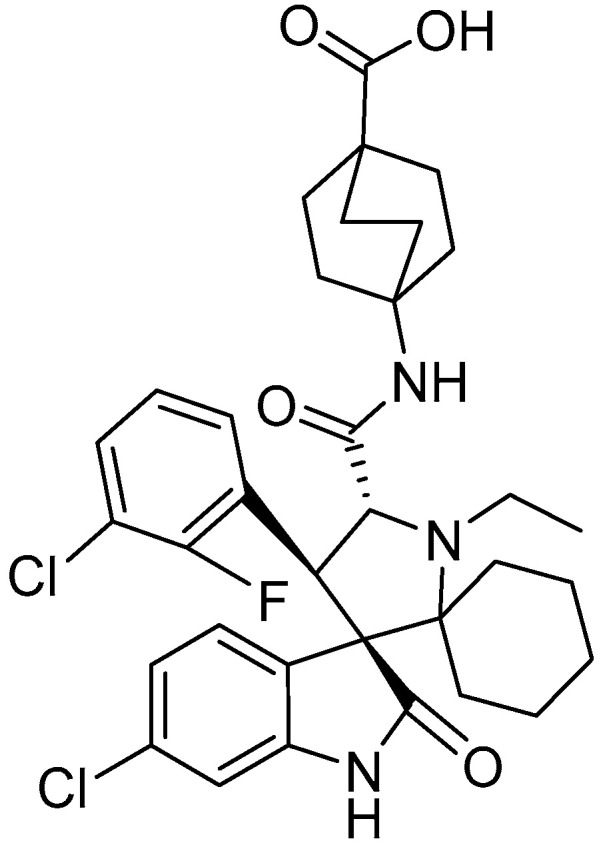
Chemical structure of **alrizomadlin** (**APG-115**).

**Alrizomadlin** (**APG-115)** (Scheme 28) is an oral, potent inhibitor of the p53-MDM2 interaction with a spiro-oxindole scaffold [274]. Early phase I studies demonstrated an acceptable safety profile in patients with advanced solid tumors, while ongoing phase II trials are evaluating APG-115 in adenoid cystic carcinoma (ACC), malignant peripheral nerve sheath tumor (MPNST), and other solid tumors (often in combination with PD-1 (Programmed Death-1) inhibitors), as well as in melanoma [297,298,299]. In monotherapy, **APG-115** showed measurable antitumor activity and was well tolerated in patients with squamous malignancies harboring wild-type p53 (SGC), with the strongest efficacy signal observed in the ACC subgroup [297]. Oral administration of **APG-115** was well tolerated and associated with antitumor activity, particularly in patients with wild-type TP53 [299]. Collectively, these findings suggest that **APG-115** is a promising candidate warranting further clinical investigation.

**Scheme 29:**
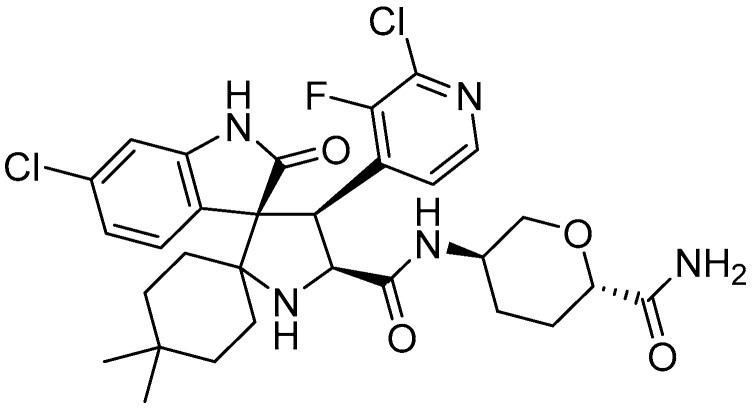
Chemical structure of **milademetan** (**DS-3032b**/**RAIN-32**).

**Milademetan** (**DS-3032b**/**RAIN-32**) (Scheme 29) is an orally available, potent MDM2 inhibitor with a complex heterocyclic architecture, designed to antagonize the MDM2-p53 interaction [300]. In the first-in-human study, an intermittent regimen of 260 mg once daily (Days 1–3 and 15–17 of a 28-day cycle) was established, mitigating dose-limiting hematologic toxicity while maintaining activity compared with continuous dosing [282]. Phase I/II trials evaluated milademetan in patients with advanced solid tumors, lymphomas, and acute myeloid leukemia, and a phase III study (NCT04979442) is comparing it with trabectedin in patients with dedifferentiated liposarcoma harboring MDM2 amplification and wild-type TP53 [301,302,303,304]. **Milademetan** prolonged PFS versus trabectedin without reaching statistical significance, and treatment-emergent adverse events were manageable with dose modifications [305].

**Scheme 30:**
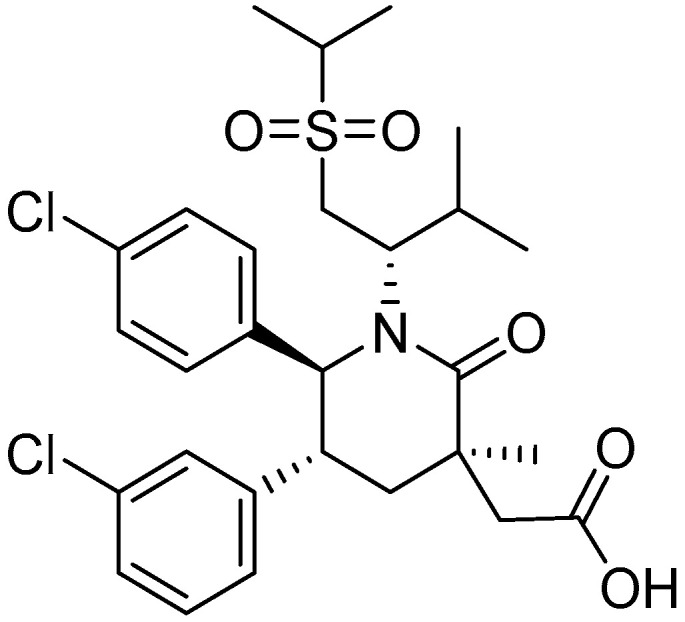
Chemical structure of **navtemadlin** (**KRT-232**).

**Navtemadlin** (**KRT-232**) (Scheme 30) is an orally available, selective MDM2 inhibitor that restores p53 function and is being clinically evaluated in acute leukemias and glioma, as well as in the global phase III BOREAS trial in relapsed or refractory myelofibrosis, representing one of the most clinically advanced programs within this drug class [306,307]. **Navtemadlin** has shown encouraging activity, particularly in myeloproliferative neoplasms with TP53-wt, where phase I/II studies reported reductions in splenomegaly, improvement of symptom burden, and objective responses when combined with other therapies [308,309].

**Scheme 31:**
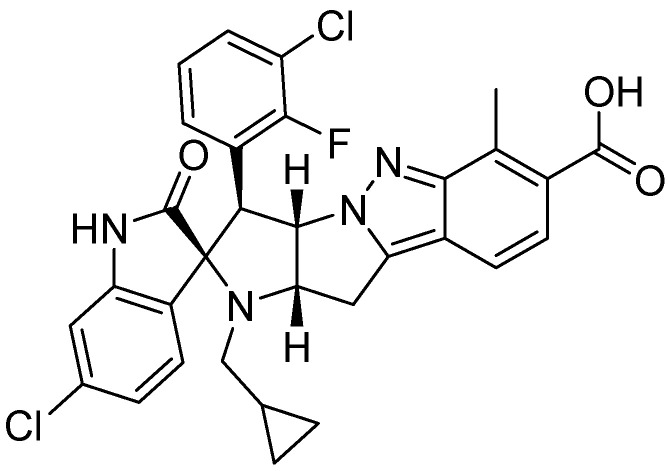
Chemical structure of **brigimadlin** (**BI 907828**).

**Brigimadlin** (**BI 907828**) (Scheme 31) is an orally available, small-molecule antagonist of the p53–MDM2 interaction with a spirooxindole scaffold, developed with intermittent high-dose dosing schedules in mind. In phase Ia/Ib studies, it demonstrated encouraging activity, particularly in DDLPS harboring MDM2 amplification, and it is currently being investigated in the randomized phase II/III Brightline-1 trial (vs. **doxorubicin** in DDLPS) as well as in phase II programs across other solid tumors, including biliary tract, pancreatic, lung, and gastric cancers. In parallel, early clinical studies (phase 0/Ia) are ongoing in wild-type TP53 glioblastoma [310,311,312,313,314].

Overall, MDM2 inhibitors represent one of the most thoroughly documented small-molecule strategies for pharmacologically restoring p53 function. Key clinical challenges include hematopoietic toxicity (thrombocytopenia and neutropenia), the need to select patients with wild-type TP53 and MDM2 expression, and resistance mechanisms (e.g., TP53 mutations or compensation by MDM4) [274,282]. Recent reviews recommend advancing rational combination regimens (e.g., with chemotherapy or signaling pathway inhibitors) and exploring MDM2-degradation platforms (e.g., PROTACs) to mitigate dose-limiting toxicity and overcome resistance [274,283]. In clinical development, the most advanced agents in this class have demonstrated robust biological activity in wt-TP53 tumor models and shown early clinical efficacy; however, their progress is frequently constrained by myelosuppression—primarily thrombocytopenia and neutropenia—which remains the most common dose-limiting adverse event [281,282,306].

#### Summary

p53–MDM2 inhibitors represent one of the most well-rationalized targeted strategies for cancers harboring wild-type TP53, as they enable pharmacological reactivation of the endogenous tumor-suppressive functions of p53; ongoing research is focused on improving safety and identifying biomarkers that will more precisely select patients most likely to benefit from this approach. Such inhibitors also illustrate that even extensive protein–protein interaction interfaces can be effectively disrupted by a carefully engineered combination of a flat core, three-dimensionally arranged aromatic groups, and a polar tail that modulates solubility and pharmacokinetics, thereby paving the way for other chemically complex PPI inhibitors in oncology [285,292].

## 3. Mechanisms of Resistance to Targeted Drugs

**Figure 1:**
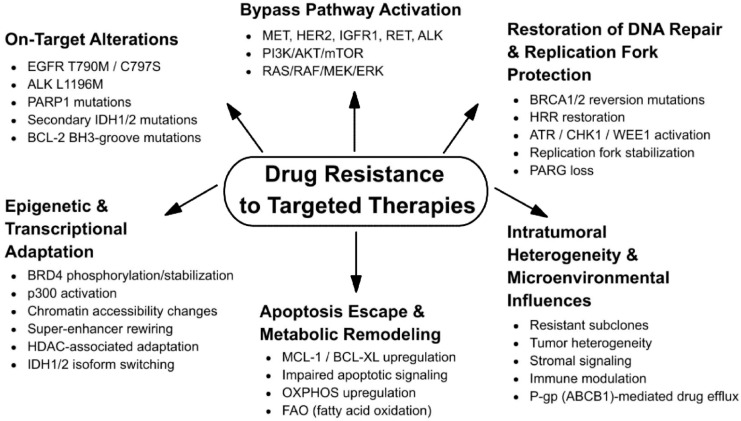
Schematic overview of major mechanisms of resistance to targeted anticancer therapies. Selected representative examples of on-target alterations, bypass signaling activation, DNA repair restoration, epigenetic adaptation, apoptosis escape, and microenvironment-driven resistance are shown.

Drug resistance to targeted therapies is multifactorial and encompasses alterations in the therapeutic target itself (on-target mechanisms), activation of bypass/compensatory signaling pathways (off-target mechanisms), intratumoral heterogeneity and microenvironmental influences, as well as modulation of antitumor immune responses, collectively limiting the durability of clinical benefit (Figure 1) [315,316]. In clinical practice, these resistance categories are observed across most major classes of targeted treatments, including tyrosine kinase inhibitors (TKIs), PARP inhibitors (PARPis), and epigenetic inhibitors, and their recognition has driven resistance-management strategies such as rational combinations, drug rotation, and sequential therapy [316].

### 3.1. EGFR (TKI): On-Target Alterations, Mutations, and Pathway Bypass

Resistance to tyrosine kinase inhibitors (TKIs) is an almost inevitable phenomenon and results from mutations within the ATP-binding site (e.g., EGFR T790M and C797S; ALK L1196M), activation of bypass pathways (e.g., MET, HER2, IGF1-R), compensatory activation of the PI3K/AKT or MAPK cascades, amplification or overexpression of target genes/proteins, and phenotypic plasticity of tumor cells [316]. One of the most frequent EGFR-dependent mechanisms of acquired resistance to third-generation EGFR TKIs, such as osimertinib, remains the EGFR C797S mutation in the kinase domain, which prevents formation of a covalent bond with the Cys797 residue within the ATP pocket and abolishes the activity of agents designed for activating EGFR alterations (e.g., L858R or exon 19 deletion) [316,317]. In parallel, signaling escape may occur through bypass activation—via amplification or acquisition of alterations in other receptor tyrosine kinases (RTKs) (e.g., MET, HER2, RET, ALK)—as well as reactivation of PI3K/AKT/mTOR and RAS/RAF/MEK/ERK nodes, potentially driven by PIK3CA mutations, PTEN loss, or enhanced upstream RTK signaling [316]. Clinically, these processes sustain proliferative and pro-survival signaling despite effective inhibition of the primary target, providing a rationale for combination regimens such as an EGFR TKI plus a MET or HER2 inhibitor, or vertical blockade along the EGFR-PI3K/AKT axis [316]. Similar compensatory pathway switching has been described for PI3K/AKT/mTOR inhibitors, where inhibition of one node can release feedback loops and induce secondary activation of the parallel MAPK pathway (or vice versa), necessitating multi-target strategies (e.g., PI3K/AKT plus MEK/ERK inhibition or PI3K/mTOR plus RTK blockade) rather than monotherapy [316,318,319]. In response to these processes, resistance-overcoming approaches are being pursued, including next-generation inhibitors (e.g., osimertinib targeting T790M and emerging compounds designed for C797S), allosteric and covalent inhibitors, PROTAC-based degraders, and rational combination therapies [316,320].

### 3.2. PARP (PARPi): Restoration of Homologous Recombination, Replication Fork Stabilization, and PARP Alterations

Resistance to poly(ADP-ribose) polymerase inhibitors (PARP inhibitors, PARPis) can arise through multiple routes that ultimately reduce tumor cell dependence on PARP inhibition-induced DNA damage. A major mechanism is reactivation of HRR, for example, via reversion mutations in HRR-related genes (e.g., BRCA1, BRCA2, RAD51C, RAD51D, PALB2) that restore protein function and re-establish double-strand break repair [321]. Resistance may also result from alterations in PARP1 itself (mutations reducing drug binding) and from loss of poly(ADP-ribose) glycohydrolase (PARG), which can sustain or restore poly(ADP-ribose) (PAR) levels and thereby attenuate PARPi cytotoxicity [322]. Clinically, detection of BRCA reversion mutations in circulating tumor DNA (ctDNA) may indicate emerging loss of PARPi sensitivity during treatment [323].

In addition, compensatory activation of other DDR pathways, particularly the ATR/CHK1/WEE1 axis, together with adaptation to chronic replication stress, can promote cell survival despite PARP blockade and provide a rationale for combination strategies (PARPi plus ATR/CHK1/WEE1 inhibitors) [324].

Another resistance layer involves mechanisms that limit the formation of lethal DNA lesions, including stabilization of replication forks; improved fork protection reduces secondary double-strand break formation and diminishes PARPi cytotoxicity [323].

Pharmacokinetic resistance is also relevant: overexpression of P-glycoprotein (P-gp), encoded by ABCB1 and also referred to as multidrug resistance protein 1 (MDR1), can lower intracellular PARPi exposure through increased drug efflux, contributing to treatment failure [146,323].

Collectively, these mechanisms may co-exist within a single tumor; therefore, dynamic assessment of HRD and DDR biomarkers (including BRCA reversion mutations in ctDNA, ABCB1 expression, and DNA damage signatures) and longitudinal monitoring of resistance evolution are critical for optimizing salvage treatment selection and for designing rational PARPi-based combination strategies [146,322,323].

### 3.3. BET/HDAC/IDH (Epigenetic Regulators): Adaptive Transcriptional Reprogramming and Compensatory Mechanisms

Resistance to BET inhibitors (bromodomain and extraterminal proteins; mainly BRD2/BRD3/BRD4) is most often adaptive and arises from transcriptional-epigenetic reprogramming rather than from mutations within BET bromodomains [194,325]. In experimental models, compensatory activation of alternative growth programs has been observed (e.g., rewiring of receptor tyrosine kinases and PI3K/RAS signaling) as well as cytokine signaling, which can promote BRD4 phosphorylation and stabilization and consequently reduce sensitivity to BET inhibition [195]. This helps explain the limited efficacy of BET inhibitor monotherapy and provides a rationale for evaluating combination regimens (e.g., BET inhibitors plus tyrosine kinase inhibitors, PARP inhibitors, BCL-2 inhibitors, or chemotherapy), an approach supported by clinical studies [195]. An important mechanism of early BET inhibitor tolerance also involves p300 (EP300): its activity enables rapid induction of an alternative gene-expression program that compensates for BET blockade and allows tumor cells to maintain pro-survival and proliferative signaling, thereby promoting resistance (initially acute and subsequently more durable) [325].

Resistance to histone deacetylase inhibitors (HDACis) can arise from increased tolerance of DNA damage through intact cell-cycle checkpoints, particularly those dependent on checkpoint kinase 1 (ChK1) [326]. In addition, tumor cells may evade apoptosis via overexpression of anti-apoptotic BCL-2 family proteins and compensatory activation of pro-survival pathways such as NF-kB [327]. Pharmacokinetic resistance can also contribute: HDACis may increase expression of the ABCB1 efflux transporter encoding P-glycoprotein, in part through increased STAT3 activity, thereby lowering intracellular drug exposure and promoting loss of response [328].

More broadly, HDACi resistance frequently reflects epigenetic rewiring of gene expression through altered chromatin accessibility at promoters, enhancers, and super-enhancers, together with modulation of non-histone substrates, which collectively reshape cellular responses to therapy; therefore, careful selection of the HDACi modality and a treatment strategy targeting dominant adaptive mechanisms is critical [329].

Resistance to IDH inhibitors is most often acquired and is associated with the emergence of secondary mutations in IDH1/2 that reduce sensitivity to ivosidenib/enasidenib, restore 2-HG production, and abrogate the differentiation effect induced by the inhibitor in leukemic cells [330,331]. A second well-described mechanism is so-called isoform switching: during therapy, a new mutation arises in the alternative isoform (e.g., from IDH1mut to IDH2mut or vice versa), enabling the tumor to regain the ability to synthesize 2-HG despite selective inhibition of the original mutant enzyme [332].

Collectively, these mechanisms underscore that resistance to epigenetic inhibitors is dynamic and multilayered, and overcoming it requires rational combination strategies that concurrently target the primary lesion and key compensatory pathways.

### 3.4. MDM2-p53 (PPI): Selective Pressure on TP53 and MDM4

MDM2 inhibitors in tumors with wild-type TP53 exert selective pressure that can favor the emergence of TP53-mutant clones and limit response durability, thereby supporting the development of intermittent dosing schedules and combination strategies (e.g., with cytotoxic agents, tyrosine kinase inhibitors, or immunotherapy) [274,333]. Overexpression of MDM4 (MDMX), an MDM2 homolog, may further attenuate responses to selective MDM2 blockade by functionally suppressing p53 activity and/or reinforcing its inactivation within the MDM2/MDM4 axis, which is considered a key limitation of monotherapy and provides a rationale for dual-targeting approaches [333]. Resistant clones have also been reported to display adaptive activation of pro-survival pathways (e.g., PI3K/AKT/mTOR) and alterations in the apoptotic machinery, which may reduce the ability of p53 to trigger cell death despite effective disruption of the p53-MDM2 interaction [274]. In line with this, the clinical development of MDM2 inhibitors currently focuses on strategies that limit selection of TP53-mutant-positive clones, including intermittent schedules, combinations with DNA-damaging chemotherapy, tyrosine kinase inhibitors, or immunotherapy, as well as dual-targeting approaches, i.e., concurrent inhibition of MDM2 and MDM4, which may reduce the risk of escape via MDM4-dependent mechanisms [274,333]. Targeting the p53-MDM2 interaction represents a fundamentally different therapeutic strategy compared with kinase inhibition, as it involves disruption of protein–protein interactions rather than enzymatic blockade. Despite a strong preclinical rationale, clinical translation has proven challenging. The efficacy of MDM2 inhibitors is restricted to tumors retaining wild-type TP53, and dose-limiting toxicities, particularly hematologic adverse effects, have limited their therapeutic window. In addition, tumors may develop resistance through alterations in downstream apoptotic pathways or selection of TP53-mutant clones. These limitations highlight the complexity of reactivating tumor suppressor pathways compared with inhibiting oncogenic drivers.

### 3.5. Resistance to Inhibitors of Anti-Apoptotic Proteins (BCL-2, MCL-1)

Resistance to BH3 mimetics, such as venetoclax, may be present either before treatment initiation or emerge during therapy. Primary resistance reflects the fact that some tumors rely from the outset on anti-apoptotic proteins other than BCL-2, most notably MCL-1 or BCL-XL, which markedly reduces sensitivity to BCL-2 inhibition. This phenomenon has been described in analyses of tumor cell survival and adaptation mechanisms, showing that high MCL-1 or BCL-XL expression can predict lack of response to venetoclax therapy [124]. During treatment, acquired resistance may develop, most commonly through compensatory upregulation of MCL-1 or BCL-XL, which functionally substitutes for inhibited BCL-2 and enables continued suppression of pro-apoptotic signaling despite drug exposure [124]. Another mechanism involves mutations within the BH3-binding groove of BCL-2 that reduce venetoclax binding affinity and thereby attenuate its activity; clinical reports indicate that such structural alterations in BCL-2 can lead to loss of therapeutic efficacy [334]. An important component of acquired resistance is also remodeling of mitochondrial metabolism. Under therapeutic pressure, tumor cells may increase oxidative phosphorylation (OXPHOS) and fatty acid oxidation (FAO), which enhances their bioenergetic capacity and supports maintenance of anti-apoptotic function despite BCL-2 blockade; these changes have been highlighted in reviews of venetoclax resistance mechanisms [334,335].

Accordingly, combination strategies are gaining importance. Venetoclax combinations with MCL-1 inhibitors, epigenetic modulators that reduce MCL-1 levels, or agents targeting metabolic pathways (e.g., OXPHOS or FAO inhibitors) have been shown to overcome both primary and acquired resistance [124,334,335]. Importantly, these resistance mechanisms rarely occur in isolation but instead reflect dynamic evolutionary processes within tumors. Clonal selection under therapeutic pressure leads to the expansion of resistant subpopulations, while non-genetic mechanisms such as transcriptional reprogramming and microenvironmental influences further contribute to adaptive resistance. From a clinical perspective, this complexity explains why targeting a single pathway is often insufficient and supports the increasing use of combination therapies designed to block multiple signaling nodes simultaneously.

### 3.6. Summary

Resistance to targeted therapies is a multifactorial and dynamic phenomenon that arises from the interplay of genetic alterations and functional adaptations in tumor cells. It may involve changes in the therapeutic target that reduce drug efficacy, as well as activation of bypass and compensatory signaling pathways that sustain pro-survival and proliferative cues despite inhibition of the primary target. Phenotypic and epigenetic plasticity, rewiring of DNA damage response networks, metabolic remodeling, and microenvironmental influences can further promote the selection of clones that are better adapted to therapeutic pressure. Consequently, the greatest clinical potential lies in strategies that account for the evolutionary nature of resistance, including rational combination regimens, sequential and adaptive approaches, and longitudinal molecular monitoring during treatment to identify the dominant escape mechanism early and adjust therapy accordingly.

## 4. Examples of New Inhibitors Approved by the FDA

In recent years, a clear acceleration has been observed in the approval of new targeted therapies in oncology, as reflected in FDA decisions from 2022 to 2025 (Table 2, Table S2). During this period, numerous small-molecule inhibitors were authorized against both established and emerging molecular targets, including tyrosine and serine-threonine kinases (e.g., EGFR, ALK, ROS1, RET, MET, BRAF), key signaling pathway components (e.g., MEK, PI3K, AKT), DNA repair enzymes (PARP1/2), and selected epigenetic inhibitors.

Many of these agents represent next-generation inhibitors developed in response to increasing molecular resistance, particularly within the EGFR-TKI and MAPK inhibitor landscapes. In addition, several targeted therapies were approved as rational combinations (e.g., **dabrafenib** plus **trametinib**, **sotorasib** plus **panitumumab**), underscoring the growing role of multi-pathway blockade strategies in the management of resistant or advanced malignancies.

The small-molecule therapies approved by the FDA in 2022–2025 and summarized in the table reflect several dominant trends in contemporary oncology. First, the continued expansion of tyrosine kinase and serine-threonine kinase inhibitors is evident, spanning established targets (EGFR/ALK/RET/MET) and newer agents directed against BRAF, MEK, PI3K, and AKT. Second, the number of drugs rationally developed to address specific resistance mechanisms has increased (e.g., **sunvozertinib** for EGFR exon 20 insertions, **taletrectinib** for ROS1, and **inavolisib**/**capivasertib** for PI3K/AKT pathway alterations).

Third, within the DNA damage repair space, PARP inhibitors have further consolidated their role, both as monotherapy (**talazoparib**) and in combination regimens (**niraparib** plus **abiraterone**). Overall, recent FDA approvals highlight a steady shift away from targeted therapies toward more precise, biomarker-driven patient selection and toward rational combinations that intercept adaptive signaling routes and may help delay or mitigate resistance.

## 5. Biological Drugs

Biological drugs represent the second major pillar of contemporary targeted therapy, alongside small-molecule inhibitors of kinases and other enzymes. They include monoclonal antibodies directed against receptors or soluble ligands involved in growth signaling pathways (e.g., EGFR, HER2, VEGF, and MET), as well as more complex ADCs that enable selective delivery of cytotoxic payloads to cancer cells (Table S3). In contrast to classical tyrosine kinase inhibitors (TKIs), biologics primarily act on extracellular receptor domains or within the tumor microenvironment, thereby not only blocking proliferative signaling but also modulating immune responses and inhibiting angiogenesis. In recent years, advances in antibody engineering, receptor fusion proteins, and ADC technologies have translated into numerous clinical approvals, particularly in tumors refractory to conventional kinase inhibitors [402,403,404].

Biological therapies in oncology include monoclonal antibodies, BsAb, ADCs, and adoptive cell therapies (CAR-T and TIL). A shared feature of these modalities is the selective recognition of tumor antigens or key regulators of immune responses, enabling a targeted enhancement of anticancer activity with a relative reduction in systemic toxicity. Immune checkpoint inhibitors (PD-1, PD-L1, and CTLA-4—cytotoxic T-lymphocyte-associated protein 4) relieve T-cell suppression within the tumor microenvironment, leading to reinvigoration of effector responses against malignant cells, and have become a cornerstone of therapy for many solid and hematologic malignancies [405,406]. Anti-PD-1 antibodies, such as **nivolumab** and **pembrolizumab**, have demonstrated clinical efficacy, among others, in melanoma, NSCLC, RCC, head and neck cancer, and MSI-H/dMMR tumors (microsatellite instability-high/mismatch repair deficiency), while treatment responses correlate with biomarkers such as PD-L1 expression—elevated PD-L1 expression promotes an “exhausted” T-cell phenotype, which is associated with impaired immune activity and facilitates immune evasion and tumor progression [405]. Anti-PD-L1 antibodies (e.g., **atezolizumab** and **durvalumab**) and anti-CTLA-4 therapy (**ipilimumab**) provide complementary targeting and, in several indications, are used in combination regimens (e.g., melanoma or NSCLC), translating into improved survival at the cost of a higher risk of immune-related toxicities [405].

Innovative CAR-T therapies involve the genetic reprogramming of autologous T lymphocytes, typically using viral vectors, to express a chimeric antigen receptor that recognizes a target antigen on tumor cells, enabling specific recognition and direct lysis of malignant cells upon target engagement [407,408]. Approved CAR-T products, including **tisagenlecleucel**, **axicabtagene ciloleucel**, **lisocabtagene maraleucel**, and **ciltacabtagene autoleucel**, primarily target CD19 or B-cell maturation antigen (BCMA) and achieve high rates of deep responses in patients with relapsed or refractory lymphomas and multiple myeloma after multiple prior lines of therapy [409,410].

Tumor-infiltrating lymphocyte (TIL) therapy is another emerging approach in oncology. It involves harvesting a tumor specimen, isolating endogenous T cells present within the tumor microenvironment, expanding them ex vivo in the presence of T-cell growth factors and interleukin 2 (IL-2), and reinfusing the expanded cell product into the patient [411]. The resulting product comprises a polyclonal T-cell population capable of recognizing multiple tumor antigens, including neoantigens, which may increase the likelihood of overcoming tumor heterogeneity and the outgrowth of resistant clones compared with single-antigen targeting strategies such as CAR-T therapy [412]. An example of this strategy is **lifileucel**, which was approved by the FDA in 2024 for the treatment of advanced melanoma. **Lifileucel** is generated by isolating TILs from the tumor, extensively expanding them ex vivo in the presence of IL-2, and reinfusing them into the patient, and it has been associated with durable responses in a substantial proportion of patients with advanced melanoma after failure of prior treatment lines [412].

BsAb, including T-cell-engaging bispecific antibodies, can simultaneously bind CD3 on T lymphocytes and a tumor-associated antigen (e.g., CD19, CD20, or BCMA) and act as a molecular bridge that brings effector cells into close proximity with tumor cells, thereby triggering T cell-dependent cytotoxicity without the need for genetic modification of patient-derived cells [413]. The efficacy of T-cell-engaging bispecific antibodies depends on efficient immune synapse formation between T cells and tumor cells, which is influenced by antigen density, epitope accessibility, and the spatial configuration of the bispecific construct. Different structural formats (e.g., IgG-like vs. fragment-based constructs) vary in half-life, tissue distribution, and the need for continuous administration. Importantly, BsAbs can activate polyclonal T-cell populations independently of native T-cell receptor specificity, thereby bypassing the requirement for antigen presentation via major histocompatibility complex molecules [414].

This class includes agents approved by the FDA in 2022, such as **mosunetuzumab** (CD3xCD20) for relapsed/refractory follicular lymphoma, **teclistamab** (CD3xBCMA) for relapsed/refractory multiple myeloma, and **tebentafusp** (CD3xgp100) for uveal melanoma; in parallel, ongoing research is exploring additional target combinations as well as tri- and tetraspecific formats, which may further broaden therapeutic options and enhance tumor cell elimination [413,415,416].

Clinically approved BsAbs have demonstrated high response rates in heavily pretreated patients with lymphoma and multiple myeloma, respectively, supporting their role as off-the-shelf alternatives to cellular therapies. **Tebentafusp**, a gp100-directed T-cell receptor-based bispecific, represents a distinct approach that redirects T cells against peptide–HLA complexes and has shown survival benefit in uveal melanoma, a disease historically resistant to systemic therapies [417,418]. In solid tumors, approved examples include **amivantamab** used in selected forms of non-small cell lung cancer with EGFR alterations, **tebentafusp** for metastatic uveal melanoma, and **zanidatamab** for HER2-positive biliary tract cancer [419].

However, the limitations of this class of agents primarily include on-target/off-tumor toxicity, immunogenicity, and adverse events associated with excessive immune activation, particularly cytokine release syndrome and immune effector cell-associated neurotoxicity syndrome (ICANS) [419]. Infections, neutropenia, lymphopenia, and hypogammaglobulinemia also remain important clinical concerns, especially for therapies targeting BCMA or CD20. The toxicity profile may depend on the target antigen—for example, gp100-targeted tebentafusp may induce adverse effects manifested mainly by skin-related events, such as rash and pruritus, often accompanied by fever, due to the presence of gp100-positive melanocytes in the skin. In solid tumors, additional challenges include dense stroma, poor perfusion, and impaired T-cell infiltration, which partly explains the greater success of these therapies in hematologic malignancies. These limitations may be partially mitigated by appropriate molecule design, step-up dosing, dexamethasone premedication, supportive care, vaccination, antimicrobial prophylaxis, and combination strategies with other immunotherapies, including anti-CD38 antibodies or immune checkpoint inhibitors [419].

ADCs combine the antigen selectivity of an antibody with a highly cytotoxic payload that is released after complex internalization and linker cleavage or degradation, enabling selective delivery of a chemotherapeutic agent to tumor cells while limiting systemic toxicity [402,420]. Following antigen binding, the conjugate–antigen complex is internalized via endocytosis and subsequently trafficked to endosomal-lysosomal compartments, where payload release occurs. Tumor-associated triggers that can promote drug release include low pH, reactive oxygen species (ROS), and tumor-associated enzymes. Once released, the payload induces tumor cell killing depending on its mechanism of action, for example, through DNA damage, cell-cycle arrest, or inhibition of mitosis [402]. The therapeutic activity of ADCs is critically dependent not only on target antigen expression but also on antigen density, internalization efficiency, and intracellular trafficking. Highly internalizing targets (e.g., HER2 or TROP2) favor effective payload delivery, whereas low internalization rates may limit ADC efficacy despite detectable antigen expression. Furthermore, linker design represents a key determinant of therapeutic index: cleavable linkers enable payload release in response to tumor-associated conditions (e.g., low pH or protease activity), while non-cleavable linkers rely on complete lysosomal degradation of the antibody component. Importantly, membrane-permeable payloads can mediate a “bystander effect,” allowing cytotoxic activity to extend to neighboring antigen-low or antigen-negative tumor cells, which may partially overcome intratumoral heterogeneity [421,422,423,424].

Since the clinical introduction of the first ADC, **gemtuzumab ozogamicin** (Mylotarg) targeting CD33 in AML in 2000, the ADC field has expanded rapidly, and to date, 15 molecules of this class have received regulatory approvals across both solid and hematologic malignancies [425]. These include, among others, additional ADCs approved by the FDA in 2025, such as **datopotamab deruxtecan** (Datroway; a TROP2-directed ADC) for hormone receptor-positive/human epidermal growth factor receptor 2-negative (HR+/HER2-) breast cancer and, under accelerated approval, for EGFR-mutated NSCLC, **telisotuzumab vedotin** (Emrelis; a c-Met-targeting ADC) for NSCLC with high c-Met protein overexpression, and **belantamab mafodotin** (Blenrep; a BCMA-directed ADC) as part of a combination regimen for relapsed/refractory multiple myeloma [426,427,428,429]. In parallel, target antigen selection has shifted from hematologic markers (CD30, CD22, CD33, and CD79b) toward more frequently expressed targets in solid tumors (HER2, TROP2, Nectin-4, and folate receptor alpha (FRα)), facilitating broader clinical applicability 399. Payload design has also evolved: beyond microtubule inhibitors (MMAE and DM1), ADCs increasingly incorporate DNA-damaging agents (calicheamicins and pyrrolobenzodiazepines (PBDs)) and topoisomerase I inhibitors (SN-38 and deruxtecan), highlighting a shift from relatively simple cytotoxic carriers to mechanistically diversified, tumor-directed therapies [402,425].

The efficacy of antibody–drug conjugates (ADCs) may be limited by both resistance and mechanism-related toxicity. The main resistance mechanisms include downregulation or loss of target antigen expression, impaired internalization and lysosomal processing, increased activity of drug efflux pumps, and reduced tumor cell sensitivity to the delivered payload [430]. The major safety concerns include myelosuppression, interstitial lung disease reported for some topoisomerase I-based ADCs, and ocular adverse events [431]. Addressing these challenges requires careful patient selection, appropriate biomarker use, optimization of ADC design, and rational combination strategies [430,431].

The role of biologic agents in targeted therapy continues to expand, and many have become central components of standard treatment regimens across a broad range of indications, from HER2-positive breast cancer and NSCLC to lymphomas and rare malignancies. Their distinctive mechanisms of action—including precise recognition of tumor-associated antigens and the ability to modulate immune responses—enable effective treatment in settings where small molecules are insufficient or readily drive resistance. Moreover, the development of ADCs and BsAb antibodies is paving the way for even more selective and synergistic therapeutic approaches.

Integrating biologics into the targeted therapy landscape supports a coherent treatment paradigm based on the precise identification of key molecular alterations and the selection of agents that intercept these drivers at the level of receptors, enzymes, or cell–cell interactions. Consequently, biologic therapies are no longer merely complementary to small-molecule drugs but represent a fully established and essential pillar of personalized oncology.

## 6. Innovative Molecular Targets in Preclinical Research and Early-Phase Clinical Trials

Despite the impressive progress in targeted therapies and immunotherapy over the past two decades, most approved anticancer drugs still converge on a relatively limited set of classical targets, including tyrosine kinases and components of the PI3K/AKT/mTOR and MAPK pathways, antiapoptotic BCL-2 family proteins, DNA repair factors (including PARP), and epigenetic regulators such as HDACs and BET family proteins. In many patients, however, the initial response is transient, and the development of acquired resistance remains one of the major limitations of contemporary precision-oriented therapies. At the same time, an expanding body of evidence is elucidating additional mechanisms of tumor plasticity, including rewiring of cell-death programs, metabolic reprogramming, and complex interactions with the immune system.

In this context, the search for innovative molecular targets is becoming increasingly important, not only to move beyond well-established drug classes discussed above, but also to open routes toward entirely new families of therapeutic agents. In this chapter, we define “innovative” targets as those that are currently at the preclinical stage or in early clinical development, fall outside the previously discussed categories, and are supported by convincing mechanistic evidence together with initial signals of antitumor activity, indicating realistic translational potential.

Accordingly, this section focuses on several selected, representative areas: inhibitors of protein–protein interactions within the MDM2-p53 axis and compounds that reactivate mutant p53, modulation of ferroptosis through targeting FSP1/AIFM2, emerging immuno-oncology targets (CD47-SIRPα, TIGIT, the STING pathway), and attempts to directly silence the MYC proto-oncogene pharmacologically. These examples do not exhaust the full spectrum of targets currently under investigation, but they illustrate the direction of molecular oncology—from relatively straightforward kinase inhibition toward pharmacological engagement of previously “undruggable” structures, complex protein–protein interaction networks, and specialized cell-death programs.

### 6.1. Inhibitors of MDM2-p53 and MDM4-p53 Protein–Protein Interactions (PPIs)

**Scheme 32:**
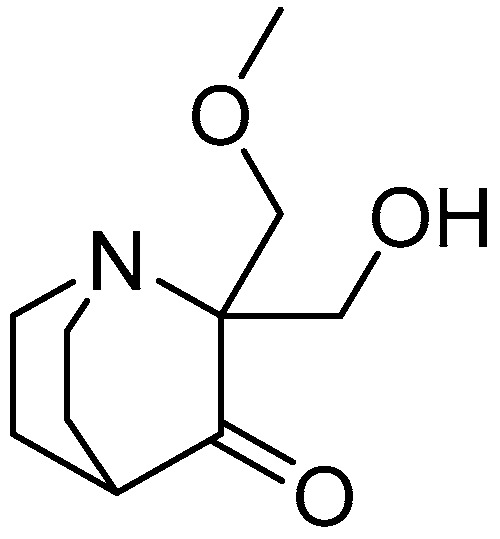
Chemical structure of **eprenetapopt** (**APR-246**).

Excessive activation of the E3 ubiquitin ligase MDM2 is one of the key mechanisms underlying functional attenuation of wild-type p53 in many cancers, particularly in tumors with MDM2 amplification and retained TP53-WT. Inhibition of the MDM2-p53 interaction results in p53 stabilization and can trigger apoptosis and cell-cycle arrest in cancer cells [274]. Despite the advanced stage of clinical development of these agents, no MDM2 inhibitor has been approved by the FDA for cancer treatment to date, as highlighted in the most recent clinical reviews [274]. This indicates that the MDM2-p53 PPI remains an innovative, clinically unexploited molecular target that may ultimately define a distinct therapeutic class in the future. A complementary approach involves reactivation of mutant p53. The small molecule **eprenetapopt** (**APR-246**) (Scheme 32), through binding to cysteine residues in p53, stabilizes its conformation and partially restores transcriptional function in cells harboring missense mutations; the compound has been investigated, among others, in patients with myelodysplastic syndromes (MDS) and TP53-mutant leukemias [432]. The clinical efficacy of this strategy was supported by the phase Ib/II trial NCT03072043, in which eprenetapopt combined with azacitidine in patients with TP53-mutant MDS and oligoblastic AML achieved a complete remission rate of 44% and an overall response rate of 71%, with an acceptable safety profile [432].

**Scheme 33:**
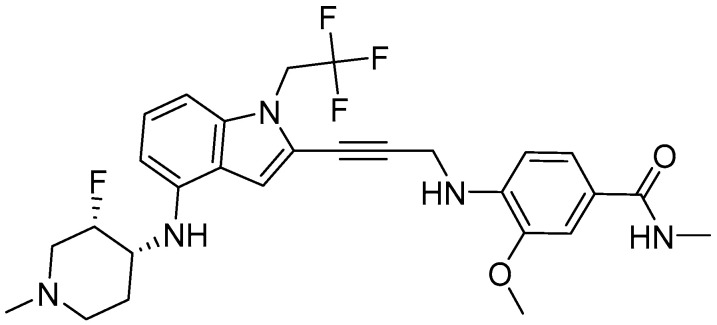
Chemical structure of **rezatapopt** (**PC14586**).

An even more selective example is **PC14586** (**rezatapopt**) (Scheme 33), a molecule specifically designed to stabilize and reactivate the p53 Y220C mutant; in a phase I study in patients with solid tumors harboring the TP53 Y220C mutation, antitumor activity and an acceptable safety profile were reported [433,434]. The ongoing phase 2 PYNNACLE study, designed as a registrational trial, aims to further evaluate the efficacy and safety of **rezatapopt** monotherapy in patients with locally advanced or metastatic solid tumors with a TP53 Y220C mutation and KRAS WT status, and participant enrollment is currently actively underway [435].

**Scheme 34:**
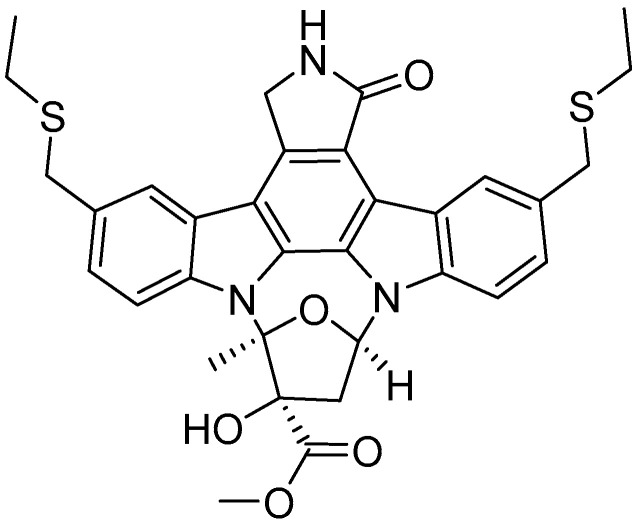
Chemical structure of **CEP-1347**.

Collectively, these data indicate that the MDM2-p53 axis and selective reactivation of mutant p53 represent one of the most promising, yet still clinically unoccupied, groups of molecular targets. A key limitation of this innovative therapeutic approach is hematologic toxicity resulting from p53 activation in healthy tissues, which leads to dysregulation of cell-cycle control. This effect is particularly pronounced in the bone marrow, where MDM2 inhibition and p53 induction disrupt hematopoiesis [436]. An additional challenge is overexpression of MDM4 (MDMX), a homolog of MDM2, which in cancer cells may compete with MDM2 for p53 binding and thereby selectively suppress p53 activity [437,438]. However, unlike MDM2 inhibitors, no selective small-molecule MDM4 (MDMX) inhibitors have been developed or approved to date. The most clinically advanced agent targeting MDM4 remains the peptide **ALRN-6924,** a dual MDM2/MDM4 inhibitor that disrupts the p53-MDM2/MDM4 interaction [439]. It mimics the N-terminal domain of p53 and binds with high affinity to both MDM2 and MDM4, thereby activating p53 signaling in cells with wild-type TP53 [439]. In the phase I trial NCT03725436 in patients with wild-type TP53 solid tumors, **ALRN-6924** showed biological activity, and it is also being evaluated in phase II studies, including NCT04022876 in small-cell lung cancer and NCT02264613 in solid tumors and lymphomas; however, it remains an investigational agent [437,439,440]. Compounds primarily targeting MDM4, such as **CEP-1347** (Scheme 34), represent a potential direction for anticancer drug development; nevertheless, they are still at the preclinical and experimental stages [441,442].

### 6.2. Ferroptosis Modulation—FSP1/AIFM2 as an Emerging Druggable Target

Ferroptosis is a regulated form of cell death driven by iron dependency and lipid peroxidation, which may selectively eliminate cancer cells that are resistant to conventional therapies [443,444]. Glutathione peroxidase 4 (GPX4) is a key guardian against ferroptosis; however, in recent years, AIFM2/FSP1 (ferroptosis suppressor protein 1) has been identified as an additional, GPX4-independent protective system based on the NADPH-dependent reduction of ubiquinone (CoQ10) and vitamin K (VK) to their reduced forms, which act at the plasma membrane as scavengers of lipid radicals (the FSP1-dependent CoQ10/VK axis) [443,445,446,447]. Overexpression of FSP1 in cancer cells increases resistance to ferroptosis induction through the GPX4 pathway as well as via other mechanisms, and elevated FSP1 levels have been associated, among others, with resistance to other therapies and poorer prognosis [445,448]. In recurrent tumors following chemotherapy, increased expression of lipid-metabolism-related genes along with FSP1 has been reported, making this enzyme an attractive therapeutic target [445,448].

**Scheme 35:**
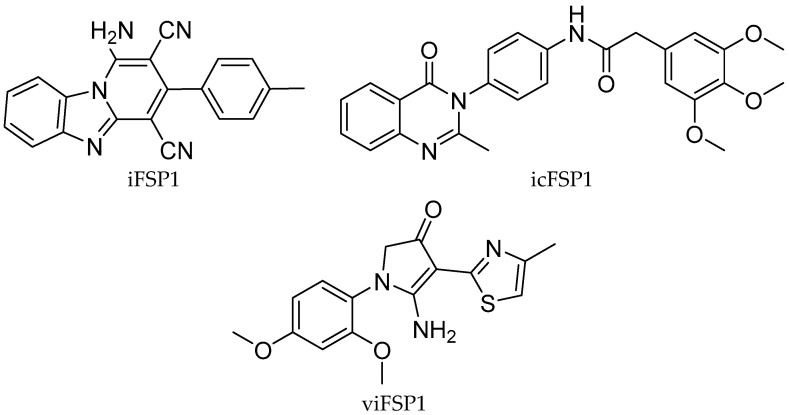
AIFM2/FSP1 inhibitors.

In recent years, specific FSP1 inhibitors have been identified, most notably iFSP1 and the newer **icFSP1**. **iFSP1** (Scheme 35), described as the first small-molecule, competitive FSP1 inhibitor, effectively induces ferroptosis and sensitizes various cancer cell lines to canonical ferroptosis inducers in vitro, and it may also indirectly enhance antitumor immune responses [443,445]. **icFSP1**, a more recent FSP1 inhibitor, acts by inducing FSP1 phase separation and displacing the protein from the membrane, which results in synergistic potentiation of ferroptosis upon concomitant GPX4 inhibition; in mouse models, **icFSP1** suppressed tumor growth without significant systemic toxicity [449]. **viFSP1** is a small-molecule FSP1 inhibitor identified through screening that induces lipid peroxidation and ferroptotic death of FSP1-dependent cells, which can be rescued by the ferroptosis inhibitor liproxstatin-1. In cell-free assays, **viFSP1** inhibits the enzymatic activity of both human and murine FSP1, and its effect is potentiated upon concomitant GPX4 inhibition, indicating synergistic induction of ferroptosis [450].

In parallel, literature reviews emphasize that pharmacological inhibition of FSP1 may represent a strategy to overcome ferroptosis resistance in refractory and metastatic cancers, and the FSP1-dependent CoQ10/VK axis is emerging as one of the most compelling new targets in ferroptosis-inducing therapies [443,444,445]. At present, FSP1/AIFM2 is not a clinically approved drug target, and available inhibitors (**iFSP1**, **icFSP1**, **viFSP1**, and other screening-derived molecules) remain preclinical tools or early drug candidates [444,449]. Nevertheless, accumulating preclinical evidence suggests that FSP1/AIFM2 may ultimately enable a new class of anticancer therapies aimed at pharmacologically unlocking ferroptosis in tumors resistant to other treatment modalities.

### 6.3. Emerging Targets in Immuno-Oncology: CD47-SIRPα, TIGIT, and the STING Pathway

Beyond classical immunotherapy targets, increasing attention has been directed toward a new generation of immune targets for which no approved drugs are yet available; however, some are already being evaluated in advanced clinical stages (e.g., TIGIT), whereas others remain in early development (e.g., STING agonists and the CD47-SIRPα axis) [451,452,453].

#### 6.3.1. The CD47-SIRPα Axis

One of the best-characterized emerging targets is the CD47-SIRPα axis, commonly referred to as the “don’t eat me” signal. CD47 is a surface glycoprotein broadly expressed on both normal and malignant cells; its interaction with SIRPα on macrophages inhibits phagocytosis and promotes tumor immune evasion. Recent reviews highlight that blockade of CD47-SIRPα can restore tumor phagocytosis and enhance antitumor immunity involving both innate and adaptive compartments [453].

The most clinically advanced agent in this class is **magrolimab**, an anti-CD47 antibody evaluated, among others, in combination with azacitidine in previously untreated higher-risk myelodysplastic syndromes. In a phase Ib study, meaningful complete response rates and deep molecular responses were reported in patients with HR-MDS (higher-risk myelodysplastic syndromes), supporting the concept that CD47 blockade can overcome immunologic resistance and potentiate **azacitidine** activity [454]. Another agent targeting this pathway is **evorpacept** (**ALX148**), which blocks the CD47-SIRPα interaction and removes the anti-phagocytic signal; however, because it lacks fragment crystallizable region (Fc) effector function, its antitumor activity is primarily observed in combinations with antibodies such as **rituximab**, which label CD20-positive cells and facilitate macrophage-mediated clearance. In the phase I ASPEN-01 study in patients with relapsed/refractory B-cell non-Hodgkin lymphoma, this combination was well tolerated and showed encouraging efficacy [455].

Development of this drug class is complicated by hematologic toxicity. CD47 serves as a physiological self-marker and is also present on erythrocytes, where it protects them from macrophage phagocytosis; therefore, anti-CD47 agents may cause anemia related to increased erythrocyte clearance, necessitating careful dose selection and close monitoring of blood parameters. Recent reviews emphasize that minimizing on-target toxicity toward circulating erythrocytes remains a major challenge in designing CD47-SIRPα-directed therapies [453,456]. **Maplirpacept** (**TTI-622**, **PF-07901801**), an SIRPα-IgG4 Fc fusion protein engineered to limit red blood cell binding, demonstrated robust preclinical antitumor activity in hematologic malignancy models and enhanced phagocytosis—including in combination settings with clinically used agents—with the potential to reduce erythrocyte-related hematologic toxicity [457]. **Maplirpacept** is currently being evaluated in clinical trials [457].

#### 6.3.2. TIGIT as an Emerging Immune Checkpoint

TIGIT (T cell immunoreceptor with Ig and ITIM domains) is an inhibitory receptor expressed on T lymphocytes, natural killer (NK) cells, and regulatory T cells (Tregs). Its ligands (including CD155) are frequently overexpressed on tumor cells and antigen-presenting cells, which contributes to immunosuppression within the tumor microenvironment and the co-occurrence of dysfunctional/exhausted T-cell features (T-cell exhaustion). Recent reviews classify TIGIT as one of the most important emerging immune checkpoints and a potential target for the next generation of immunotherapies [451,458]. Because TIGIT is very frequently co-expressed with PD-1 on T cells in tumor tissues (most TIGIT+ cells are also PD-1+, and most PD-1+ cells co-express TIGIT) and PD-1 blockade has been shown to compensatorily increase TIGIT expression on the surface of cytotoxic T lymphocytes, concomitant blockade of both receptors may more effectively relieve suppression of the antitumor response than targeting either pathway alone [451,458].

Several anti-TIGIT antibodies are being evaluated in clinical trials. The most advanced include **tiragolumab**, **domvanalimab**, and **vibostolimab**, typically administered in combination with PD-1/PD-L1 inhibitors, while **ociperlimab** and **etigilimab** are also under clinical investigation [459]. In the randomized phase II CITYSCAPE study, tiragolumab plus **atezolizumab** in previously untreated patients with PD-L1-positive non-small-cell lung cancer (NSCLC) showed a higher response rate and longer PFS in selected subgroups compared with **atezolizumab** alone [459,460]. This led to a phase III trial; however, it was discontinued due to failure to meet the primary endpoint [459].

**Domvanalimab** (an anti-TIGIT antibody) was engineered as an Fc-silent antibody lacking Fc effector function to avoid inducing ADCC (antibody-dependent cellular cytotoxicity) and thereby to preserve peripheral Tregs that are critical for maintaining immune homeostasis, which may potentially reduce the risk of autoimmune toxicities compared with Fc-enabled antibodies [461]. **Domvanalimab** in combination with the anti-PD-1 antibody **zimberelimab** showed promising phase II results in the ARC-10 study in NSCLC, and the EDGE-Gastric study (NCT05329766) evaluated **domvanalimab** plus **zimberelimab** with FOLFOX chemotherapy in patients with advanced gastric or esophageal cancer, yielding encouraging efficacy signals, particularly in the subgroup with high PD-L1 expression, without new safety signals. On this basis, a phase III study (NCT05568095) was initiated to compare this combination with a **nivolumab**-based regimen plus chemotherapy in advanced gastric, gastroesophageal junction, and esophageal adenocarcinoma [459,461].

**Vibostolimab** (**MK-7684**), another anti-TIGIT antibody, demonstrated an acceptable safety profile and antitumor activity in selected malignancies in a phase I study in combination with **pembrolizumab** in patients with advanced solid tumors, supporting the biological rationale for TIGIT blockade while also indicating the need for careful patient selection and optimization of combination strategies [459,462]. Despite these encouraging early-phase findings, some large phase III trials with **tiragolumab** did not confirm the expected advantage over standard anti-PD-L1 immunotherapy in the overall NSCLC population, leading to a temporary withdrawal of registration plans in certain indications [463].

TIGIT remains an important candidate target for the next generation of immune checkpoint inhibitors; however, the optimal therapeutic strategy (tumor types, biomarkers, and combinations) is still the subject of intensive clinical investigation [451,459].

#### 6.3.3. The STING Pathway (Stimulator of Interferon Genes)

Another innovative therapeutic target is the STING (stimulator of interferon genes) pathway, which is central to the innate immune response to cytosolic DNA (typically originating from pathogens or damaged host cells). This DNA activates the enzyme cyclic GMP-AMP synthase (cGAS) to generate cyclic GMP, which in turn directly activates the STING receptor located on the endoplasmic reticulum. Activation of the cGAS-STING axis induces the production of type I interferons and pro-inflammatory cytokines (e.g., TNF- α and IL-6), promotes recruitment of effector cells required for immune responses, and can convert the tumor microenvironment toward an inflamed phenotype, making this pathway an attractive target for anticancer therapy [452]. STING agonists are being designed by mimicking natural STING ligands, thereby strengthening immune defense against tumor cells [452].

**Scheme 36:**
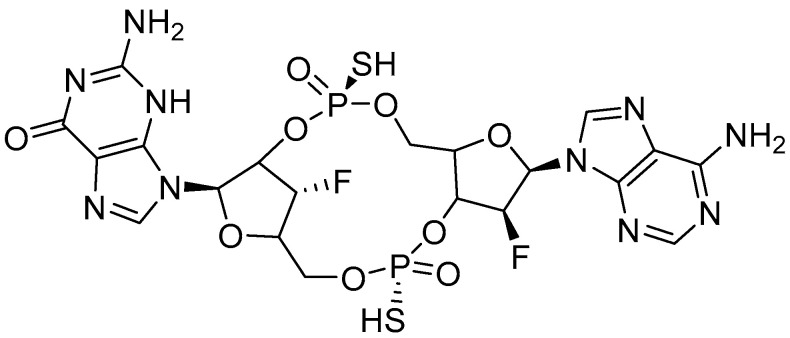
Chemical structure of **MK-1454**.

Early clinical studies of STING agonists include, among others, cyclic dinucleotides (CDNs) that mimic endogenous cyclic dinucleotides, activating the STING pathway, and are administered either locally into the tumor or systemically. **MK-1454** (Scheme 36), a CDN developed by Merck, was evaluated in a phase I/II study (NCT03010176) in which it was administered intratumorally in combination with **pembrolizumab** (anti-PD-1) in patients with advanced solid tumors and lymphomas, which induced type I cytokine production and T-cell infiltration and demonstrated antitumor activity with an acceptable safety profile [452,464].

**Scheme 37:**
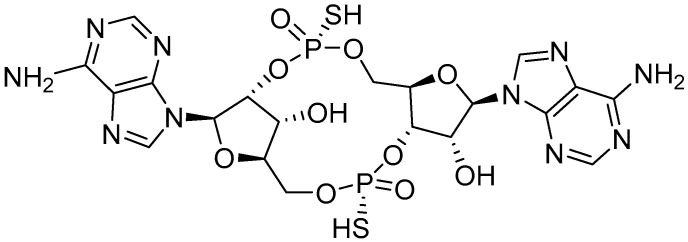
Chemical structure of **MIW815**.

**MIW815** (**ADU-S100)** (Scheme 37), another intratumorally administered STING agonist, has been evaluated in several clinical studies. In a phase I trial in patients with advanced solid tumors and lymphomas, it showed biological activity (induction of interferon-stimulated gene expression and immune infiltration) but relatively limited clinical responses, highlighting the need to optimize dose, route of administration, and combination regimens (e.g., with PD-1 inhibitors) [452,465,466].

Available evidence suggests that although STING agonists can robustly activate innate immunity and modulate the tumor microenvironment, their clinical application is constrained by toxicity (flu-like symptoms and tissue inflammation) and a narrow therapeutic window. In addition to the STING agonists discussed above, other CDN analogs under clinical evaluation include **BMS-986301**, **BI-1387446**, and **TAK-676**, most often tested in combination with other immunotherapies [452]. Current efforts also focus on next-generation STING agonists, such as non-CDN small molecules with unique chemical architectures that enable STING activation, bacterial vectors designed for local generation and release of STING agonists within the tumor, exosome-based CDN delivery systems, and strategies combining these approaches with immune checkpoint inhibitors. The main clinical limitations of STING agonists relate to the risk of excessive systemic inflammation and toxicity (e.g., fever, chills, and CRS (cytokine release syndrome)), particularly with systemic administration, as well as challenges in achieving safe and effective tumor-targeted delivery because intratumoral injections are not feasible for all tumors. Efficacy is further limited by tumor heterogeneity and variable responses (including “cold” tumors and cases with deficient/epigenetically silenced STING signaling), the emergence of resistance, and the need for predictive biomarkers to guide patient selection and optimize combination regimens [452].

### 6.4. Direct Targeting of the MYC Proto-Oncogene

MYC is an oncoprotein that, as a transcription factor and through extensive interactions with other proteins, coordinates gene expression and processes linked to proliferation (including cell-cycle control and DNA replication) and, consequently, influences protein synthesis, genome stability, metabolism, immune responses, and cell survival. MYC remains one of the key regulators of cancer cell proliferation, metabolism, and survival, and is deregulated in a substantial fraction of solid and hematologic malignancies. For many years, MYC was considered largely inaccessible to conventional small-molecule drugs due to its intrinsically disordered protein structure and the lack of a well-defined small-molecule binding pocket. However, a growing body of recent preclinical evidence, together with the first completed phase I studies of direct MYC inhibitors, indicates that both direct and indirect MYC inhibition is a feasible therapeutic strategy [467,468].

#### 6.4.1. Small-Molecule Inhibitors of MYC-MAX

**Scheme 38:**
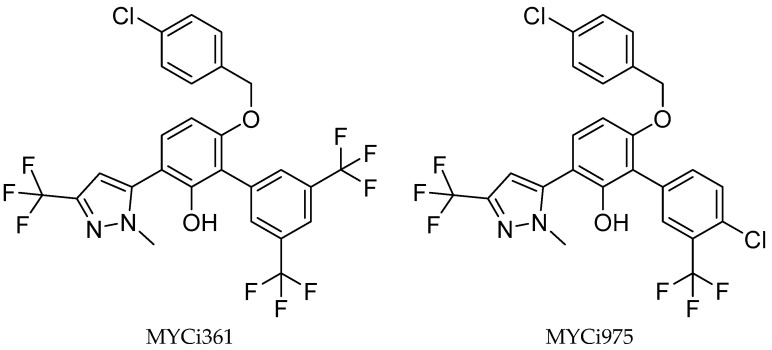
MYC-MAX inhibitors.

A new generation of small-molecule compounds, such as **MYCi361** and its optimized analog **MYCi975** (Scheme 38), has been developed to destabilize the MYC-MAX (MYC Associated Factor X) heterodimer that functions as a transcription factor regulating the expression of genes promoting growth and proliferation [469]. **MYCi361** was evaluated in preclinical models, where it increased immune-cell infiltration and suppressed tumor growth [470]. Compared with **MYCi361**, which showed limited tolerability, **MYCi975** demonstrated a more favorable safety profile while maintaining immunomodulatory activity [469]. **MYCi975** has been shown to bind MYC, disrupt MYC-MAX dimerization, enhance proteasome-mediated MYC degradation, and inhibit tumor growth in in vivo models with acceptable tolerability [468]. The same compound selectively remodels MYC and MAX DNA-binding profiles and histone H3K27 acetylation (H3K27ac), leading to repression of key proliferative genes, as well as sensitization of prostate cancer cells to enzalutamide and ER-positive breast cancer cells to 4-hydroxytamoxifen. Thus, **MYCi975** attenuates MYC-dependent signaling and provides a rationale for exploring combination treatment strategies [468].

#### 6.4.2. The Omomyc Mini-Protein (OMO-103) as the First Clinical MYC Inhibitor

The most clinically advanced approach to direct MYC inhibition is the **Omomyc mini-protein** (**OMO-103**). Omomyc is a MYC-derived variant that competes for MAX and DNA binding, forming non-productive dimers and thereby blocking endogenous MYC function, while retaining the ability to penetrate cells and translocate to the nucleus [467]. In a recently published phase I study in patients with advanced solid tumors, once-weekly **OMO-103** administration was well tolerated; approximately half of the evaluable patients achieved stable disease and showed early signals of antitumor activity, including measurable tumor shrinkage. In one patient with metastatic pancreatic cancer, a 49% reduction in total tumor burden was observed, and tumor biopsies confirmed suppression of MYC target genes, providing the first clinical proof of concept for direct MYC inhibition in humans [467,471]. These findings indicate that direct targeting of MYC by modulating protein–protein interactions and disrupting MYC-MAX dimers is becoming a feasible, albeit still early, therapeutic strategy, and its success could pave the way for a new class of drugs directed against transcription factors that have long been considered highly challenging to inhibit pharmacologically [467,472].

#### 6.4.3. MYC Degradation Strategies: PROTACs

**Scheme 39:**
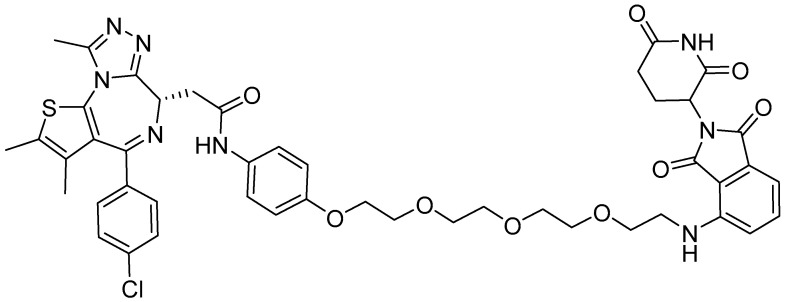
Chemical structure of **ARV-825**.

Another important direction comprises strategies based on the selective elimination of regulators within the MYC axis via the ubiquitin-proteasome system. In 2020, Saraswat et al. reported a nanoformulation of the BRD4-degrading PROTAC **ARV-825** (Scheme 39), which indirectly reduces MYC expression in pancreatic cancer. The use of a nanocarrier markedly improved compound stability and enhanced BRD4 degradation with consequent MYC suppression, resulting in pronounced cytotoxicity, apoptosis, and inhibition of growth of 3D pancreatic cancer tumor spheroids [473]. **ARV-825** was also evaluated in a SCID mouse xenograft model using SU-DHL-4 cells, where it was more effective than **JQ1** in inhibiting DLBCL cell proliferation, inducing apoptosis, promoting cell-cycle arrest, and prolonging survival. Compared with **JQ1**, **ARV-825** more strongly reduced c-MYC and BET-family protein levels in both in vitro and in vivo settings. These findings suggest that BET-targeting PROTACs may represent a promising therapeutic strategy in DLBCL [474].

**Scheme 40:**
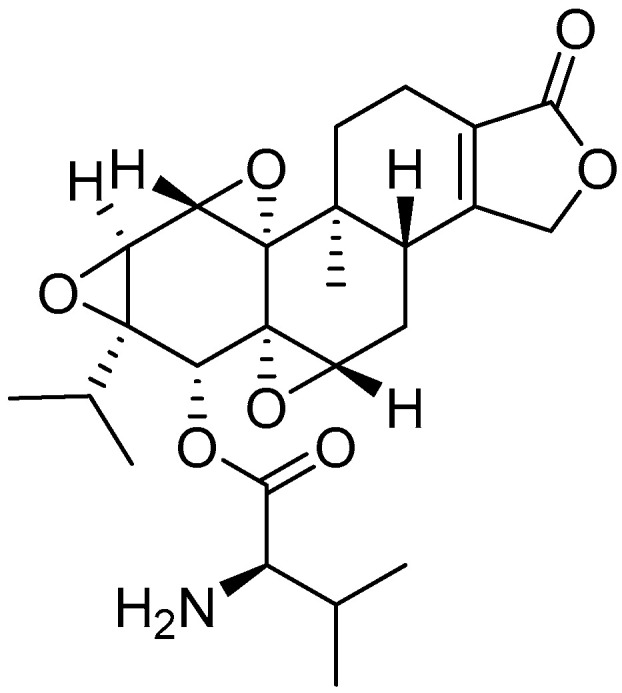
Chemical structure of **WBC100**.

Recent reviews emphasize that PROTACs and other degraders are also being developed to act more directly against MYC, including molecules such as **WBC100** (Scheme 40), which in preclinical models induce selective MYC degradation and tumor regression, although they remain at an early stage of development [467,472]. **WBC100** is an orally administered MYC degrader based on PROTAC technology, designed to promote targeted MYC protein degradation; in a phase I clinical study (NCT05100251) in patients with advanced solid tumors, it demonstrated a tolerable safety profile. Among evaluable patients, preliminary antitumor activity was observed (a single partial response and cases of stable disease), with a particularly notable signal in PDAC [475].

Collectively, evidence from recent years indicates that MYC is becoming a feasible target for several classes of innovative therapeutics. Although none of these agents has been approved to date, results from preclinical studies and early-phase clinical trials suggest that MYC may, in the coming years, emerge as a therapeutic vulnerability across a broader range of anticancer drugs, particularly in tumors that are highly dependent on this oncogene.

### 6.5. Summary

The molecular targets presented in this section share several common features. First, in most cases, these are proteins or complexes that, until recently, were considered very difficult to inhibit pharmacologically; this applies to the MDM2/MDM4 ligase regulating p53 stability, mutant forms of p53 itself, the MYC proto-oncogene, and FSP1 (AIFM2), which protects cells against ferroptosis. Second, the therapeutic strategies discussed are no longer limited to the classical inhibition of a single kinase, but instead intervene at key regulatory nodes of cancer cells, including restoration of the genome guardian function (p53), abrogation of protection from ferroptosis (FSP1), modulation of immune responses (CD47-SIRPα, TIGIT, STING), or direct attenuation of a transcriptional oncogene (MYC).

Currently, the available clinical evidence remains limited and is largely derived from preclinical studies and early-phase trials in small patient cohorts. Further development of these agents will depend on more comprehensive toxicity profiling, improved biomarker identification, and optimal integration with established targeted therapies and immunotherapy. Nevertheless, the overall direction is clear: the next generation of targeted therapies will likely shift from simple kinase inhibitors toward agents that modulate protein–protein interactions, specific modes of cell death, and more complex mechanisms of immune regulation. If the current promising results are confirmed in larger studies, the targets described here may provide the foundation for new drug classes that complement and expand the therapeutic options currently available in oncology.

## 7. Discussion and Future Perspectives

Recent advances in targeted cancer therapy confirm a decisive transition from the historical one-size-fits-all cytotoxic paradigm toward precision modulation of tumor-specific molecular vulnerabilities. Over the analyzed period, both small-molecule agents and biologics have undergone rapid evolution, driven by expanding knowledge of cancer genomics, high-resolution molecular profiling, and increasingly sophisticated drug design strategies. Importantly, progress is no longer defined solely by the identification of single inhibitory molecules, but by the development of coherent, biology-driven therapeutic strategies that integrate structural, functional, and systems-level insights. Contemporary targeted therapy extends beyond single-pathway blockade and increasingly aims at dynamic modulation of interconnected signaling networks. Across major target classes—including receptor and non-receptor kinases, components of the PI3K/AKT/mTOR axis, antiapoptotic BCL-2 family proteins, PARP enzymes, regulators of epigenetic architecture, and DNA damage response pathways—several unifying mechanistic and medicinal chemistry principles emerge.

First, kinase inhibitor design continues to rely heavily on privileged heterocyclic scaffolds such as pyrimidines, quinazolines, and azolopyrimidines, which provide robust hinge-binding interactions and synthetic versatility. However, the clinical emergence of resistance-associated mutations, including gatekeeper and solvent-front substitutions, necessitates strategies that move beyond classical ATP-competitive paradigms. Conformationally adaptive inhibitors, covalent and reversible covalent agents with optimized warhead positioning, and allosteric modulators targeting non-conserved regulatory pockets are increasingly central to overcoming acquired resistance and reducing off-target cross-reactivity.

Second, targeting antiapoptotic proteins such as BCL-2 and MCL-1 exemplifies the shift from catalytic site inhibition to topology-driven engagement of protein–protein interfaces. BH3 mimetics demonstrate that faithful reproduction of α-helical interaction geometry and precise engagement of hotspot residues are critical for isoform selectivity and therapeutic window optimization. Future refinement in this domain will likely involve macrocyclization strategies, transient exposure regimens (pulse dosing), and rational combination approaches designed to mitigate mechanism-based toxicities.

Third, PARP inhibitors illustrate how subtle structural modulation within the NAD^+^-binding pocket influences not only catalytic inhibition but also PARP trapping efficiency—an increasingly recognized determinant of therapeutic index. Fine-tuning trapping potency, exploiting replication stress, and combining with DNA damage response modulators represent promising avenues for improving the durability of response.

At the same time, biologic, immunomodulatory, and cell-based therapies are gaining prominence, complementing small-molecule inhibitors and expanding therapeutic opportunities in malignancies previously considered refractory. The current therapeutic landscape—encompassing newly approved small molecules, biologics, and agents in preclinical and clinical development—reflects the breadth and diversification of modern molecular oncology. The growing number of emerging targets suggests that the coming years will further broaden the spectrum of actionable vulnerabilities.

Looking forward, several research directions appear particularly consequential:

- Expansion of allosteric and non-ATP site inhibitors to circumvent conserved catalytic constraints and resistance mutations.
- Optimization of covalent and reversible covalent chemotypes to balance durability of target engagement with safety.
- Development of targeted protein degradation strategies (e.g., PROTACs and molecular glues), particularly for kinases and antiapoptotic proteins that escape occupancy-driven inhibition.
- Integration of structural biology with molecular dynamics simulations to better anticipate conformational plasticity and resistance evolution.
- Network-informed combination design based on signaling topology and adaptive rewiring, rather than empirical synergy screening alone.
- Continued advancement of chemotypes capable of selectively modulating extended or shallow protein–protein interfaces.

Collectively, targeted therapy remains one of the most rapidly advancing domains in oncology. Its future will depend on the integration of structural biology, synthetic chemistry, tumor genomics, and systems-level signaling analysis. The transition from static enzyme inhibition to dynamic, resistance-aware modulation of signaling architecture represents a defining shift in modern anticancer drug discovery. A deep understanding of molecular mechanisms—coupled with scaffold innovation and rational combination strategies—will be essential for delivering more effective, safer, and increasingly personalized therapeutic approaches.

## Figures and Tables

**Table 2:** Summary of selected FDA-approved small-molecule targeted therapies from 2022 to 2025 (Sources: Polish Society of Oncology; www.fda.gov).

Class	Molecular Target	Drug Name	Clinical Indication	FDA Approval Date	Pivotal Trial
TKI—EGFR	EGFR exon 20 insertion mutation	Sunvozertinib (Zegfrovy)	Locally advanced or metastatic NSCLC with an EGFR exon 20 insertion mutation after ≥1 prior EGFR-targeted therapy	2 July 2025	WU-KONG-1B (NSCLC EGFR ex20ins) [336,337,338]
	EGFR with activating mutations (exon 19 deletion, L858R)	Osimertinib (Tagrisso)	Locally advanced, unresectable stage II EGFR-positive NSCLC after chemoradiotherapy	25 September 2024	LAURA [339]
Combination: EGFR TKI (third generation) + bispecific IgG targeting EGFR/MET	EGFR-mutant NSCLC; synergistic inhibition of EGFR and MET	Amivantamab + lazertynib	First-line treatment of patients with locally advanced or metastatic EGFR-mutant NSCLC	19 August 2024	MARIPOSA [340,341,342]
TKI—ALK	ALK	Ensartinib (Ensacove)	ALK-positive NSCLC (treatment of patients with locally advanced or metastatic ALK-positive NSCLC after prior ALK inhibitor therapy)	18 December 2024	eXALT-3 [343,344]
	ALK	Alectinib (Alecensa)	Adjuvant treatment of ALK-positive NSCLC after resection	18 April 2024	ALINA [345,346,347]
TKI—ROS1	ROS1 (rearranged/fusion tyrosine kinase)	Taletrectinib (Ibtrozi)	Locally advanced or metastatic ROS1-positive NSCLC (treatment-naive or previously treated)	11 June 2025	TRUST-I, TRUST-II [348,349]
	ROS1/NTRK (TRK)—inhibitor of ROS1 and NTRK fusion kinases	Repotrectinib (Augtyro)	Locally advanced or metastatic ROS1-positive NSCLC (2023) and NTRK-positive solid tumors (label expansion, 2024)	15 November 2023 (NSCLC ROS1)/13.06.2024 (NTRK-positive solid tumors)	TRIDENT-1—phase I/II trial of repotrectinib in patients with ROS1-positive NSCLC and NTRK-positive solid tumors [350]
TKI—MET	MET	Tepotinib (Tepmetko)	Metastatic NSCLC with a MET exon 14 skipping mutation	15 February 2024	VISION—updated indication for tepotinib (Tepmetko) in MET exon 14 skipping NSCLC [351,352]
	MET	Capmatinib (Tabrecta)	Treatment of metastatic NSCLC with a MET exon 14 skipping mutation (full approval; expansion of the 2020 indication)	10 August 2022	GEOMETRY mono-1 [353,354]
TKI—cMET-targeted ADC	MET-high NSCLC (c-MET ≥ 50%)	Telisotuzumab vedotin-tllv (Emrelis)	Treatment of locally advanced or metastatic non-squamous NSCLC with high MET expression (≥50%) after prior therapy	14 May 2025	LUMINOSITY [355,356,357]
TKI—RET	RET (RET fusions in NSCLC; RET mutations in MTC and thyroid cancer)	Selpercatinib (Retevmo)	2022–2024: subsequent label expansions—including advanced/metastatic RET-positive MTC and RET-positive thyroid cancer/NSCLC in adults and pediatric patients aged ≥2 years	21 September 2022; 12 June 2024; 27 September 2024	LIBRETTO-001 [358,359,360]/ LIBRETTO-531 [361]/ LIBRETTO-121 [362,363]
	RET (RET fusions in NSCLC)	Pralsetinib (Gavreto)	Treatment of metastatic RET fusion-positive NSCLC	9 August 2023 (full approval)	ARROW [364,365,366]
TKI—FGFR	FGFR3 (inhibitor FGFR2/3)	Erdafitynib (Balversa)	Locally advanced or metastatic UC with an FGFR3 mutation (label expansion)	19 January 2024	BLC3001—randomized trial of erdafitinib versus chemotherapy in patients with advanced UC with an FGFR3 mutation [367,368]
	FGFR2 (tyrosine kinase inhibitor of FGFR1–4, predominantly FGFR2)	Futibatinib (Lytgobi)	FGFR2-positive biliary tract cancer (unresectable or metastatic intrahepatic CCA with an FGFR2 rearrangement after prior therapy)	30 September 2022	FOENIX-CCA2—single-arm trial of futibatinib in patients with intrahepatic CCA with an FGFR2 rearrangement [369]
	FGFR1 (FGFR1–3 kinase inhibitor; key target FGFR1 in MLN-FGFR1)	Pemigatinib (Pemazyre)	Relapsed or refractory myeloid/lymphoid neoplasms with FGFR1 rearrangement (MLN-FGFR1)	26 August 2022	FIGHT-203—trial of pemigatinib in myeloid/lymphoid neoplasms with FGFR1 rearrangement [370]
TKI-FLT3	FLT3-ITD (selective FLT3 inhibitor)	Quizartinib (Vanflyta)	Newly diagnosed AML with an FLT3-ITD mutation in adults (in combination with induction and consolidation chemotherapy: cytarabine plus an anthracycline for induction, and cytarabine for consolidation)	20 July 2023	QuANTUM-First—randomized trial of quizartinib plus standard chemotherapy versus placebo in patients with FLT3-ITD-mutated AML [371]
TKI—KRAS	KRAS G12C mutation plus anti-EGFR antibody	Sotorasib + panitumumab	Treatment of patients with metastatic KRAS G12C-positive colorectal cancer after prior fluoropyrimidine-, oxaliplatin-, and irinotecan-based therapy	16 January 2025	CodeBreaK 300 [372,373]
	KRAS G12C (covalent KRAS G12C inhibitor)	Adagrasib + cetuksymab (Krazati)	Locally advanced or metastatic KRAS G12C-positive colorectal cancer after prior therapy (in combination with cetuximab)	21 June 2024	KRYSTAL-1—cohort of patients with KRAS G12C-mutant colorectal cancer treated with adagrasib plus cetuximab [374]
TKI targeting c-KIT/PDGFRA (GIST-like); CSF1R kinase inhibitor	CSF1R on macrophage-lineage cells (drives recruitment and survival of cells in TGCT)	Vimseltinib (Romvimza)	Treatment of patients with symptomatic tenosynovial giant cell tumor (TGCT) for whom surgery is not feasible	14 February 2025	MOTION [375,376,377]
TKI—PI3K/AKT/mTOR	PIK3CA gain-of-function—PROS (PIK3CA-related overgrowth spectrum)	Alpelisib (Vijoice)	Treatment of pediatric patients aged ≥2 years and adults with severe manifestations of PROS	6 April 2022	EPIK-P1 [378,379]
	AKT1/PIK3CA/PTEN-altered PI3K/AKT pathway in HR-positive/HER2-negative breast cancer	Capivasertib (Truqap) + fulvestrant	HR-positive, HER2-negative advanced/metastatic breast cancer with PIK3CA or AKT1 mutations or PTEN loss, in combination with fulvestrant after prior endocrine therapy and a CDK4/6 inhibitor	16 November 2023	CAPItello-291 (capivasertib + fulvestrant vs. placebo + fulvestrant) [380]
	PI3Kα (PIK3CA)	Inavolisib (Itovebi)	HR-positive, HER2-negative, PIK3CA-mutant advanced/metastatic breast cancer—in combination with palbociclib and fulvestrant after prior endocrine therapy	10 October 2024	INAVO120 (inavolisib + palbocyklib + fulvestrant vs. placebo) [381,382]
BRAF/MEK pathway	BRAF V600E	Toworafenib (Ojmeda)	Treatment of pediatric patients aged ≥6 months with BRAF-positive low-grade glioma (LGG)	23 April 2024 Under accelerated approval	FIREFLY [383,384,385]
	BRAF V600E	Dabrafenib + Trametinib (Mekinist, Novartis)	Treatment of pediatric patients aged ≥1 year with BRAF V600E-positive LGG	16 March 2023	CDRB436G2201 [386,387]
NRTK inhibitor (FAK/PTK2) + MAPK pathway inhibitor (RAF/MEK)	FAK/KRAS	Avutometinib + defactinib	Treatment of adults with KRAS-mutant low-grade serous ovarian carcinoma (LGSOC)	8 May 2025	RAMP 301 [11]
DNA repair proteins PARP1/2	PARP1/2 (inhibitor PARP)	Niraparib (component of Akeega—niraparib plus abiraterone) in combination with prednisone	mCRPC with BRCA1/2 mutations, in combination with abiraterone acetate and prednisone	11 August 2023	MAGNITUDE [388,389,390]
	PARP1/2 (inhibitor PARP)	Olaparib (Lynparza)	High-risk early-stage BRCA1/2-mutated, HER2-negative breast cancer after systemic therapy (adjuvant targeted therapy)	11 March 2022	OlympiA [391,392]
	PARP1/2 (PARP inhibitor; synthetic lethality in tumors with HRR defects)	Talazoparib (Talzenna)	mCRPC with HRR gene alterations, in combination with enzalutamide	20 June 2023	TALAPRO-2 [393]
Epigenetic inhibitors	IDH1/IDH2 (dual inhibitor of mutant IDH1 and IDH2 enzymes)	Vorasidenib (Voranigo)	WHO grade 2 astrocytoma or oligodendroglioma with an IDH1 or IDH2 mutation after resection or biopsy (LGG)	6 August 2024	INDIGO [394]
	IDH1 (selective inhibitor of mutant IDH1)	Olutasidenib (Rezlidhia)	Relapsed/refractory AML with an IDH1 mutation in adults	1 December 2022	2102-HEM-101 [266,395]
OTHER					
HIF pathway (hypoxia-inducible factor)	HIF-2α (selective inhibitor of the HIF-2α-ARNT complex)	Belzutifan (Welireg)	Advanced renal cell carcinoma (mRCC) after prior PD-1/PD-L1 and VEGF inhibitor therapy (2023) and adrenal pheochromocytoma/paraganglioma in adults and pediatric patients aged ≥12 years with locally advanced, unresectable, or metastatic disease (2025)	14 December 2023 (mRCC)/14 May 2025 (pheo/paraganglioma)	LITESPARK-005 [396] LITESPARK-015 [397]
Estrogen receptor (ER)	ER—selective estrogen receptor degrader (SERD)	Imlunestrant/Inluriyo	Advanced/metastatic ER-positive, HER2-negative, ESR1-mutant breast cancer after ≥1 line of endocrine therapy	25 September 2025	EMBER-3 [398,399]
NOTCH	γ-secretase (gamma-secretase inhibitor; blocks NOTCH signaling)	Nirogacestat (Ogsiveo)	Progressive desmoid tumor requiring systemic therapy	27 November 2023	DeFi [400,401]

## Data Availability

No new data were created or analyzed in this study. Data sharing is not applicable to this article.

## Supplementary Materials

The following supporting information can be downloaded at: https://www.mdpi.com/article/10.3390/molecules31071195/s1. Table S1: Representative approved TKIs used in targeted therapy; Table S2: Summary of selected small-molecule targeted drugs approved by the FDA in 2022–2025; Table S3: Summary of selected targeted biologic drugs approved by the FDA in 2022–2025 [415,416,426,427,428,476,477,478,479,480,481,482,483,484,485,486,487,488,489,490,491,492,493,494,495,496,497,498,499,500,501,502,503,504,505,506,507,508,509,510,511,512,513,514,515,516,517,518,519,520].

## Abbreviations

The following abbreviations are used in this manuscript:

^177^Lu-PSMA-617	lutetium-177- prostate-specific membrane antigen-617 radioligand
2-HG	D-2-hydroxyglutarate
ABCB1	ATP-binding cassette subfamily B member 1
ACC	adenoid cystic carcinoma
AD	adenine-ribose binding site (adenine site)
ADCC	antibody-dependent cellular cytotoxicity
ADC	antibody–drug conjugate
ADME	absorption, distribution, metabolism, and excretion
AIFM2	apoptosis-inducing factor mitochondria-associated 2 (also known as FSP1)
AKT	protein kinase B (PKB)
ALK	anaplastic lymphoma kinase
ALL	acute lymphoblastic leukemia
AML	acute myeloid leukemia
ARAF	A-Raf proto-oncogene, serine/threonine kinase
ARNT	aryl hydrocarbon receptor nuclear translocator
ASCO	American Society of Clinical Oncology
ATR	ataxia telangiectasia and Rad3-related protein kinase
ATP	adenosine triphosphate
BAD	BCL2-associated agonist of cell death
BAK	BCL2 antagonist/killer 1
BAX	BCL2-associated X, apoptosis regulator
BCMA	B-cell maturation antigen
BCL-2	B-cell lymphoma 2
BCL-W	BCL2-like 2 (BCL2L2)
BCL-XL	B-cell lymphoma-extra large (BCL2L1)
BCR	B-cell receptor
BCR-ABL/BCR::ABL1	BCR-ABL fusion tyrosine kinase
BD1/BD2	bromodomain 1/2
BET	bromodomain and extra-terminal (proteins)
BH3	BCL-2 homology 3
BIK	BCL2-interacting killer (NBK)
BIM	BCL2-like 11 (BCL2L11)
BMF	BCL2-modifying factor
BRAF	B-Raf proto-oncogene, serine/threonine kinase
BRAFi	BRAF inhibitor
BRCA1/2	breast cancer gene 1/2 (DNA repair-associated)
BRD2/3/4	bromodomain-containing protein 2/3/4
BsAb	bispecific antibody
BTK	Bruton tyrosine kinase
cGAS	cyclic GMP-AMP synthase
CAR-T	chimeric antigen receptor T-cell therapy
CCA	cholangiocarcinoma
CD	cluster of differentiation
CDN	cyclic dinucleotide
CDK	cyclin-dependent kinase
ChK1	checkpoint kinase 1
CLL	chronic lymphocytic leukemia
CML	chronic myeloid leukemia
CoQ10	coenzyme Q10 (ubiquinone)
CRC	colorectal cancer
CRS	cytokine release syndrome
CTCL	cutaneous T-cell lymphoma
CTLA-4	cytotoxic T-lymphocyte-associated protein 4
ctDNA	circulating tumor DNA
DDLPS	dedifferentiated liposarcoma
DDR	DNA damage response
DLBCL	diffuse large B-cell lymphoma
DLT	dose-limiting toxicity
DM1	mertansine
DNA	deoxyribonucleic acid
DSB	double-strand break
DTC	differentiated thyroid carcinoma
E3	E3 ubiquitin ligase
EGFR	epidermal growth factor receptor
EGFR-TKI	epidermal growth factor receptor tyrosine kinase inhibitor
EML4-ALK	EML4-ALK fusion (echinoderm microtubule-associated protein-like 4-ALK)
EMT	epithelial–mesenchymal transition
EP300/p300	E1A binding protein p300 (histone acetyltransferase coactivator)
ER	estrogen receptor
ERK	extracellular signal-regulated kinase
ESR1	estrogen receptor 1 (gene)
FAK	focal adhesion kinase
FAO	fatty acid oxidation
Fc	fragment crystallizable region
FDA	U.S. Food and Drug Administration
FGF	fibroblast growth factor
FGFR	fibroblast growth factor receptor
FKBP12	FK506-binding protein 12
FL	follicular lymphoma
FLT3-ITD	fms-like tyrosine kinase 3 internal tandem duplication
FOLFOX	folinic acid (leucovorin), fluorouracil, and oxaliplatin
FRα	folate receptor alpha
FRB	FKBP12-rapamycin binding domain
FSP1	ferroptosis suppressor protein 1 (also known as AIFM2)
GDNF	glial cell line-derived neurotrophic factor
GIST	gastrointestinal stromal tumor
GPX4	glutathione peroxidase 4
gp100	glycoprotein 100 (PMEL)
GTP	guanosine triphosphate
H3K27ac	histone H3 lysine 27 acetylation
HCC	hepatocellular carcinoma
HDAC	histone deacetylase
HDACi	histone deacetylase inhibitor(s)
HER2	human epidermal growth factor receptor 2 (ERBB2)
HER3	human epidermal growth factor receptor 3 (ERBB3)
HGF	hepatocyte growth factor
HIF	hypoxia-inducible factor
HNSCC	head and neck squamous cell carcinoma
HR	homologous recombination; hormone receptor (context dependent)
HR+	hormone receptor-positive
HRD	homologous recombination deficiency
HRR	homologous recombination repair
HR-MDS	higher-risk myelodysplastic syndromes
IC_50_	half maximal inhibitory concentration
IDH1/2	isocitrate dehydrogenase 1/2
IDH-DS	IDH differentiation syndrome
IGF-1R	insulin-like growth factor 1 receptor
IgG	immunoglobulin G
IL-2/IL-6	interleukin 2/interleukin 6
IRS-1	insulin receptor substrate 1
JAK	Janus kinase
JAK/STAT	Janus kinase/signal transducer and activator of transcription
KAc	lysine acetylation
KIT	KIT proto-oncogene, receptor tyrosine kinase
KRAS	(Kirsten Rat Sarcoma Viral Oncogene Homolog) proto-oncogene, GTPase
KRAS G12C	KRAS G12C mutation
KRASG12D	KRAS G12D mutation
L858R	EGFR leucine-to-arginine substitution at codon 858
LGG	low-grade glioma
Lig3	DNA ligase III
LGSOC	low-grade serous ovarian carcinoma
MAPK	mitogen-activated protein kinase
MAX	MYC-associated factor X
MCL	mantle cell lymphoma
MCL-1	myeloid cell leukemia 1
mCRPC	metastatic castration-resistant prostate cancer
MDM2	mouse double minute 2 homolog (E3 ubiquitin ligase)
MDM4/MDMX	mouse double minute 4 homolog
MDR1	multidrug resistance protein 1
MDS	myelodysplastic syndrome(s)
MEK	mitogen-activated protein kinase kinase (MAP2K)
MEKi	MEK inhibitor
MET	mesenchymal–epithelial transition factor (receptor tyrosine kinase)
METex14	MET exon 14 skipping alteration
MHC-II	major histocompatibility complex class II
MLN-FGFR1	myeloid/lymphoid neoplasm with FGFR1 rearrangement
MMAE	monomethyl auristatin E
MPNST	malignant peripheral nerve sheath tumor
mRCC	metastatic renal cell carcinoma
MRTX1133	KRASG12D-selective inhibitor MRTX1133
mTOR	mechanistic target of rapamycin
mTORC1/2	mechanistic target of rapamycin complex 1/2
MTC	medullary thyroid carcinoma
MYC/c-MYC	MYC proto-oncogene (transcription factor)
MYC-MAX	MYC-MAX heterodimeric transcriptional complex
MZL	marginal zone lymphoma
NAD+	nicotinamide adenine dinucleotide (oxidized form)
NADPH	reduced nicotinamide adenine dinucleotide phosphate
NCT	ClinicalTrials.gov identifier (prefix)
NF-κB	nuclear factor kappa B
NHEJ	non-homologous end joining
NHL	non-Hodgkin lymphoma
NI	nicotinamide-ribose binding site (nicotinamide site)
NK	natural killer (cell)
NOTCH	Notch signaling pathway
NOXA	phorbol-12-myristate-13-acetate-induced protein 1 (PMAIP1); a pro-apoptotic BH3-only protein (BCL-2 family)
NRTK	non-receptor tyrosine kinase
NSCLC	non-small cell lung cancer
NTRK	neurotrophic tyrosine receptor kinase
OXPHOS	oxidative phosphorylation
P-TEFb	positive transcription elongation factor b
PALB2	partner and localizer of BRCA2
PAR	poly(ADP-ribose)
PARG	poly(ADP-ribose) glycohydrolase
PARP	poly(ADP-ribose) polymerase
PARPi	PARP inhibitor(s)
PD-1	programmed cell death protein 1
PD-L1	programmed death-ligand 1
PDAC	pancreatic ductal adenocarcinoma
PDGFRA	platelet-derived growth factor receptor alpha
PDTO	patient-derived tumor organoids
PBD	pyrrolobenzodiazepine
PFS	progression-free survival
PH	phosphate binding site
PI3K	phosphoinositide 3-kinase
PIK3CA	phosphatidylinositol-4,5-bisphosphate 3-kinase catalytic subunit alpha
P-gp	P-glycoprotein
pNET	pancreatic neuroendocrine tumor(s)
Polβ	DNA polymerase beta
PPI	protein–protein interaction
PROS	PIK3CA-related overgrowth spectrum
PROTAC	proteolysis-targeting chimera
PSC	pulmonary sarcomatoid carcinoma
PSMA	prostate-specific membrane antigen
PTEN	phosphatase and tensin homolog
PUMA	p53 upregulated modulator of apoptosis (BBC3)
RAF	RAF kinase family (ARAF, BRAF, RAF1/CRAF)
RAF1	RAF proto-oncogene serine/threonine-protein kinase 1
RAS	RAS family small GTPases
RAS (ON)	active, GTP-bound RAS state
RCC	renal cell carcinoma
RET	rearranged during transfection proto-oncogene (receptor tyrosine kinase)
RNA Pol II	RNA polymerase II
ROS	reactive oxygen species
ROS1	ROS proto-oncogene 1 (receptor tyrosine kinase)
RTK	receptor tyrosine kinase
SAR	structure–activity relationship
SCLC	small cell lung cancer
SCID	severe combined immunodeficiency
SD	stable disease
SD36	STAT3 degrader SD36
SERD	selective estrogen receptor degrader
SIRPα	signal regulatory protein alpha
SLL	small lymphocytic lymphoma
SNAr	nucleophilic aromatic substitution
SPP1	secreted phosphoprotein 1 (osteopontin)
SSB	single-strand break
STAT	signal transducer and activator of transcription
STING	stimulator of interferon genes
STS	soft tissue sarcoma
SU-DHL-4	human DLBCL cell line
TAD	transactivation domain
TGCT	tenosynovial giant cell tumor
TIGIT	T cell immunoreceptor with Ig and ITIM domains
TIL	tumor-infiltrating lymphocyte therapy
TK	tyrosine kinase
TKI	tyrosine kinase inhibitor(s)
TNF-α	tumor necrosis factor alpha
TP53	tumor protein p53 (gene)
TRK	tropomyosin receptor kinase
Treg	regulatory T cell
TROP2	trophoblast cell surface antigen 2
TYK2	tyrosine kinase 2
UC	urothelial carcinoma
VEGF	vascular endothelial growth factor
VEGFR	vascular endothelial growth factor receptor
VHL	von Hippel-Lindau (E3 ligase used in PROTAC design)
VK	vitamin K
WEE1	WEE1 G2 checkpoint kinase
WGR	WGR domain (PARP DNA-binding related domain)
WHO	World Health Organization
WM	Waldenstrom macroglobulinemia
WT	wild-type
XRCC1	X-ray repair cross-complementing protein 1
Y220C	TP53 missense mutation (tyrosine-to-cysteine substitution at residue 220)
α-KG	alpha-ketoglutarate
