# Supplementary material for: Molecularly Targeted Therapies in Oncology: Mechanisms, Resistance, and Combination Strategies
Source: Molecules. 2026 Apr 3;31(7):1195. doi: 10.3390/molecules31071195 (PMC13074999; doi:10.3390/molecules31071195)
Supplement: Supplementary file 1 [file molecules-31-01195-s001.zip › molecules-4207593-supplementary.pdf]

Supplementary Materials

Molecularly Targeted Therapies in Oncology: Mechanisms, Resistance, and Combination Strategies

Klaudia Giercuszkiewicz-Haśnik [1,2,3*], Beata Morak-Młodawska [4], Małgorzata Jeleń [4*]

[1] Department of Systems Biology and Engineering, Silesian University of Technology, Gliwice 44−100, Poland;

[2] Centre of Biotechnology, Silesian University of Technology, Gliwice 44-100, Poland; klaudia.giercuszkiewicz@polsl.pl (K.G.-H.);

[3] Faculty of Medical Sciences in Katowice, Medical University of Silesia, Katowice 40-752, Poland

[4] Department of Organic Chemistry, Faculty of Pharmaceutical Sciences in Sosnowiec, Medical University of Silesia in Katowice, Sosnowiec 41-200, Poland; manowak@sum.edu.pl (M.J), bmlodawska@sum.edu.pl (B.M.-M.)

[*] Correspondence: klaudia.giercuszkiewicz@polsl.pl; manowak@sum.edu.pl

Table S1 Representative approved TKI inhibitors used in targeted therapy. Compound structures were retrieved from the ChEMBL database and then visualized and formatted for the manuscript.

Representative approved TKI inhibitors	Structures
Acalabrutinib	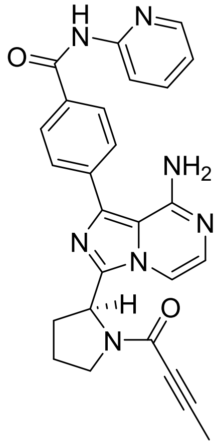
Afatinib	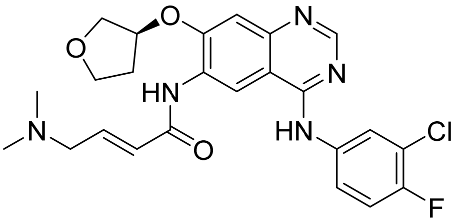

Axitinib	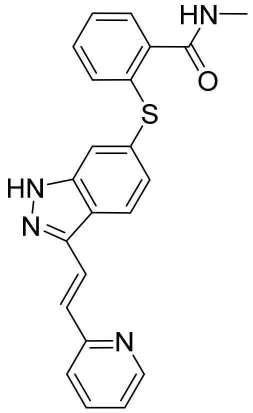
Alectinib	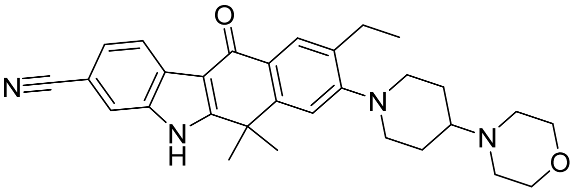
Anlotinib	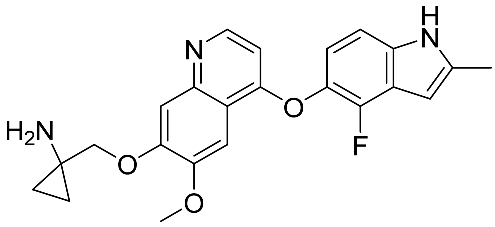
Apatinib	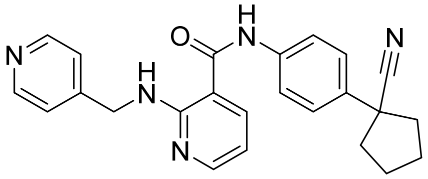
Asciminib	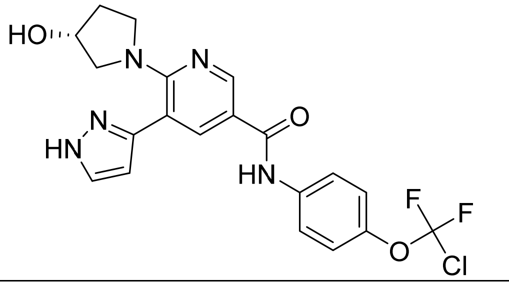
Avapritinib	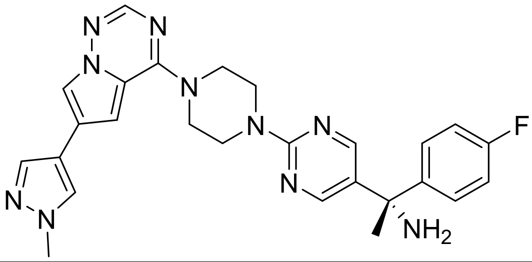
Bosutinib	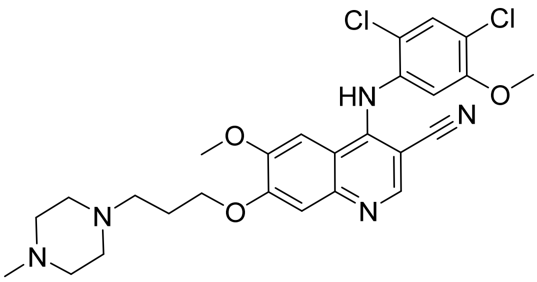

Brigatinib	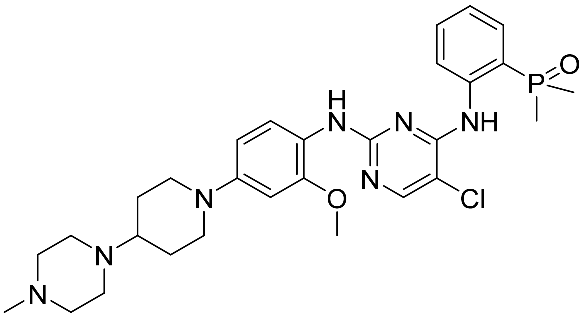
Cabozantinib	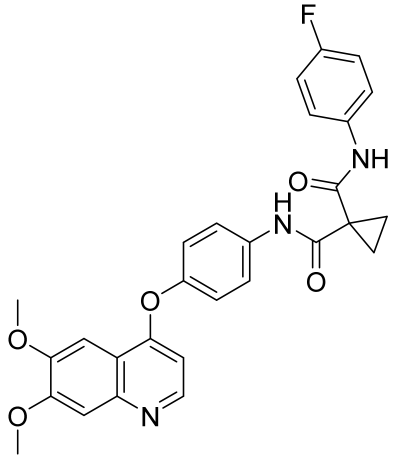
Capmatinib	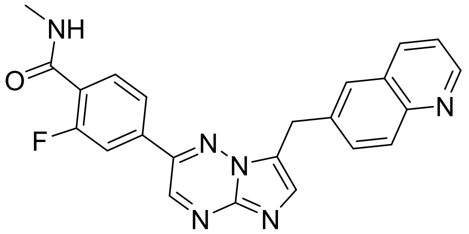
Ceritinib	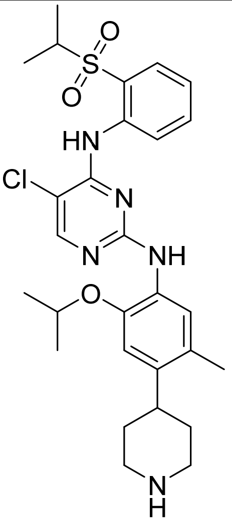

Crizotinib	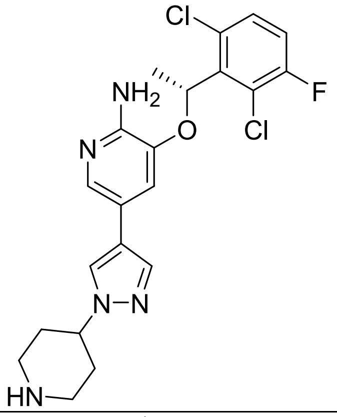
Dacomitinib	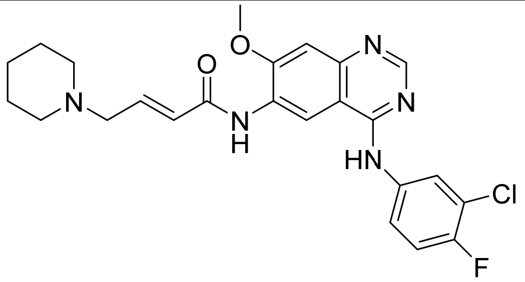
Dasatinib	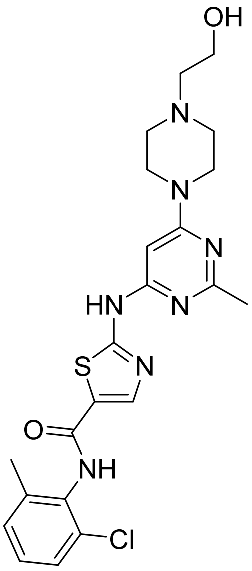
Entrectinib	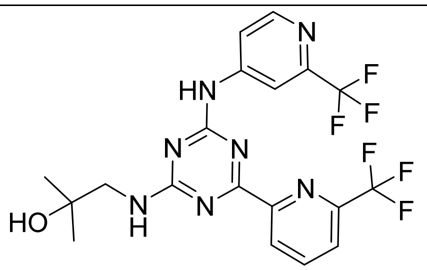
Erdafitinib	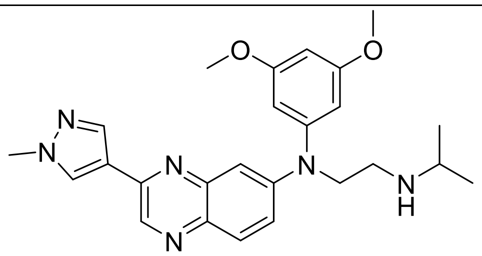

Erlotinib	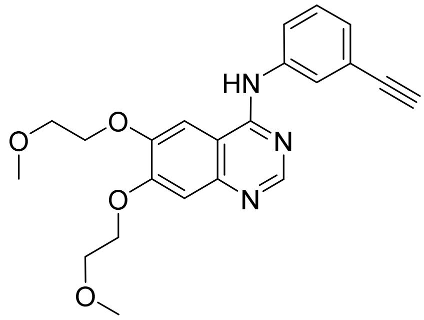
Fedratinib	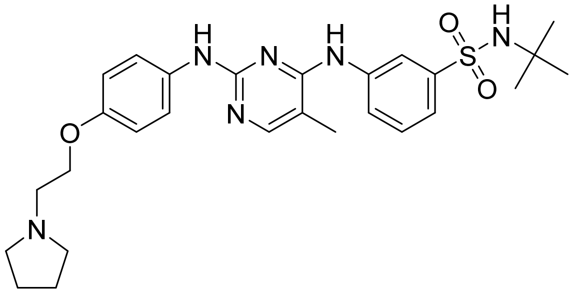
Fruquintinib	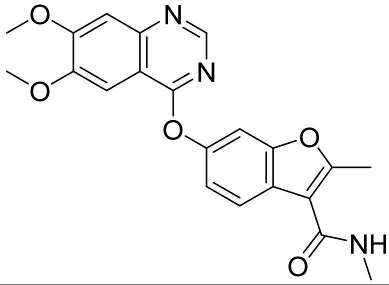
Gefitinib	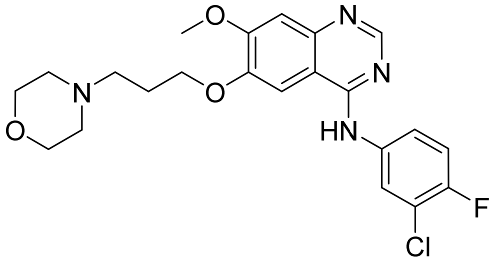
Ibrutinib	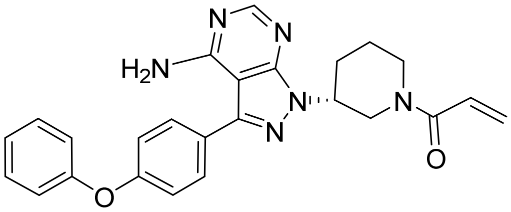
Icotinib	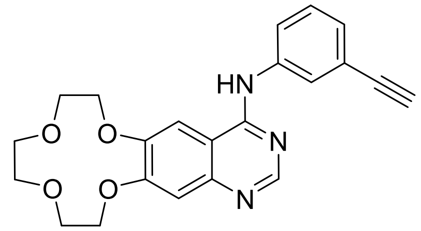
Imatinib	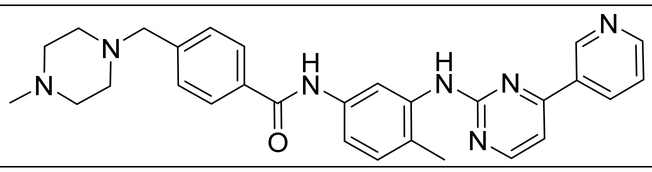

Lapatinib	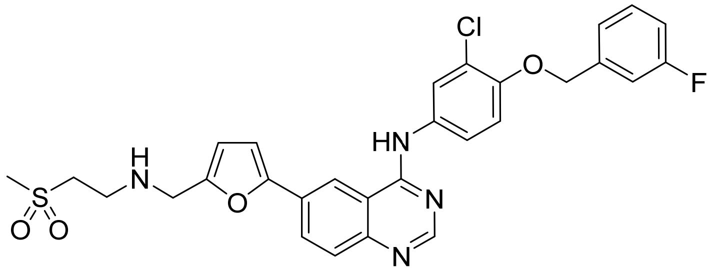
Lenwatinib	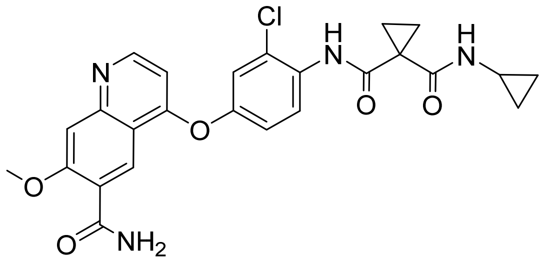
Lorlatinib	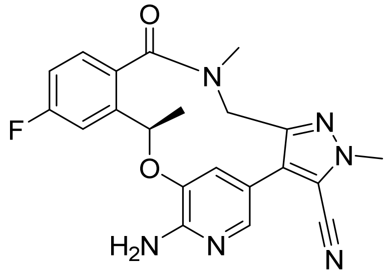
Momelotinib	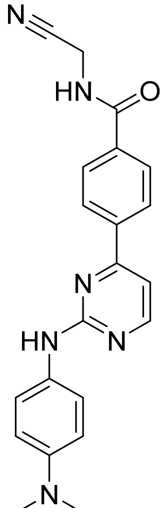
Neratinib	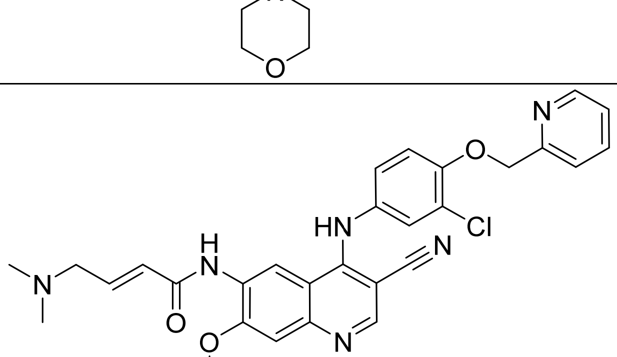

Nilotinib	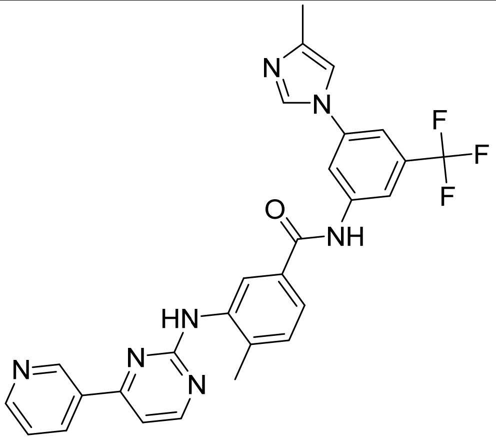
Osimertinib	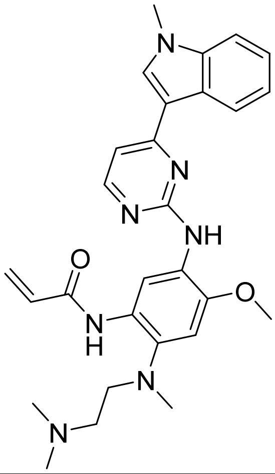
Pacritinib	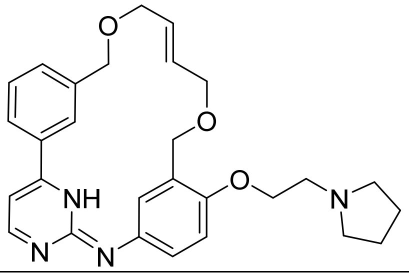
Pazopanib	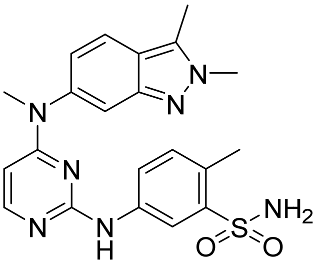
Pemigatinib	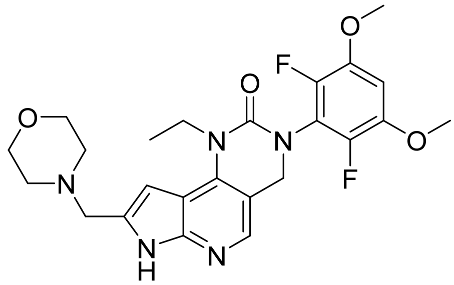

Ponatinib	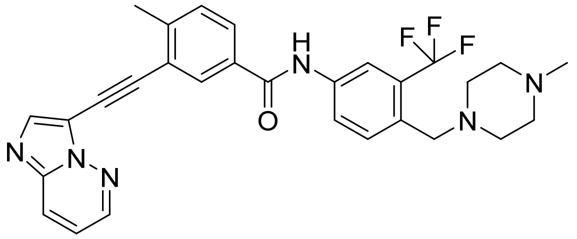
Pralsetinib	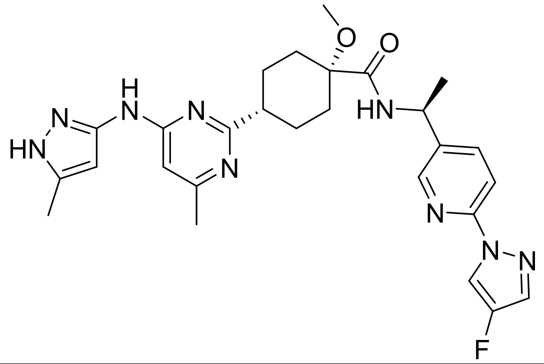
Pyrotinib	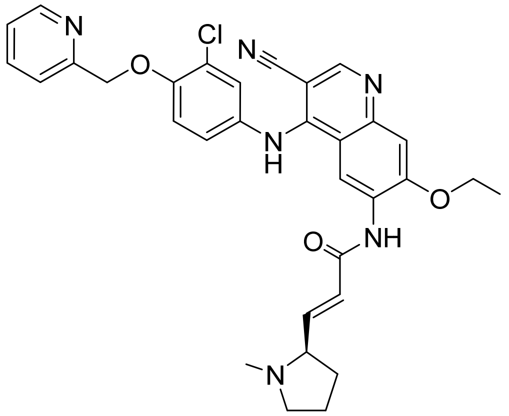
Regorafenib	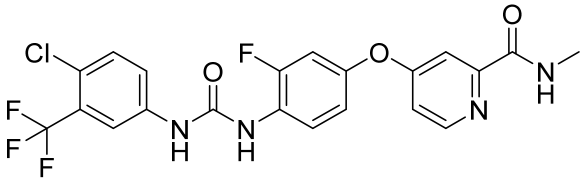
Ripretinib	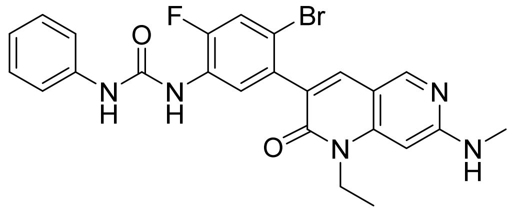
Ruxolitinib	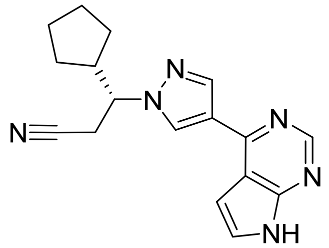
Savolitinib	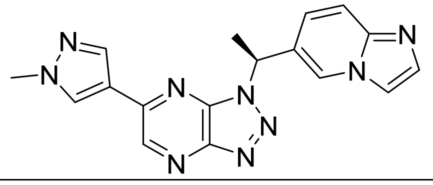

Selpercatinib	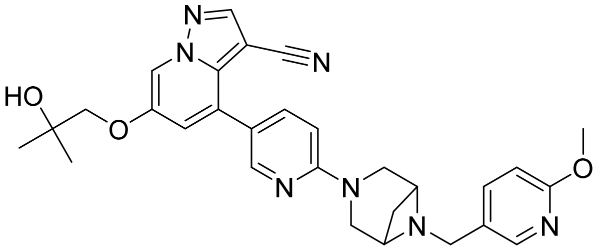
Sorafenib	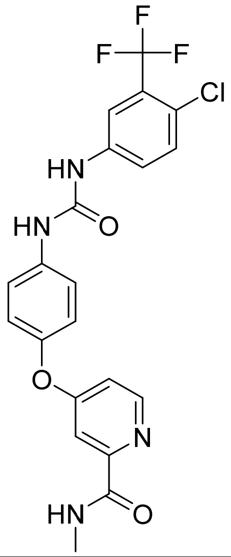
Sunitinib	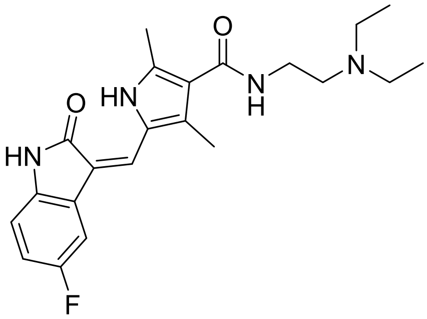
Tepotinib	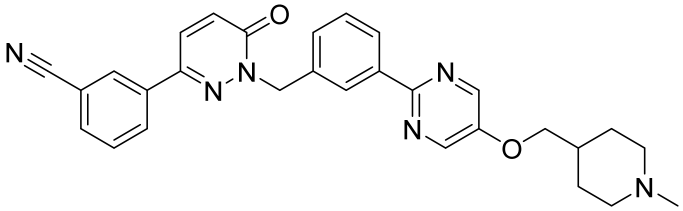
Tucatinib	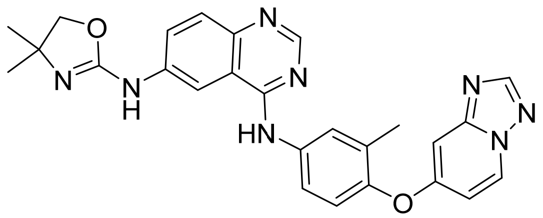

Vandetanib	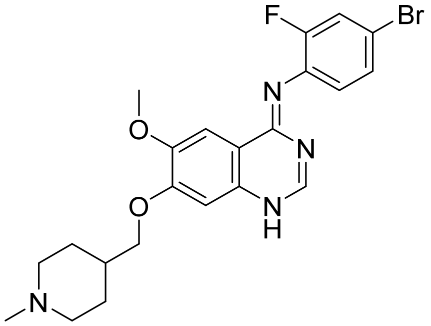
Zanubrutinib	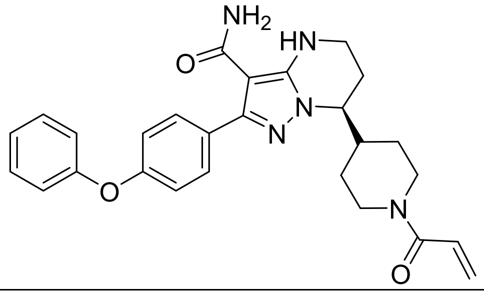

Table S2 Summary of selected small-molecule targeted drugs approved by the FDA in 2022-2025. Compound structures were retrieved from the ChEMBL database and then visualized and formatted for the manuscript.

**Small-molecule targeted drugs**	**Structures**
Adagrasib	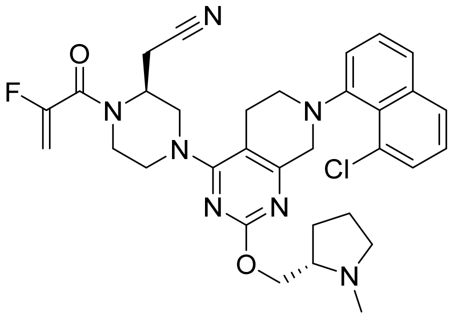
Alectinib	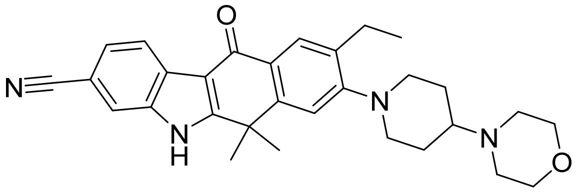
Alpelisib	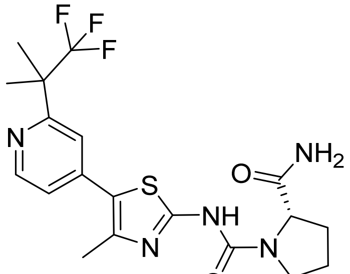
Avutometinib	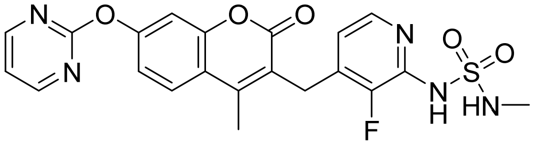

Belzutifan	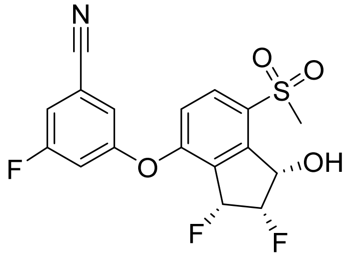
Capivasertib	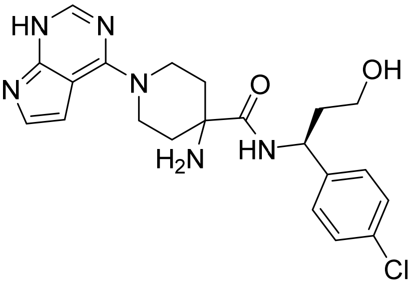
Capmatinib	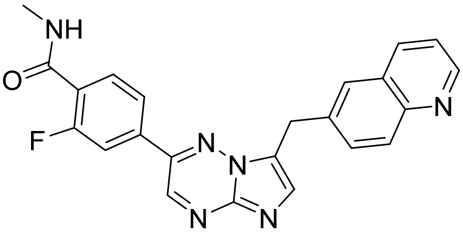
Dabrafenib	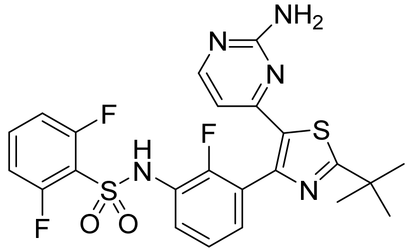
Defactinib	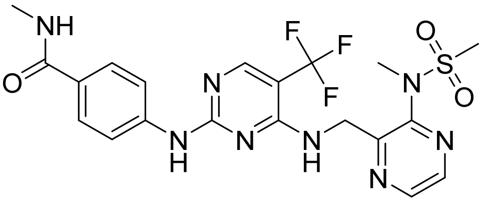
Erdafitinib	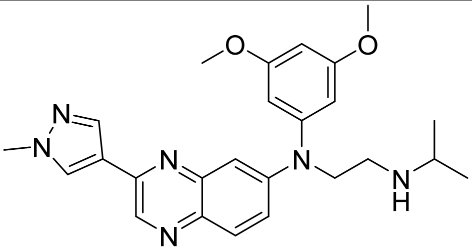
Ensartinib	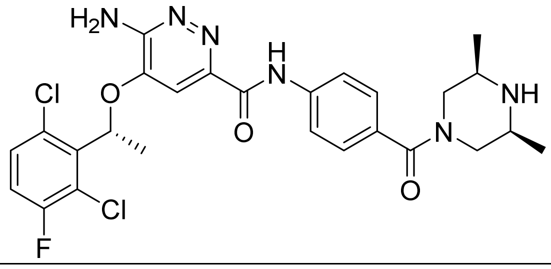

Fulvestrant	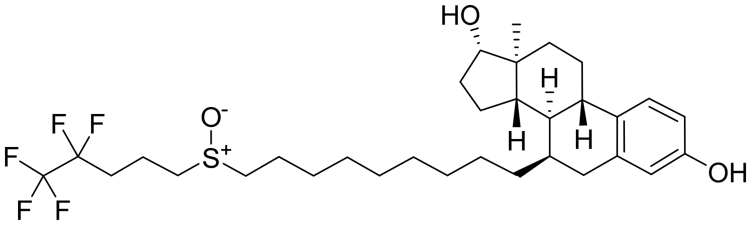
Futibatinib	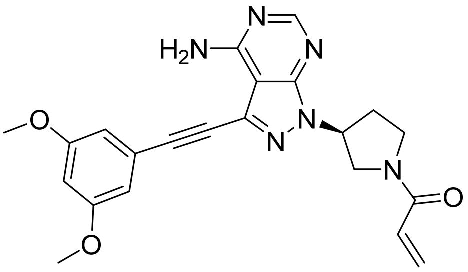
Imlunestrant	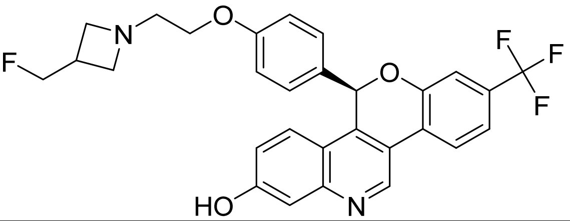
Inavolisib	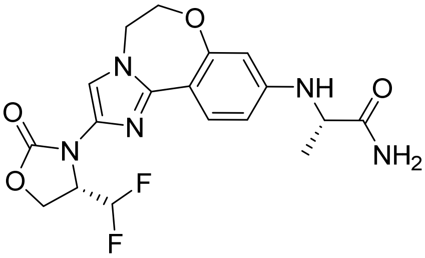
Lazertinib	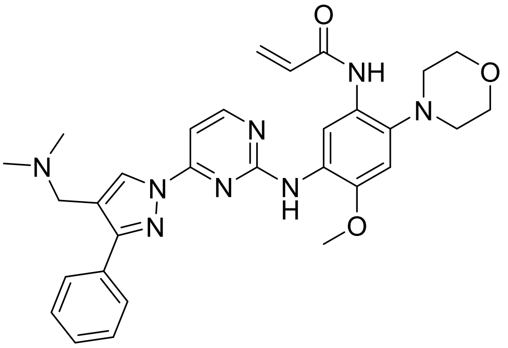
Niraparib	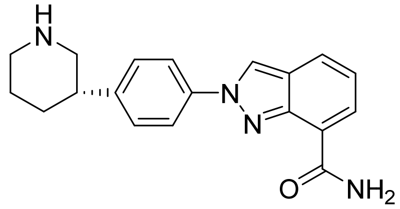
Nirogacestat	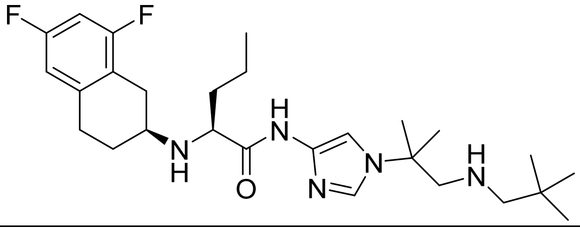

Olaparib	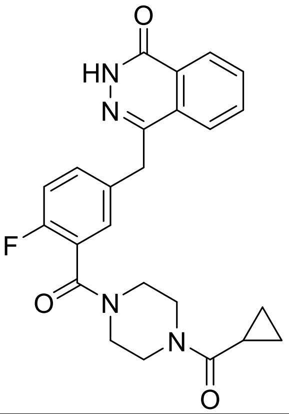
Olutasidenib	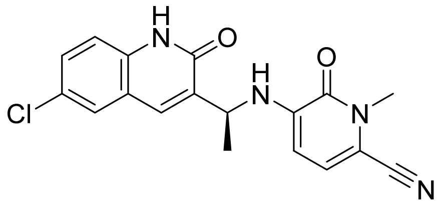
Osimertinib	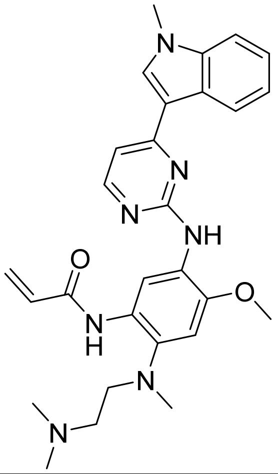
Pemigatinib	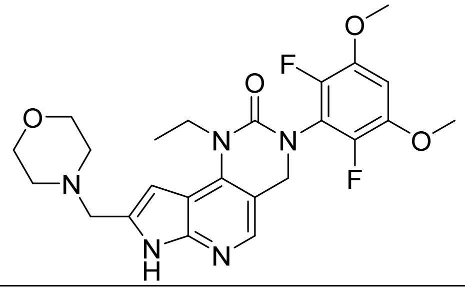
Pralsetinib	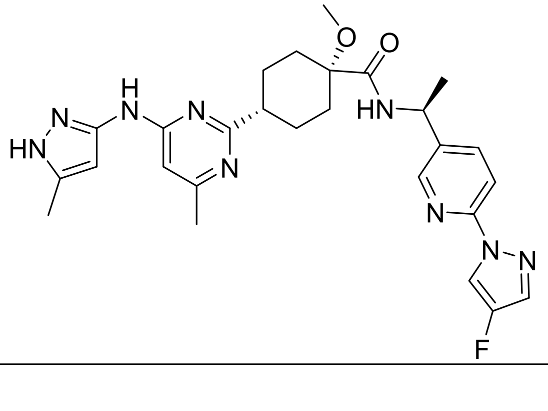

Quizartinib	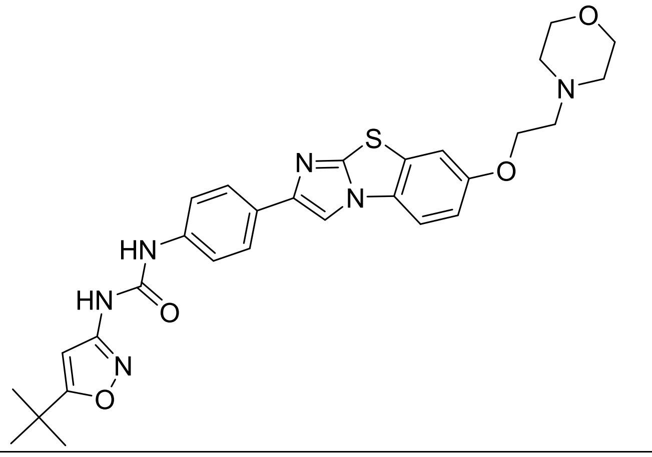
Repotrectinib	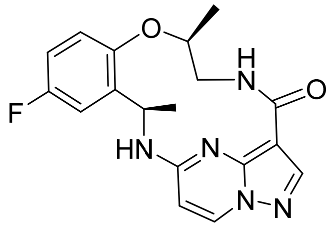
Selpercatinib	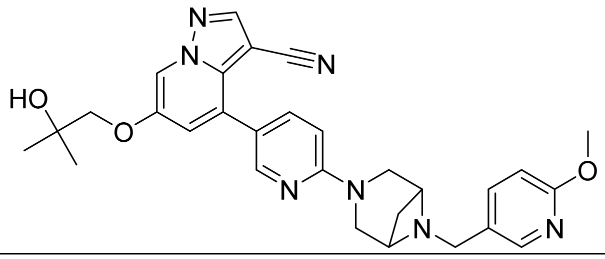
Sotorasib	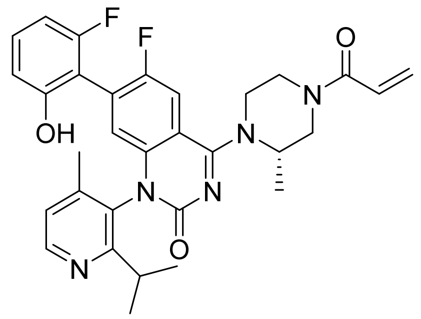
Sunvozertinib	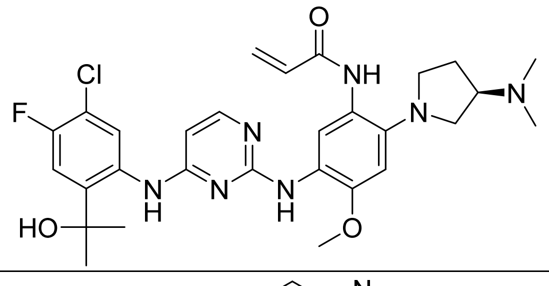
Taletrectinib	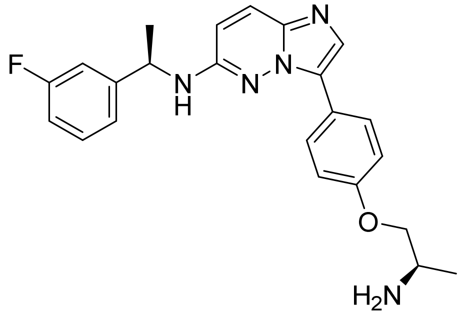

Talazoparib	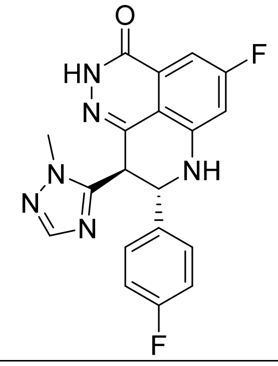
Tepotinib	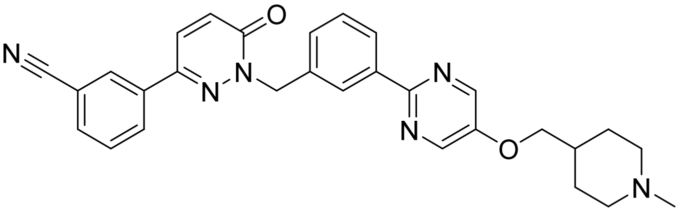
Toworafenib	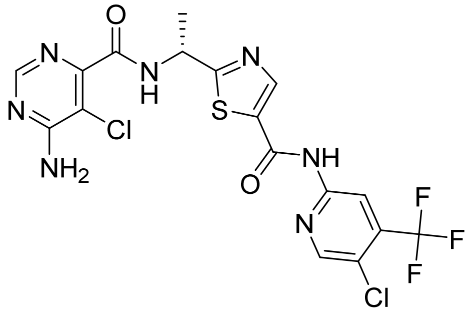
Trametinib	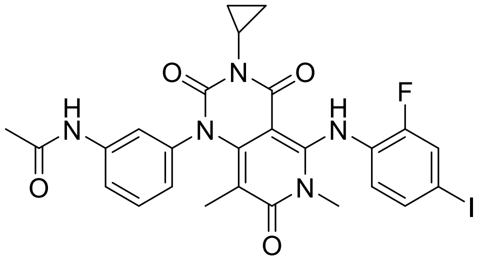
Vimseltinib	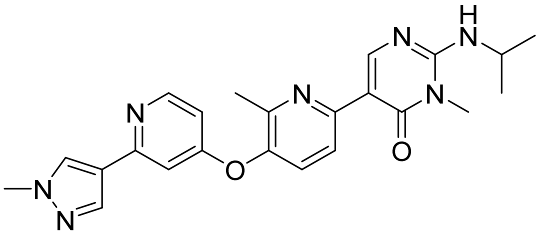

Vedotin	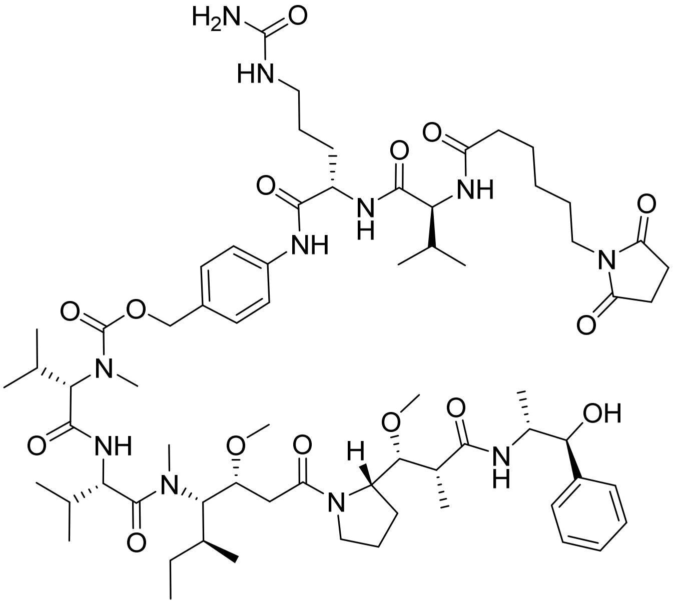
Vorasidenib	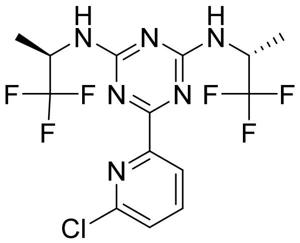

Table S3 Summary of selected targeted biologic drugs approved by the FDA in 2022-2025 (sources: Polish Society of Clinical Oncology; www.fda.gov).

Class	Molecular target	Drug name	Clinical indication	FDA approval date	Pivotal trial
**PD-1 inhibitors (monoclonal antibodies, mAbs)**	PD-1 expressed on T lymphocytes - blockade releases antitumor immune responses.	**Pembrolizumab** (Keytruda)	Neoadjuvant treatment in patients with resectable, locally advanced head and neck cancer with PD-L1-positive expression (CPS ≥ 1), with postoperative continuation in combination with radiotherapy, with or without cisplatin.	12.06.2025	KEYNOTE-689 [476]
			First-line treatment of unresectable advanced or metastatic pleural mesothelioma, in combination with pemetrexed and a platinum-based chemotherapy agent.	17.09.2024	KEYNOTE-483 [477]
			In patients with advanced or recurrent endometrial	17.06.2024	KEYNOTE-868 [478]

			cancer, in combination with carboplatin and paclitaxel.		
			In patients with cervical cancer FIGO stage III-IVA, in combination with concurrent chemoradiotherapy (radiotherapy plus chemotherapy).	12.01.2024	KEYNOTE-A18 [479]
		**Nivolumab (Opdivo, BMS)**	First-line treatment of unresectable or metastatic hepatocellular carcinoma, in combination with ipilimumab (Yervoy; an anti-CTLA-4 monoclonal antibody that blocks an inhibitory receptor on T-cell activation).	11.04.2025	CHECKMATE-9DW [480]
			In combination with ipilimumab (Yervoy; Bristol Myers Squibb) for adults and pediatric patients aged ≥12 years with unresectable or metastatic colorectal cancer with microsatellite instability-high (MSI-H) and/or deficient mismatch repair (dMMR).	08.04.2025	CHECKMATE-8HW [481]
			Neoadjuvant therapy in combination with platinum-based chemotherapy, followed after surgical resection by nivolumab monotherapy as adjuvant treatment in patients with resectable non-small cell lung cancer (tumor ≥ 4 cm and/or lymph node involvement) when EGFR mutation status and ALK rearrangement status have not been determined.	03.11.2024	CHECKMATE-77T [482]
			First-line treatment of unresectable or metastatic urothelial carcinoma, in combination with cisplatin and gemcitabine.	06.03.2024	CHECKMATE-901 [483]
			Adjuvant treatment in patients aged ≥12 years with cutaneous melanoma stage IIB-IIC following complete resection of the tumor.	13.10.2023	CHECKMATE-76K [484]

		**Dostarlimab-** dostarlimab-gxly (Jemperli, GSK)	Initial treatment in combination with carboplatin and paclitaxel, followed by continuation as dostarlimab-gxly monotherapy in patients with primary advanced or recurrent endometrial cancer with deficient mismatch repair (dMMR) and/or microsatellite instability-high (MSI-H).	01.08.2024	RUBY [485]
			In patients with advanced or recurrent endometrial cancer with deficient mismatch repair (dMMR), after prior platinum-based therapy, who are not candidates for curative local treatment.	09.02.2023	GARNET [486]
		**Cemiplimab-** rwlc (Libtayo, Regeneron)	In patients with advanced non-small cell lung cancer without EGFR, ALK, or ROS1 alterations, in combination with platinum-based chemotherapy.	08.11.2022	Study 16113 [487]
		**Toripalimab-tpzi** (Loqtorz, Coherus BioSciences)	In patients with metastatic or recurrent, locally advanced nasopharyngeal carcinoma, in combination with cisplatin and gemcitabine.	27.10.2023	JUPITER-02 [488]
		**Retifanlimab-diwr** (Zynyz, Incyte)	In patients with inoperable recurrent or metastatic squamous cell carcinoma of the anal canal, in combination with carboplatin and paclitaxel.	15.05.2025	POD1UM-303/InterAACT 2 [489]
			In patients with metastatic or locally advanced recurrent Merkel cell carcinoma.	22.03.2023 under the accelerated approval pathway	POD1UM-201 [490]
		**Penpulimab-kcqx** (Akeso Biopharma)	First-line treatment of recurrent or metastatic non-keratinizing nasopharyngeal carcinoma, in combination with cisplatin or carboplatin plus gemcitabine.	23.04.2025	AK105-304 [491]
**PD-L1 inhibitors (mAbs)**	PD-L1 expressed on tumor cells and antigen-presenting cells (APCs)	**Durvalumab** (Imfinzi)	In patients with locally advanced or metastatic biliary tract cancer (BTC), in combination	02.09.2022	TOPAZ-1 [492]

	blocks the interaction with PD-1.		with gemcitabine and cisplatin.		
		**Atezolizumab** SC (Tecentriq Hybreza)	In pediatric patients aged ≥2 years and adults with unresectable or metastatic alveolar soft part sarcoma (ASPS).	09.12.2022	ML39345 [493]
		**Axatilimab-csfr** (Niktimvo, Incyte)	In adults and pediatric patients weighing >40 kg for the treatment of chronic graft-versus-host disease (cGVHD) after failure of at least two prior lines of systemic therapy.	14.08.2024	AGAVE-201 [494]
		**Cosibelimab-ipdl** (Unloxcyt)	In patients with locally advanced or metastatic cutaneous squamous cell carcinoma when curative local treatment (surgery or radiotherapy) is not feasible.	13.12.2024	CK-301-101 [495]
**Bispecific antibodies**	Recruitment of T lymphocytes (CD3) to B cells (CD20)	**Epcoritamab-bysp** (Epkinly)	In patients with relapsed or refractory diffuse large B-cell lymphoma (DLBCL) or high-grade B-cell lymphoma (HGBL) after at least two prior lines of systemic therapy.	19.05.2023 under the accelerated approval pathway	EPCORE NHL-1 [496]
		**Glofitamab-gxbm** (Columvi)	In patients with relapsed or refractory diffuse large B-cell lymphoma, not otherwise specified (DLBCL, NOS), or other large B-cell lymphoma (LBCL) after at least two prior lines of therapy.	15.06.2023	NP30179 [497]
		**Mosunetuzumab -axgb** (Lunsumio)	In patients with relapsed or refractory follicular lymphoma after at least two prior lines of therapy.	23.12.2022 under the accelerated approval pathway	GO29781 [498]
**HER2-targeting bispecific antibodies**	Enhanced HER2 signaling blockade and Fc-dependent immune effector mechanisms.	**Zanidatamab-hrii** (Ziihera)	In previously treated patients with unresectable or metastatic biliary tract cancer with HER2 overexpression (HER2-positive disease).	20.11.2024	HERIZON-BTC-01 [499]
**CAR-T cell therapy**	Autologous T lymphocytes engineered to express a transgene encoding a chimeric antigen receptor (CAR) targeting CD19, BCMA, etc.	**Lisocabtagene maraleucel** (Breyanzi)	Second-line treatment in patients with large B-cell lymphoma (LBCL).	24.06.2022	TRANSFORM [500]
			In patients with relapsed or refractory mantle cell lymphoma (MCL) after at least two prior lines of therapy.	30.05.2024	TRANSCEND-MCL [501]
			In patients with relapsed or refractory follicular lymphoma (FL).	15.05.2024	TRANSCEND-FL [502]

<table>
<tr><td rowspan="4"></td><td rowspan="4"></td><td>Axicabtagene ciloleucel (Yescarta)</td><td>In patients with relapsed large B-cell lymphoma (LBCL).</td><td>01.04.2022</td><td>ZUMA-7 [503]</td></tr>
<tr><td>Tisagenlecleucel (Kymriah)</td><td>In patients with relapsed or refractory follicular lymphoma (FL) after at least two prior lines of therapy.</td><td>27.05.2022 under the accelerated approval pathway</td><td>ELARA [504]</td></tr>
<tr><td>Ciltacabtagene autoleucel (Carvykti)</td><td>In patients with relapsed or refractory multiple myeloma after at least four prior lines of therapy.</td><td>28.02.2022</td><td>CARTITUDE-1 [505]</td></tr>
<tr><td>Afamitresgene autoleucel (Tecelra)</td><td>In patients with unresectable or metastatic synovial sarcoma with MAGE-A4-positive expression.</td><td>02.08.2024</td><td>SPEARHEAD [506]</td></tr>
<tr><td>Cell therapy using tumor-infiltrating lymphocytes (TILs).</td><td>Polyclonal T lymphocytes recognizing tumor neoantigens, thereby enhancing antitumor immune responses.</td><td>Lifileucel (Amtagvi, Iovance)</td><td>Advanced or metastatic melanoma after prior anti-PD-1 immunotherapy.</td><td>16.02.2024 under the accelerated approval pathway</td><td>C-144-01 [507]</td></tr>
<tr><td>TCR-based therapy/adoptive T-cell therapy.</td><td>A fusion T-cell receptor (TCR) protein specific for gp100 presented in the HLA-A02:01 complex, linked to an anti-CD3 moiety, which recruits T lymphocytes to uveal melanoma cells.</td><td>Tebentafusp-tebn (Kimmtrak)</td><td>In patients with unresectable or metastatic uveal melanoma.</td><td>25.01.2022</td><td>IMCgp100-202 [508]</td></tr>
<tr><td rowspan="3">ADCs (antibody-drug conjugates) - cytotoxic antibody conjugates.</td><td rowspan="3">An antibody directed against a tumor antigen plus a linker and a cytotoxic payload (e.g., DM4, MMAE, a topoisomerase I inhibitor).</td><td rowspan="2">Datopotamab deruxtecan (Datroway)</td><td>In patients with unresectable or metastatic hormone receptor-positive (HR-positive) and human epidermal growth factor receptor 2-negative (HER2-negative) breast cancer, after prior endocrine therapy and chemotherapy.</td><td>17.01.2025</td><td>TROPION-Breast01 [509]</td></tr>
<tr><td>In patients with locally advanced or metastatic non-small cell lung cancer with EGFR alterations (EGFR-positive disease), after prior EGFR inhibitor therapy and platinum-based chemotherapy.</td><td>23.06.2025</td><td>TROPION-Lung01 TROPION-Lung05 [510]</td></tr>
<tr><td>Mirvetuximab soravtansine-gynx (Elahere)</td><td>In patients with platinum-resistant ovarian cancer, fallopian tube cancer,</td><td>14.11.2022</td><td>Study 0417 [511]</td></tr>
</table>

			or primary peritoneal cancer with folate receptor alpha (FRα)-positive expression, after at least three prior lines of systemic therapy.		
		**Enfortumab vedotin-ejfv** (Padcev – Nectin)	In patients with locally advanced or metastatic urothelial carcinoma.	15.12.2023	EV-302/KN-A39 [512]
		**Tisotumab vedotin-tftv** (Tivdak)	In patients with recurrent or metastatic cervical cancer after prior chemotherapy.	28.04.2024	InnovaTV301 [513]
		**Polatuzumab vedotin-piiq** (Polivy)	First-line treatment of diffuse large B-cell lymphoma, not otherwise specified (DLBCL, NOS), and high-grade B-cell lymphoma (HGBL) with an International Prognostic Index (IPI) score ≥ 2, in combination with the R-CHP regimen (rituximab, cyclophosphamide, doxorubicin, and prednisone).	19.04.2023	POLARIX [514]
		**Telisotuzumab vedotin-tllv** (Emrelis)	In patients with locally advanced or metastatic non-squamous non-small cell lung cancer with high c-MET expression (≥50%).	14.05.2025	LUMINOSITY [515]
		**Trastuzumab deruxtecan** (fam-/Enhertu)	In patients with unresectable or metastatic HER2-overexpressing tumors (HER2-positive disease) after prior systemic therapy.	05.04.2024	DESTINY-Lung01 [516]
			In patients with unresectable or metastatic non-small cell lung cancer with HER2 overexpression (HER2-positive disease).	11.08.2022	DESTINY-Lung02 [517]
			For unresectable or metastatic HR-positive, HER2-low breast cancer after progression on at least one line of endocrine therapy for metastatic disease.	27.01.2025	DESTINY-Breast06 [518]

**Bispecific /other mAb directed against tumor antigens.**	Various targets: CD38, CLDN18.2.	**Isatuximab-irfc** (Sarclisa)	In patients with newly diagnosed multiple myeloma who are ineligible for stem cell transplantation, in combination with bortezomib, lenalidomide, and dexamethasone.	20.09.2024	IMROZ [519]
		**Zolbetuximab-clzb** (Vyloy)	First-line treatment of locally advanced unresectable or metastatic gastric or gastroesophageal junction adenocarcinoma, in combination with fluoropyrimidine- and platinum-based chemotherapy, in patients without HER2 overexpression (HER2-negative disease) and with CLDN18.2-positive expression.	18.10.2024	SPOTLIGHT/GLOW [520]
		**Talquetamab-tgvs** (Talvey)	In patients with relapsed or refractory multiple myeloma after at least four prior lines of therapy.	09.08.2023	MMY1001 [521]
**Gene immunotherapy/cytokines/ mesenchymal stromal cells.**	Tumor microenvironment modulation: IL-15/IFN expression, NK/T-cell stimulation, and inflammation modulation.	**Nadofaragene firadenovec-vncg** (Adstiladrin)	In patients with bacillus Calmette-Guerin (BCG)-unresponsive non-muscle-invasive bladder cancer (NMIBC) with carcinoma in situ (CIS), with or without papillary tumors.	16.12.2022	CS-003 [522]
		**Nogapendekin alfa inbakicept-pmln** (Anktiva)	In patients with non-muscle-invasive bladder cancer (NMIBC), in combination with intravesical bacillus Calmette-Guerin (BCG) immunotherapy.	22.04.2024	QUILT-3.032 [523]
		**Remestemcel-L-rknd** (Ryoncil)	In pediatric patients aged ≥2 months with steroid-refractory acute graft-versus-host disease following allogeneic hematopoietic stem cell transplantation.	18.12.2024	MSB-GVHD001 [524]
**Oligonucleotide-based therapy.**	The template region of the RNA component of human telomerase.	**Imetelstat** (Rytelo)	In patients with low- or intermediate-risk myelodysplastic syndromes (MDS) with red blood cell transfusion-dependent anemia.	06.06.2024	IMerge [525]

476. Research, C. for D.E. and FDA approves neoadjuvant and adjuvant pembrolizumab for resectable locally advanced head and neck squamous cell carcinoma. FDA **2025**.
477. Research, C. for D.E. and FDA approves pembrolizumab with chemotherapy for unresectable advanced or metastatic malignant pleural mesothelioma. FDA **2024**.
478. Research, C. for D.E. and FDA approves pembrolizumab with chemotherapy for primary advanced or recurrent endometrial carcinoma. FDA **2024**.
479. Research, C. for D.E. and FDA approves pembrolizumab with chemoradiotherapy for FIGO 2014 Stage III-IVA cervical cancer. FDA **2024**.
480. Research, C. for D.E. and FDA approves nivolumab with ipilimumab for unresectable or metastatic hepatocellular carcinoma. FDA **2025**.
481. Research, C. for D.E. and FDA approves nivolumab with ipilimumab for unresectable or metastatic MSI-H or dMMR colorectal cancer. FDA **2025**.
482. Research, C. for D.E. and FDA approves neoadjuvant/adjuvant nivolumab for resectable non-small cell lung cancer. FDA **2024**.
483. Research, C. for D.E. and FDA approves nivolumab in combination with cisplatin and gemcitabine for unresectable or metastatic urothelial carcinoma. FDA **2024**.
484. Research, C. for D.E. and FDA approves nivolumab for adjuvant treatment of Stage IIB/C melanoma. FDA **2023**.
485. Research, C. for D.E. and FDA expands endometrial cancer indication for dostarlimab-gxly with chemotherapy. FDA **2024**.
486. Research, C. for D.E. and FDA grants regular approval to dostarlimab-gxly for dMMR endometrial cancer. FDA **2023**.
487. Research, C. for D.E. and FDA approves cemiplimab-rwlc in combination with platinum-based chemotherapy for non-small cell lung cancer. FDA **2024**.
488. Research, C. for D.E. and FDA approves toripalimab-tpzi for nasopharyngeal carcinoma. FDA **2024**.
489. Research, C. for D.E. and FDA approves retifanlimab-dlwr with carboplatin and paclitaxel and as a single agent for squamous cell carcinoma of the anal canal. FDA **2025**.
490. Research, C. for D.E. and FDA grants accelerated approval to retifanlimab-dlwr for metastatic or recurrent locally advanced Merkel cell carcinoma. FDA **2024**.
491. Research, C. for D.E. and FDA approves penpulimab-kcqx for non-keratinizing nasopharyngeal carcinoma. FDA **2025**.
492. Research, C. for D.E. and FDA approves durvalumab for locally advanced or metastatic biliary tract cancer. FDA **2024**.
493. Research, C. for D.E. and FDA grants approval to atezolizumab for alveolar soft part sarcoma. FDA **2022**.
494. Research, C. for D.E. and FDA approves axatilimab-csfr for chronic graft-versus-host disease. FDA **2024**.
495. Research, C. for D.E. and FDA approves cosibelimab-ipdl for metastatic or locally advanced cutaneous squamous cell carcinoma. FDA **2024**.
496. Research, C. for D.E. and FDA grants accelerated approval to epcoritamab-bysp for relapsed or refractory diffuse large B-cell lymphoma and high-grade B-cell lymphoma. FDA **2023**.
497. Research, C. for D.E. and FDA grants accelerated approval to glofitamab-gxbm for selected relapsed or refractory large B-cell lymphomas. FDA **2023**.
498. Research, C. for D.E. and FDA grants accelerated approval to mosunetuzumab-axgb for relapsed or refractory follicular lymphoma. FDA **2024**.
499. Research, C. for D.E. and FDA grants accelerated approval to zanidatamab-hrii for previously treated unresectable or metastatic HER2-positive biliary tract cancer. FDA **2024**.
500. Research, C. for D.E. and FDA approves lisocabtagene maraleucel for second-line treatment of large B-cell lymphoma. FDA **2024**.
501. Research, C. for D.E. and FDA approves lisocabtagene maraleucel for relapsed or refractory mantle cell lymphoma. FDA **2024**.

502. Research, C. for D.E. and FDA grants accelerated approval to lisocabtagene maraleucel for follicular lymphoma. FDA **2024**.
503. Research, C. for D.E. and FDA approves axicabtagene ciloleucel for second-line treatment of large B-cell lymphoma. FDA **2024**.
504. Research, C. for D.E. and FDA approves tisagenlecleucel for relapsed or refractory follicular lymphoma. FDA **2024**.
505. Research, C. for D.E. and FDA approves ciltacabtagene autoleucel for relapsed or refractory multiple myeloma. FDA **2024**.
506. Research, C. for D.E. and FDA grants accelerated approval to afamitresgene autoleucel for unresectable or metastatic synovial sarcoma. FDA **2024**.
507. Research, C. for D.E. and FDA grants accelerated approval to lifileucel for unresectable or metastatic melanoma. FDA **2024**.
508. Research, C. for D.E. and FDA approves tebentafusp-tebn for unresectable or metastatic uveal melanoma. FDA **2024**.
509. Research, C. for D.E. and FDA approves datopotamab deruxtecan-dlnk for unresectable or metastatic, HR-positive, HER2-negative breast cancer. FDA **2025**.
510. Research, C. for D.E. and FDA grants accelerated approval to datopotamab deruxtecan-dlnk for EGFR-mutated non-small cell lung cancer. FDA **2025**.
511. Research, C. for D.E. and FDA grants accelerated approval to mirvetuximab soravtansine-gynx for FRα positive, platinum-resistant epithelial ovarian, fallopian tube, or peritoneal cancer. FDA **2024**.
512. Research, C. for D.E. and FDA approves enfortumab vedotin-ejfv with pembrolizumab for locally advanced or metastatic urothelial cancer. FDA **2024**.
513. Research, C. for D.E. and FDA approves tisotumab vedotin-tftv for recurrent or metastatic cervical cancer. FDA **2024**.
514. Research, C. for D.E. and FDA approves polatuzumab vedotin-piiq for previously untreated diffuse large B-cell lymphoma, not otherwise specified, and high-grade B-cell lymphoma. FDA **2024**.
515. Research, C. for D.E. and FDA grants accelerated approval to telisotuzumab vedotin-tllv for NSCLC with high c-Met protein overexpression. FDA **2025**.
516. Research, C. for D.E. and FDA grants accelerated approval to fam-trastuzumab deruxtecan-nxki for unresectable or metastatic HER2-positive solid tumors. FDA **2024**.
517. Research, C. for D.E. and FDA grants accelerated approval to fam-trastuzumab deruxtecan-nxki for HER2-mutant non-small cell lung cancer. FDA **2024**.
518. Research, C. for D.E. and FDA approves fam-trastuzumab deruxtecan-nxki for unresectable or metastatic HR-positive, HER2-low or HER2-ultralow breast cancer. FDA **2025**.
519. Research, C. for D.E. and FDA approves isatuximab-irfc with bortezomib, lenalidomide, and dexamethasone for newly diagnosed multiple myeloma. FDA **2024**.
520. Research, C. for D.E. and FDA approves zolbetuximab-clzb with chemotherapy for gastric or gastroesophageal junction adenocarcinoma. FDA **2024**.
521. Research, C. for D.E. and FDA grants accelerated approval to talquetamab-tgvs for relapsed or refractory multiple myeloma. FDA **2024**.
522. Research, C. for D.E. and FDA approves first adenoviral vector-based gene therapy for high-risk Bacillus Calmette-Guérin unresponsive non-muscle invasive bladder cancer. FDA **2022**.
523. Research, C. for D.E. and FDA approves nogapendekin alfa inbakicept-pmln for BCG-unresponsive non-muscle invasive bladder cancer. FDA **2024**.
524. Research, C. for D.E. and FDA approves remestemcel-L-rknd for steroid-refractory acute graft versus host disease in pediatric patients. FDA **2024**.
525. Research, C. for D.E. and FDA approves imetelstat for low- to intermediate-1 risk myelodysplastic syndromes with transfusion-dependent anemia. FDA **2024**.
